# Integration of summary data from GWAS and eQTL studies identified novel risk genes for coronary artery disease

**DOI:** 10.1097/MD.0000000000024769

**Published:** 2021-03-19

**Authors:** Yigang Zhong, Liuying Chen, Jingjing Li, Yinghao Yao, Qiang Liu, Kaimeng Niu, Yunlong Ma, Yizhou Xu

**Affiliations:** a Department of Cardiology, Affiliated Hangzhou First People's Hospital, Zhejiang University School of Medicine; b Zhejiang Chinese Medical University; c State Key Laboratory for Diagnosis and Treatment of Infectious Diseases, The First Affiliated Hospital, Collaborative Innovation Center for Diagnosis and Treatment of Infectious Diseases, Zhejiang University School of Medicine, Hangzhou; d Institute of Biomedical Big Data, School of Ophthalmology & Optometry and Eye Hospital, Wenzhou Medical University, Wenzhou, China.

**Keywords:** Coronary artery disease, expression quantitative trait loci, genome-wide association study, pathways, single nucleotide polymorphisms

## Abstract

Several genetic loci have been reported to be significantly associated with coronary artery disease (CAD) by multiple genome-wide association studies (GWAS). Nevertheless, the biological and functional effects of these genetic variants on CAD remain largely equivocal. In the current study, we performed an integrative genomics analysis by integrating large-scale GWAS data (N = 459,534) and 2 independent expression quantitative trait loci (eQTL) datasets (N = 1890) to determine whether CAD-associated risk single nucleotide polymorphisms (SNPs) exert regulatory effects on gene expression. By using Sherlock Bayesian, MAGMA gene-based, multidimensional scaling (MDS), functional enrichment, and in silico permutation analyses for independent technical and biological replications, we highlighted 4 susceptible genes (*CHCHD1*, *TUBG1, LY6G6C*, and *MRPS17*) associated with CAD risk. Based on the protein–protein interaction (PPI) network analysis, these 4 genes were found to interact with each other. We detected a remarkably altered co-expression pattern among these 4 genes between CAD patients and controls. In addition, 3 genes of *CHCHD1* (*P* = .0013), *TUBG1* (*P* = .004), and *LY6G6C* (*P* = .038) showed significantly different expressions between CAD patients and controls. Together, we provide evidence to support that these identified genes such as *CHCHD1* and *TUBG1* are indicative factors of CAD.

## 1 Introduction

Coronary artery disease (CAD) is one of the leading causes of mortality and morbidity worldwide.^[1,2]^ Despite the advanced developments in prevention and treatment, the healthcare and economic burden of CAD remains high. CAD is highly influenced by both genetic and environmental determinants.^[3,4]^ The narrow-sense heritability of CAD has been estimated to be approximately 50%.^[5,6]^ Thus, identifying the genetic determinants with critical roles in the pathogenesis of CAD is critical for proposing novel therapeutic targets.

In the past decade, CAD has been a focus of genetics-based or genomics-based studies. Among these, genome-wide association study (GWAS) has been extensively applied to discover CAD-associated genetic loci. To date, more than 160 loci have been reported to be associated with CAD.^[7]^ This rapid advance has been largely attributable to the release of genome-wide genotyping data of the UK Biobank study together with existing GWAS from the CARDIoGRAMplusC4D consortium. For example, the chromosome region of 9p21 was reported to be the highest risk region associated with CAD.^[8–10]^ Furthermore, numerous GWASs based on a large number of samples have documented single nucleotide polymorphisms (SNPs) to be associated with a group of CAD-related risk factors, including low-density lipoprotein cholesterol,^[11]^ high-density lipoprotein cholesterol,^[11]^ diastolic blood pressure,^[12]^ systolic blood pressure,^[12]^ triglycerides,^[11]^ type II diabetes,^[13]^ waist-to-hip ratio,^[14]^ and body mass index.^[15]^ Recently, a study reported^[16]^ a significant genetic correlation between CAD and other lipid metabolism-related traits (*P* value <1 × 10^−16^), and 13 genes (e.g., *LPA*, *APOE*, *APOC1*, and *SLC22A3*) were identified as common risk factors between CAD and plasma lipid levels. However, despite GWAS studies, the biological effects of significant genetic variants on CAD remain largely unknown.

Moreover, the GWAS method employed the stringent genome-wide significance threshold to avoid false discoveries due to simultaneous testing of the associations of millions of SNPs; also, to a large number of SNPs might have weak genetic associations, and hence, not identified in the current sample sizes. Furthermore, evidence from previous GWASs have shown that the vast majority of identified SNPs are mapped into non-coding genomic regions.^[17]^ Thus, it can be speculated that these SNPs affect the expression level of specific gene rather than the function of its protein. Genetic variants can influence the expression level of RNA via *cis*- or *trans*-regulatory mechanisms or both.^[18]^ Accumulating evidence also supported that the dysfunctional expression of susceptible genes play a vital role in the etiology of complex diseases.^[19–21]^ Therefore, additional studies are required to discover the underlying regulatory functions of these SNPs with small-to-moderate effects on CAD, which potentially contribute to understanding the missing heritability of CAD.

Previous studies have focused on the integration of GWAS summary statistics with expression quantitative trait loci (eQTL) data to reveal susceptible genes associated with a complex array of diseases due to pleiotropy.^[22–24]^ For example, Zhu et al^[25]^ utilized GWAS summary data (N = 339,224) and eQTL data (N = 5311) obtained by summary data-based Mendelian randomization (SMR) method and prioritized 126 susceptible genes, of which 25 were newly identified; for example, *NMRAL1* and *SNX19* for schizophrenia and *ANKRD55* and *TRAF1* for rheumatoid arthritis. Furthermore, He et al^[22]^ proposed a Bayesian-based inference method (also called Sherlock) to systematically discover the *cis-* and *trans*-regulatory effects of SNPs on the expression levels of disease-risk genes by incorporating GWAS summary and eQTL datasets. By conducting an integrative genomics analysis based on GWAS, eQTL, and mQTL data, our group^[26]^ have reported 34 important genes with numerious candidate SNPs conffering risk to the comorbidity of schizophrenia and smoking behaviors. By combining different layers of evidence, many novel genes, which were hard to be identified by a GWAS alone, were identified for complex diseases, including gout disease,^[27]^ schizophrenia,^[28]^ and major depressive disorders.^[29,30]^

In the current study, we conducted a comprehensive genomics analysis using the Sherlock Bayesian method to integrate a large-scale GWAS summary dataset (N = 459,534) with 2 independent eQTL datasets (N = 1890). The primary goal of the current study was to determine whether risk SNPs influenced the expression levels of genes and identify CAD-associated susceptible genes. Furthermore, we performed several bioinformatics analyses using multi-omics data to highlight the CAD-risk genes.

## 2 Materials and methods

### 2.1 Summary on design of current study

**Figure 1:**
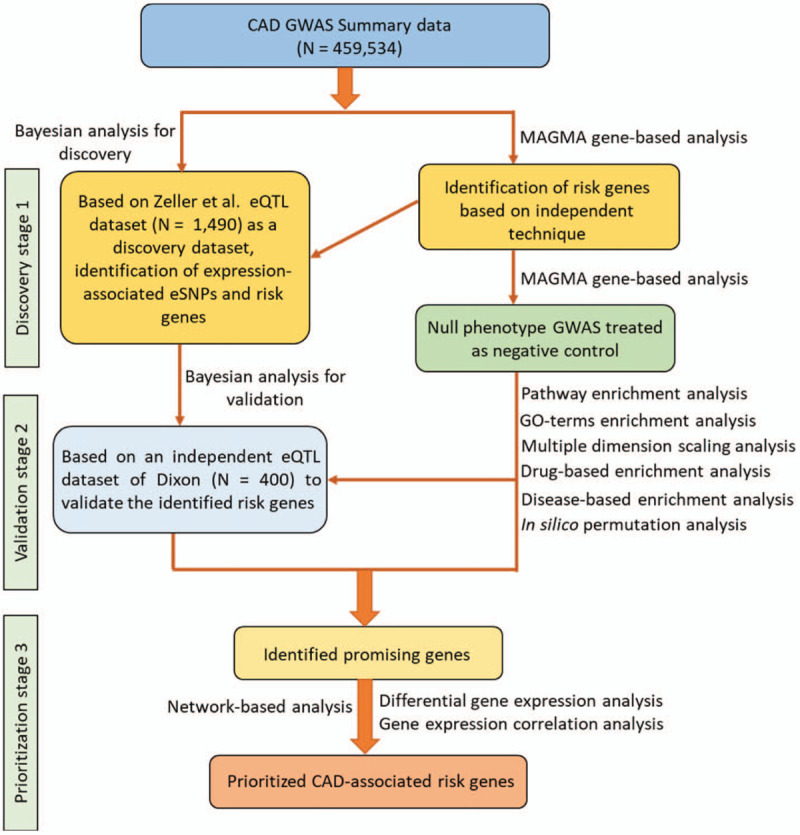
Schematic of current genomics analysis.

In the current comprehensive genomics study, we designed a three-stage in silico analysis framework (see Fig. 1). In the first discovery stage, we used the Sherlock tool to integrate a large-scale GWAS summary statistics dataset on CAD with a large eQTL dataset for identifying CAD-associated risk genes. In the second validation stage, we reperformed the Sherlock Bayesian analysis using an independent eQTL dataset to replicate these identified genes in the discovery stage. Meanwhile, we also used the MAGMA tool to perform a genome-wide gene-based association analysis as an independent method to validate the Sherlock-identified genes. To avoid random events, we further simulated a null trait as a negative control. In the third prioritization stage, we conducted a series of bioinformatics analyses, including pathway/GO-term enrichment analysis, multiple dimension scaling analysis, drug-based enrichment analysis, disease-based enrichment analysis, in silico permutation analysis, network-based analysis, differential gene expression analysis, and gene co-expression analysis, to prioritize the important risk genes implicated in CAD.

### 2.2 Dataset #1 for GWAS summary data on CAD

To identify candidate causal genes for CAD using an integrative genomics analysis, we downloaded the GWAS meta-analysis summary data (N = 459,534) on CAD^[31]^ from the CARDIoGRAMplusC4D Consortium website. This sample set contains 120,419 CAD patients and 339,115 matched controls. All subjects provided written consent for participating in the GWAS study that was approved by the local research ethics committee or institutional review board. Basic clinical information on CAD patients and controls were reported in the original study.^[31]^

### 2.3 Dataset #2 for GWAS summary data on random phenotype (fake CAD)

To avoid the confusion of random events, as refer to the method of a previous study,^[32]^ we constructed a fake CAD-based GWAS summary dataset based on a published GWAS on lung cancer with 3960 samples, as reported by Landi et al.^[33]^ For these individuals, the disease status was randomly defined using the function of RANDBETWEEN (“CAD,” “CONTROL”) in Microsoft Excel. We used “CAD” to represent CAD patients, and “CONTROL” to represent non-CAD controls. The randomly distributed CAD phenotype was defined as null phenotype. The logistic regression model, using PLINK (version 1.07), was employed to analyze the GWAS dataset on null phenotype.^[34]^

### 2.4 Dataset #3 for eQTL data as the discovery dataset

This eQTL dataset, reported by Zeller et al^[35]^ provides in-depth insights into the overall variability of gene expression. A total of 1490 unrelated participants were enrolled from a single-center Gutenberg Heart Study. The RNA and DNA samples were isolated from circulating monocytes in these participants. After rigorous quality control, a total of 675,359 SNPs and 12,808 genes were included in the eQTL analysis. In the current study, we first integrated this eQTL dataset as a discovery dataset with GWAS on CAD for Sherlock analysis to identify the common candidate genes with expression-associated SNPs. For more detailed clinical information, please refer to the original study.^[35]^

### 2.5 Dataset #4 for eQTL data as the replication dataset

This eQTL dataset reported by Dixon et al^[36]^ was considered as a replication dataset for subsequent integrative genomics analysis. A total of 400 participants with isolated DNA and RNA samples from Epstein-Barr virus-transformed lymphoblastoid cell lines were utilized in this study. All the subjects provided written consent, and the UK Multicentre Research Ethics Committee approved this study. For this dataset, 408,273 genotyped SNPs and 20,599 genes were incorporated to generate an eQTL resource, which is a global map of the effects of genetic variants on the expression levels of genes. It was used for the mapping of complex disease susceptibility loci.^[36]^

### 2.6 Sherlock Bayesian analysis method

Based on the assumption that altered the expression level of a specific gene might be ascribed as a risk factor of CAD, we used the Sherlock Bayesian statistical analysis (http://sherlock.ucsf.edu/) proposed by He et al^[22]^ to match the “signature” of genes from 2 chosen eQTL datasets with patterns of associations in GWAS on CAD. Extracting from the GWAS summary dataset on CAD, SNP rs IDs and *P* values were adopted as a submitted list for Sherlock analysis. The parameters α and β, which were used to specify the prior probabilities of a SNP being associated with a phenotypic trait and an expression separately, were set: α = 1.0 × 10^−3^ (*cis*) and 5.0 × 10^−5^ (*trans*), β = 1.0 × 10^−3^. The statistical inference procedures of the Sherlock algorithm are as follows: the Sherlock algorithm first utilizes the information from eQTL data to discover expression-associated SNPs (called as eSNPs). Then, the algorithm evaluates the association of eSNPs with CAD using the genome-wide association signals of SNPs from GWAS summary data. On the basis of the association significance of an eSNP with CAD, the algorithm calculates the score of the eSNP. There exist 3 scenarios:

1. If the association between eSNP and CAD is significant, the algorithm assigns a positive score to the eSNP.
2. If there is non-significant association between eSNP and CAD, the algorithm assigns a negative score to the eSNP.
3. If there is no eSNP but only the SNP significantly associated with CAD, no score is assigned.

For each gene, the Sherlock Bayesian algorithm is used to examine whether altered gene expression has any effect on the risk of CAD by using the incorporated information of the putative one or more eSNPs of this gene. Based on integrated evidence from eQTL and GWAS, the algorithm infers CAD-associated risk genes via calibrating the logarithm of Bayes factor of each gene. The logarithm of Bayes factor is a pivotal indicator for predicting promising genes associated with CAD risk. Simulation analysis was used in current Sherlock analysis to assess the significance of each gene. Simulated *P* value ≤.05 should be of significant.

### 2.7 MAGMA gene level analysis

The Multi-marker Analysis of GenoMic Annotation (MAGMA)^[37]^ was applied to conduct gene-based enrichment analysis based on the genome-wide SNPs information from GWAS summary dataset. The SNP IDs and relevant *P* values of the GWAS summary dataset were submitted as input for the MAGMA tool. For the method of MAGMA, multiple regression analysis was adopted with incorporating the information of linkage disequilibrium (LD) between SNPs within a defined genomic region to uncover multi-variant convergent effects. A SNP mapped to a gene depends on whether the location of the SNP mapped in the gene body or within a genomic region extended +/−20 kb downstream or upstream of the gene.^[38]^ The 1000 Genome European reference panel was adopted to calibrate SNP-SNP LD scores. The Human Genome Build 37 was used to indicate the location of SNPs. MAGMA's built in empirical multiple testing corrections were used to correct raw *P* values with 10,000 times of permutations.

### 2.8 Functional enrichment analysis based on pathway and GO-term resources

To identify the biological functions of these prioritized genes associated with CAD risk, we used the tool of ClueGO, a plug-in tool of Cytoscape platform,^[39]^ to perform enrichment analyses based on organized Kyoto Encyclopedia of Genes and Genomes (KEGG) pathways,^[40]^ Reactome pathways,^[41]^ Wiki pathways,^[42]^ or gene ontology (GO) terms.^[43]^ Strikingly, 4 well-applied categories of GO terms, including biological process, molecular function, cellular component, and immune system, were used in the present study. To avoid the redundancies of enriched GO terms, the function of “GO term fusion” was employed. The hypergeometric test is employed for all enrichment analyses to compute the significance.

### 2.9 Multidimensional scaling analysis for clustering enriched pathways

In order to cluster significantly enriched KEGG pathways by identified CAD-associated genes, we performed a MDS analysis. First, we organized a *pathway.txt* file that contains all the significant enriched KEGG pathways. Then, we used the Jaccard distance method to calculate pathway-pathway distance scores according to overlapped genes. By using these Jaccard distance scores, we did the MDS analysis to obtain MDS1 and MDS2 values. Final, by plotting a bubble diagram, we visualized the clusters of enriched KEGG pathways via MDS1 and MDS2 values. The most significant pathway (i.e., has the lowest *P* value) was used to represent each cluster.

### 2.10 Functional enrichment analysis based on multiple disease- and drug-based databases

The web-access tool of WebGestalt (http://www.webgestalt.org/)^[44]^ was utilized for disease- and drug-based functional enrichment analysis based on 2 commonly used databases, that is, DisGeNET^[45]^ and GLAD4U.^[46]^ Herein, we performed an overrepresentation analysis to analyze the gene list identified from Sherlock Bayesian analysis in the discovery stage while searching for significantly enriched gene sets related to diseases or targeted drugs. All functional enrichment analyses were based on the protein-coding genes. The gene size of each gene set ranged from 5 to 2000. The Benjamini–Hochberg false discovery rate was adopted for multiple corrections.

### 2.11 Protein–protein interaction (PPI)-based sub-network analysis

Accumulating evidence demonstrated that susceptible genes showed biological connections and had joint functions in the etiology of complex diseases.^[21,47,48]^ Consequently, we performed the PPI network analysis using the GeneMANIA software^[49]^ to discover the interaction patterns among the identified susceptible genes. For GeneMANIA, these identified genes were analyzed to construct a PPI-based sub-network based on published genomics and proteomics data. The network was dependent on multiple layers of evidence, that is, physical interactions, co-expression, predicted links, co-localization, pathway links, and shared protein domains.

### 2.12 Computer-based permutation analysis

In the current study, we identified a group of genes (Geneset #1: N = 634) from the Sherlock integrative analysis in the discovery stage and 2 gene sets from the Sherlock integrative analysis (Geneset #2: N = 658) in the replication stage. To determine whether these identified gene sets were highly overlapped than random events, we carried out a permutation analysis of 100,000 times.^[50]^ At the first step, we counted the number of overlapped gene (*N*_ observation_) between discovery stage (Geneset #1) and replication stage (Geneset #2). At the second step, we calculated the total number of background genes for the Sherlock analysis of Dataset #4 (*N*_ total_ = 13,152). Then, through randomly selecting the same number as the identified significant genes (Geneset #2) from background genes for 100,000 trials, we counted the number of randomly selected genes overlapped with genes of Geneset #1 (*N*_ random_). Finally, we calculated the number of times (*N*_count_) that *N*_random_*≤ N*_observation_ among 100,000 trials. The proportion of *N*_count_ divided by 100,000 was used to assess empirical *P* values, and a *P* value ≤.05 is considered to be significant.

### 2.13 Comparative analysis for MAGMA of GWAS on CAD and null trait

To further evaluate whether these CAD-risk gene were due to genetic determinants rather than random chance, we also carried out a MAGMA gene-based association analysis of GWAS on null trait (Dataset #2). There were 805 genes identified to be significantly associated with null trait (Geneset #4). Based on the MAGMA gene-based association analysis, there were 2276 genes identified to be associated with CAD (Geneset #3). For comparative analysis, we first used the different *P* values of 0.05, 0.01, and 0.001 as 3 comparative points to extract subgroup genes from Genesets #1 and #2, respectively. At each comparative point, we used these subgroup genes of Genesets #1 and #2 to overlap with Genesets #3 and #4, respectively. Then, we compared the overlapped gene rates between Sherlock analysis and MAGMA analysis on CAD with that between Sherlock analysis and MAGMA analysis on null trait at 3 different comparative points. We used the Microsoft Excel tool to visualize the results of the comparative analysis. Paired Student *t* test was used to assess the significance.

### 2.14 RNA expression dataset on CAD from gene expression omnibus (GEO) database

We downloaded an RNA expression dataset on CAD from the GEO database (Accession number GSE120774) to explore whether the expression patterns of identified genes were altered between cases and controls. With regard to this dataset,^[51]^ adult patients with preoperative coronary angiography were enrolled in the present study. Control patients underwent elective valve surgery and had no significant CAD (any single lesion > 50%) on preoperative coronary angiograms. The case patients were referred for coronary artery bypass surgery due to significant CAD. All samples provided informed consent, and the University of Massachusetts Medical Institutional Review Board (docket H-14436) approved the present study. All samples (N = 19) used in the current investigation were based on epicardial adipose tissues obtained from a site adjacent to the right coronary artery in patients with CAD (N = 9) and matched controls (N = 10). The Affymetrix Human Gene 1.0 ST microarray (Platform: GPL6244) was utilized to measure the expression levels of the target genes.

### 2.15 Statistical analysis

For the RNA expression dataset of GSE120774, we conducted a differential gene expression (DGE) analysis of identified genes between CAD patients and controls. Student *t* test was applied to assess the statistical significance, and a *P* value ≤.05 is of significance. We used the *boxplot* in R platform to visualize the differential expression level of identified genes between CAD patients and controls. To examine whether the co-expression links among these genes changed between CAD patients and matched controls based on the GSE120774 dataset, the Pearson correlation analysis was applied to calculate co-expression levels among these identified genes in CAD patients and controls separately. The *Corrplot* package from R platform was used to visualize co-expression patterns.

## 3 Results

### 3.1 Integrated genomics analysis in the discovery stage

By incorporating the large-scale GWAS summary data (N = 459,534) with 2 eQTL datasets (N = 1890), respectively, as well as multiple independent bioinformatics techniques, we attempted to identify susceptible genes with risk SNPs and abnormal expression implicated in the pathogenesis of CAD. All steps of the current investigation are shown in Figure 1. In the discovery stage, the Sherlock Bayesian analysis identified 634 genes to be significantly associated with CAD (Geneset #1, simulated *P* < .05; Supplemental Table S1); for example, *GSDML* (simulated *P* value = 1.43 × 10^−4^), *PAN3* (simulated *P* value = 1.43 × 10^−4^), *PSMC2* (simulated *P* value = 2.22 × 10^−4^), *FUBP3* (simulated *P* value = 3.80 × 10^−4^), and *SCML2* (simulated *P* value = 4.58 × 10^−4^). Among these, 15 genes were reported to be associated with CAD in the source of the GWAS Catalog database (Supplementary Table S1).

### 3.2 Functional enrichment analysis of the CAD-risk genes

**Figure 2:**
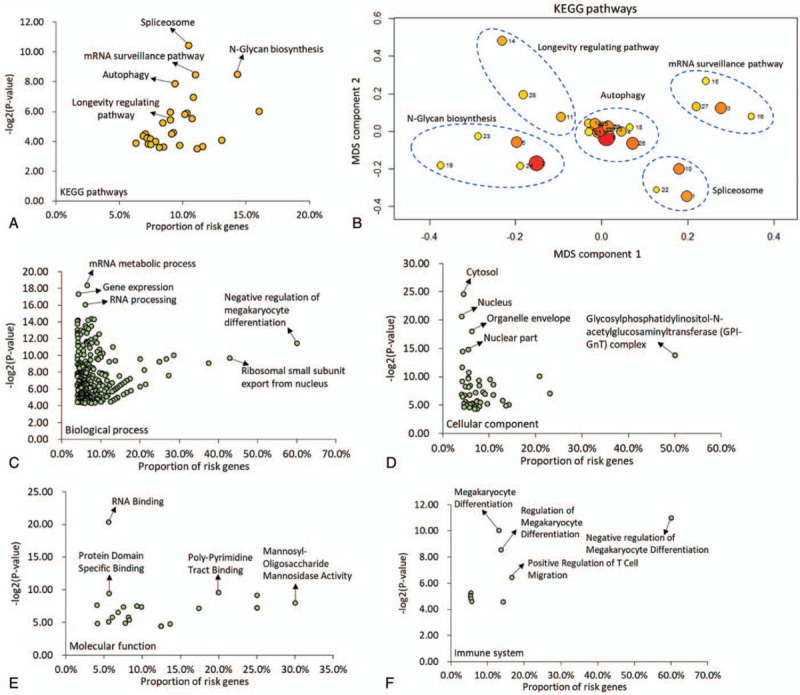
Functional enrichment analysis of CAD-risk genes. (a) KEGG pathway enrichment analysis of identified genes with 29 enriched pathways. (b) Multidimensional scaling plot of 29 KEGG enriched pathways for CAD. Circular ring size represents the number of genes in each enriched pathway. Color represents the significant level of each enriched pathway (red represents the most significant pathway with the lowest *P* value). Number in the plot represents the ID of each enriched pathway, as shown in the Supplemental Table S2. (c) GO terms of biological process enrichment analysis of identified genes. (d) GO terms of cellular component enrichment analysis of identified genes. (e) GO terms of molecular function enrichment analysis of identified genes. (f) GO terms of immune system enrichment analysis of identified genes.

By performing a pathway-based enrichment analysis, we identified significant pathways enriched by these CAD-related genes using the 3 commonly used pathway resources: KEGG, Wiki, and Reactome. For KEGG, we identified 29 significantly enriched pathways (Fig. 2a and Supplementary Table S2). Next, these pathways were clustered into 5 clusters using the MDS analysis (see Methods, Fig. 2b): autophagy (Cluster #1), longevity regulating pathway (Cluster #2), N-Glycan biosynthesis (Cluster #3), spliceosome (Cluster #4), and mRNA surveillance pathway (Cluster #5). A total of 16 and 205 significantly enriched Wiki pathways and Reactome pathways were detected, respectively (Supplementary Tables S3-S4); for example, brain-derived neurotrophic factor (BDNF) signaling pathway (*P* = 6.26 × 10^−4^), H19 action Rb-E2F1 signaling and CDK-catenin activity (*P* = 1.59 × 10^−3^), and factor I cleaved iC3b (*P* = 4.51 × 10^−5^).

With regard to the GO enrichment analysis, we identified 297, 48, 22, and 10 significantly enriched terms for biological process (Fig. 2c and Supplementary Table S5), cellular component (Fig. 2d and Supplementary Table S6), molecular function (Fig. 2e and Supplementary Table S7), and immune system (Fig. 2f and Supplementary Table S8), respectively. For example, the mRNA metabolic process (*P* = 2.94 × 10^−6^), protein domain specific binding (*P* = 1.32 × 10^−3^), and megakaryocyte differentiation (*P* = 9.66 × 10^−4^). Furthermore, we performed disease- and drug-based enrichment analysis using 2 widely-used databases of Disgenet and GLAD4U. For disease-based enrichment analysis, we identified top-ranked 20 significantly enriched gene sets relevant to the disease (Supplemental Figs. S1-S2 and Supplemental Tables S9-S10); for example, hypertensive encephalopathy (*P* = 5.56 × 10^−3^). Furthermore, we detected 10 top-enriched gene sets relevant to drug targets using the drug-focused enrichment analysis (Supplemental Fig. S3 and Supplemental Table S11).

### 3.3 Biological and technical validation of these identified risk genes

**Figure 3:**
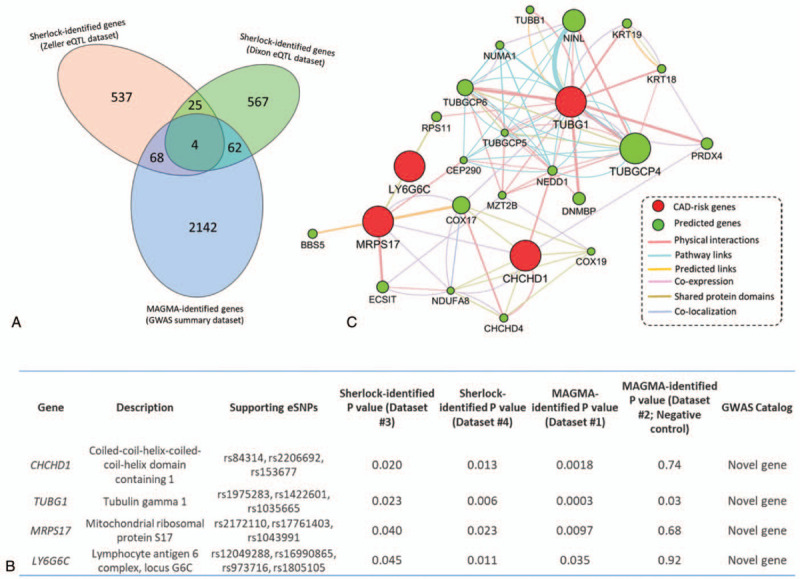
Identification of 4 susceptible genes associated with CAD risk based on independent datasets. (a) Venn diagram shows the overlapped genes among 3 gene sets identified from independent analyses: Sherlock-identified genes from Zeller eQTL data (Geneset #1), Sherlock-identified genes from Dixon eQTL data (Geneset #2), and MAGMA-identified genes from GWAS summary statistics on CAD (Geneset #3). (b) PPI network analysis of four identified CAD-risk genes. These 4 identified risk genes are marked in red, and the predicted connection genes are marked in green. The predicted attributes are based on the documented evidence of physical interaction, pathway links, predicted links, co-expression, shared protein domains, and co-localization. (c) Consistent evidence supports that these four genes indicate susceptibility to CAD.

Next, we conducted a gene-level analysis using the widely-adopted tool of MAGMA as an independent technique to replicate these identified CAD-risk genes. We found 72 MAGMA-based significant genes were overlapped with genes identified from the Sherlock analysis in the discovery stage (Fig. 3a and Supplementary Table S12). Among these, 9 genes have been documented to be significantly associated with CAD, as reported previously (Supplementary Tables S12 and S14). Subsequently, we reperformed Sherlock Bayesian analysis with the same parameters using an independent eQTL dataset as biological replication and identified 29 significantly replicated genes to be overlapped with genes identified in the discovery stage (Fig. 3a and Supplementary Tables S13 and S14). To intersect 3 identified gene sets (i.e., Genesets #1, #2, and #3), there were 4 common genes implicated in CAD risk (Fig. 3a and Fig. 3b), that is, *CHCHD1*, *TUBG1*, *MRPS17*, and *LY6G6C*.

To determine whether these 4 genes have functionally connections, we carried out a PPI network enrichment analysis using the GeneMANIA software based on public available genomics and proteomics data. Figure 3c shows that these 4 CAD-associated genes are highly connected with each other, indicating there exists a convergent effect of these genes on the etiology of CAD. Notably, each of the 4 common genes had several eSNPs, which were significantly associated with CAD and regulated the expression level of the specific gene simultaneously (Fig. 3b and Supplemental Table S15); for example, a trans-eSNP of rs84314 was significantly associated with the expression of *CHCHD1* gene (*P* = 7.09 × 10^−6^) and CAD risk (*P* = .0082).

### 3.4 Computer-based permutation analysis supporting these identified genes associated with CAD

**Figure 4:**
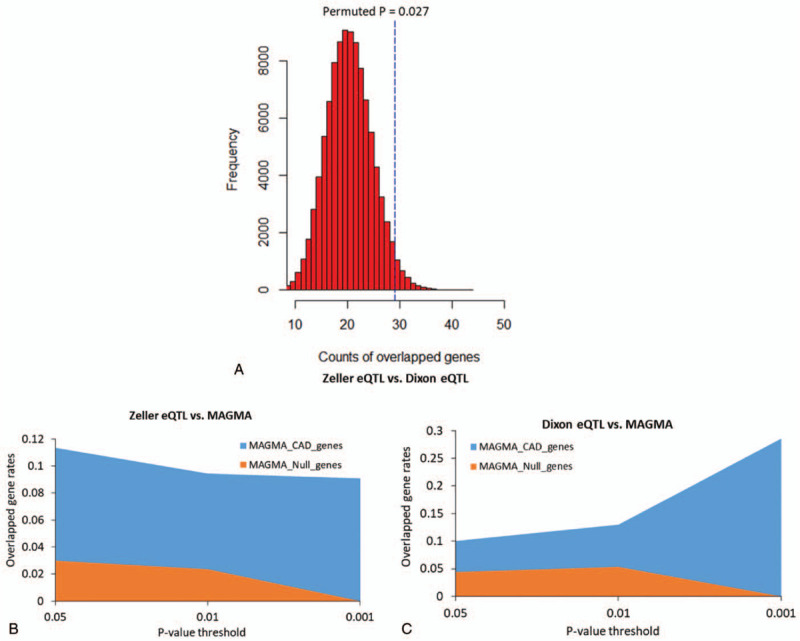
In silico permutation analysis and comparative analysis. (a) In silico permutation analysis to assess the significance of the overlapped genes between Zeller eQTL dataset (Dataset #3) and Dixon eQTL dataset (Dataset #4). (b) Sherlock-identified genes from Zeller eQTL data (Dataset #3) were remarkably overlapped with genes from MAGMA analysis on CAD-related GWAS (Dataset #1) than those from MAGMA analysis on null-related GWAS (Dataset #2). (c) Sherlock-identified genes from Dixon eQTL data (Dataset #4) were remarkably overlapped with genes from MAGMA analysis on CAD-related GWAS (Dataset #1) than those from MAGMA analysis on null-related GWAS (Dataset #2).

In order to ensure the reliability and specificity of the current analysis, we carried out a comparative analysis between real and null data. First, we performed a computer-based permutation analysis with a total of 100,000 random tests. The number of overlapped genes between Geneset #1 (discovery stage) and Geneset #2 (replication stage) were significantly higher than those overlapped between Geneset #1 and ten thousands of random selections (Fig. 4a). Furthermore, by using 3 different *P* values of .05, 0.01, and .001 as 3 comparative points, we performed a comparative analysis (see Method) to compare significant genes from Sherlock analysis and MAGMA analysis on CAD with MAGMA analysis on null trait. For all the 3 comparative points, we found that the overlapped gene rates of comparisons (Zeller eQTL vs MAGMA on CAD; Dixon eQTL vs MAGMA on CAD) were significantly higher than that from null-based comparisons (Zeller eQTL vs MAGMA on null trait; Dixon eQTL vs MAGMA on null trait) (*P* = .005, Fig. 4b-c). Together, these findings provide supportive evidence that these identified genes associated with CAD risk are likely to be attributed to genetic determinants.

### 3.5 Differential expression patterns of these 4 identified genes between CAD patients and controls

**Figure 5:**
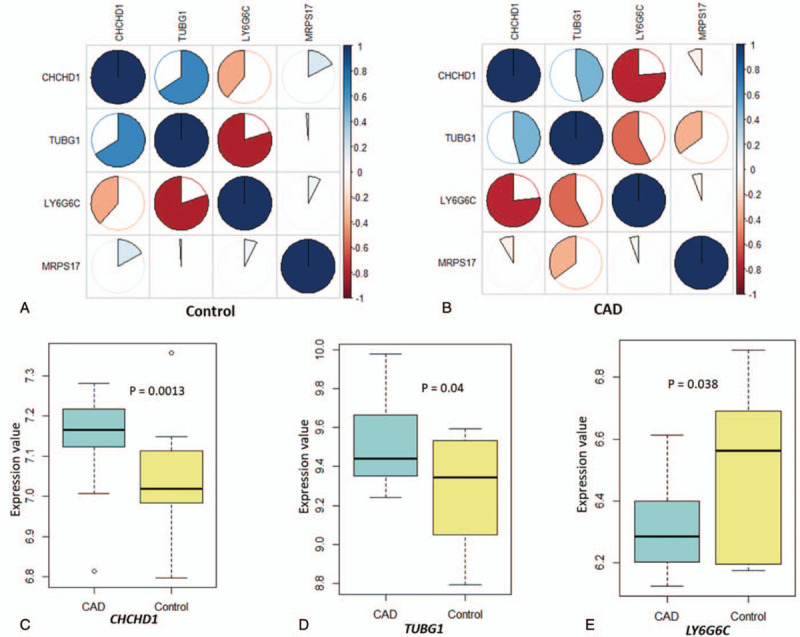
Significant differences in the expression signatures of four identified genes between CADs and controls. (a) Co-expression patterns of four identified genes in the control group based on Pearson correlation analysis. (b) Co-expression patterns of four identified genes in CAD group based on Pearson correlation analysis. (c-e) Boxplots show the significantly differential expression patterns of 3 identified genes between CAD and control groups. (c) *CHCHD1*, (d) *TUBG1*, and (e) *LY6G6C*.

By using the Pearson correlation analysis, we found remarkable altered co-expression patterns among these 4 genes between CAD patients and controls (Figure 5a and 5b, Supplementary Tables S16 and S17). For example, the negative correlation coefficient between *CHCHD1* and *LY6G6C* was −0.39 in controls, which increased to −0.76 in CAD patients. Similarly, the negative correlation coefficient between *TUBG1* and *MRPS17* was increased from −0.01 in controls to −0.35 in CADs. Conversely, the positive correlation coefficient between *CHCHD1* and *TUBG1* was decreased from 0.66 in controls to 0.46 in CADs, while that between *CHCHD1* and *MRPS17* was changed from 0.17 in controls to −0.09 in CADs.

In addition, by performing a DGE analysis, we found 3 of the 4 genes to show significantly differential expression between CAD patients and controls. Two genes of *CHCHD1* (*P* = .0013) and *TUBG1* (*P* = .04) were highly expressed in CAD patients than in controls (Figure 5c and 5d). The *LY6G6C* gene showed significantly lower expression among CAD patients as compared to that in controls (*P* = .038; Figure 5e). *MRPS17* yielded a suggestively differential expression between CAD patients and controls (*P* = .16; Supplemental Fig. S4).

## 4 Discussion

In the past decade, GWAS has been the main approach for an unbiased evaluation of the genetic determinants of CAD. Hitherto, more than 160 CAD-associated genetic loci have been identified.^[7,50]^ Nevertheless, the underlying functional effects of these identified genetic loci on CAD risk remain largely unclear. Similar to the reported SNPs associated with other complex diseases,^[17]^ a large number of identified CAD-associated SNPs were mapped into the noncoding regions, suggesting that these noncoding SNPs might affect RNA expression by *cis*- or *trans*-regulatory mechanisms in the etiology of CAD. In the present study, we conducted a systematically integrative genomics analysis based on multiomics datasets, including Sherlock Bayesian inference analysis, MAGMA gene-level analysis, MDS analysis, pathway-based enrichment analysis, in silico permutation analysis, PPI network enrichment analysis, co-expression analysis, and DGE analysis, to prioritize the genes associated with the risk of CAD.

GWAS is an effective method for the identification of risk genetic loci associated with specific complex diseases.^[52]^ Subsequently, numerous genetic variants have been reported to show significant associations with complex diseases.^[53,54]^ These GWAS-identified variants are useful for guiding researchers to perform functional genomic experiments and testing drug targets.^[55–57]^ However, the strict multiple testing correction of genome-wide SNPs at 1 GWAS was adopted, which led to a prominent reduction of the statistical power of GWAS. These SNPs, which did not gain a genome-wide significance but had vital roles in the pathogenesis of complex diseases, were largely and easily ignored under the GWAS method. Although numerous genetic loci were identified as hotspots to be associated with CAD risk, the immediate functional and biological effects of these variants are yet to be elucidated. Analyzing the correlations between genetic variants and RNA expression alterations are worthy of incorporating variation at the DNA sequence level to that at the RNA level. Thus, the Sherlock Bayesian inference analysis used in the current investigation is an effective way to reveal *cis*- and *trans*-regulatory effects of CAD-risk SNPs on RNA expression, as well as highlight susceptible genes, which cannot be discovered easily by any single GWAS.

In the present study, by incorporating a large-scale GWAS dataset with an eQTL dataset as discovery stage, we conducted the Sherlock Bayesian analysis and found 634 genes to be associated with CAD at a simulated level of significance. Some of these genes, such as *HERPUD1*,^[58]^*CCDC97,*^[58]^*MAD2L1*,^[31]^*RNF4,*^[59]^ and *ZEB2*,^[31,58,60]^ have been documented to be associated with CAD in previous GWAS studies. Furthermore, based on these significant genes, we performed a pathway enrichment analysis and identified numerous significant pathways with 5 clusters: autophagy, longevity regulating pathway, N-Glycan biosynthesis, spliceosome, and mRNA surveillance pathway, which have been implicated in the etiology of CAD,^[18,61–63]^ myocardial infarction,^[64,65]^ and heart disease.^[66,67]^ Very recently, Khera and coworkers has reviewed a group of risk genes and bilogical pathways implicated in the etiology of CAD.^[68]^ In line with Khera results, 41 of reported CAD-associated genes were significantly identified in our current investigation, including *ZEB2*, *RASD1*, *SNF8*, *PHACTR1,* and *ADAMTS7*. Additionally, these identified genes were significantly overrepresented in numerous gene sets related to drug targets, suggesting that our identified genes might be therapeutic molecular targets for the treatment of CAD.

To replicate these identified risk genes in the discovery stage, we also leveraged an independent technique replication using the MAGMA gene-level analysis. Consequently, 72 identified genes were validated. Of these, 9 have been reported to be associated with CAD in earlier GWAS studies.^[31,58,69]^ Moreover, we utilized the Sherlock Bayesian analysis with the same parameters based on an independent eQTL dataset for biological validation of the findings from the discovery stage. A total of 29 genes were replicated, and 1 gene of *HMOX1* appeared to be a potential risk determinant of CAD.^[70]^ Consistently, no matter which comparing with results from 100,000 times of random selections or MAGMA analysis of null GWAS, the overlapped rates of genes between the discovery stage and replication stage were higher. Together, through using the two-stage design genomics analysis as used in earlier reported studies,^[13,28,31,60]^ we provide multiple lines of evidence support Sherlock-identified genes have potent roles in CAD risk.

Based on the independent biological and technical replications, 4 genes, *CHCHD1*, *TUBG1*, *LY6G6C*, and *MRPS17*, were identified to be potentially implicated in the etiology of CAD. The gene of *CHCHD1*, which is a ribosomal protein, has been discovered to be indispensable for mitochondrial translation.^[71]^ Sequence variants in *CHCHD1* gene has been reported to involve in combined oxidative phosphorylation system deficiencies.^[72]^ For the *LY6G6C* gene, which belongs to a cluster of leukocyte antigen-6 (LY6) genes in the major histocompatibility complex (MHC) region. Most LY6 proteins have been documented to be attached to the cell surface by a glycosylphosphatidylinositol (GPI) anchor, which is directly involved in signal transduction.^[73]^ As for *TUBG1* gene, it encodes a member of the tubulin superfamily. Mutations in *TUBG1* cause malformations of cortical development and microcephaly.^[74]^ The protein encoded by *MRPS17* gene is moderately conserved between human mitochondrial proteins, which help in the synthesis of protein within the mitochondrion.

Furthermore, 2 hub genes of *MRPS17* and *CHCHD1* were co-expressed.^[75]^ The hub gene *MRPS17* physically interacted with *ECSIT*,^[76]^ while *TUBG1* showed physical interactions with *NINL*, *KRT19*, *RPS11*, and *NEDD1*. The hub gene of *CHCHD1* also showed physical interaction with *NEDD1*.^[77]^ Our PPI network analysis showed a large proportion of co-expression interactions among these four highlighted genes as well as other predicted genes. Consistently, we revealed remarkable changes in the co-expression interactions among these 4 genes between CAD patients and controls, indicating that these 4 genes may have joint functions in the pathogenesis of CAD. The main assumption of Sherlock Bayesian method^[22]^ is that the abnormal expression of risk genes contribute susceptibility to the diseases of interest. In agreement with this assumption, we conducted a DGE analysis and observed that 3 genes of *TUBG1*, *LY6G6C*, and *CHCHD1* were significantly expressed between CAD patients and controls. Together, we prioritized 4 genes as important candidates for CAD susceptibility.

In view of the influence of LD between SNPs, GWAS-reported genetic loci often have a number of highly LD SNPs with significant *P* values, which enhance the difficulty of searching authentic risk SNPs and relevant genes. For example, the MHC region on chromosome 6 contains many SNPs with complicated LD structures, which is hard to reveal genuine risk genes in this region by a single GWAS dataset. The method of Sherlock analysis used in our current analysis is designed to prioritize risk genes by integrating GWAS summary data with eQTL data. An eSNP for a specific risk gene should be significantly associated with CAD and expression level of this gene simultaneously. For example, the *LY6G6C* gene is mapped in a cluster of leukocyte antigen-6 (LY6) genes in the MHC region. We found 4 eSNPs of rs12049288, rs16990865, rs973716, rs1805105, which are not mapped in the MHC region, having trans-regulatory effects on *LY6G6C* expression and also associated with CAD, indicating that we could highlight CAD-risk genes which was not reported by the original GWAS by using the Sherlock integrative genomics method based on multiple omics datasets.

Some limitations of current study should be cautious. Because of there existed heterogeneities among omics datasets and bioinformatics tools used in our current study, we used different methods for multiple testing correction for each dataset and analysis. Such as, simulated *P* values were used in the Sherlock analysis, empirical *P* values were used in the in silico permutation analysis and MAGMA gene-based analysis. Moreover, the association signals of current integrative genomics analysis were based on European population. More studies based on other ethnicities are warranted. Based on the Sherlock Bayesian algorithm, these GWAS and eQTL datasets used in current integrative genomics analysis was derived from different populations. There existed heterogeneity across datasets. Further studies with GWAS and eQTL data from the same population are warranted. Although we prioritized 4 important genes associated with CAD, we did not explore the causal relationships between risk genes and CAD. Further studies are needed to uncover the molecular mechanisms of 4 genes such as *CHCHD1* and *TUBG1* for CAD risk.

## 5 Conclusions

In summary, the current investigation was based on a comprehensive in silico genomics analysis that revealed CAD-associated susceptible eSNPs, genes, and pathways. The GWAS data combined with eQTL information were used to elucidate the regulatory effects of SNPs on CAD. The topology data of protein–protein regulatory correlations 4 highlighted genes with vital roles in CAD risk. However, additional in vitro and in vivo studies are essential for the identification of the molecular functions and biological mechanisms of these prioritized genes implicated in the pathogenesis of CAD.

## Author contributions

**Conceptualization:** Yunlong Ma, Yizhou Xu.

**Data curation:** Yigang Zhong, Liuying Chen, Jingjing Li, Yinghao Yao, Qiang Liu, Kaimeng Niu, Yunlong Ma.

**Formal analysis:** Liuying Chen, Jingjing Li, Yinghao Yao, Qiang Liu, Kaimeng Niu, Yunlong Ma.

**Funding acquisition:** Yunlong Ma, Yizhou Xu.

**Methodology:** Kaimeng Niu, Yizhou Xu.

**Software:** Qiang Liu.

**Supervision:** Yigang Zhong, Yunlong Ma, Yizhou Xu.

**Visualization:** Qiang Liu, Kaimeng Niu, Yunlong Ma.

**Writing – original draft:** Yigang Zhong, Liuying Chen, Jingjing Li, Yunlong Ma, Yizhou Xu.

**Writing – review & editing:** Yigang Zhong, Yunlong Ma, Yizhou Xu.

## Supplementary Material

Supplemental Digital Content

## Supplementary Material

Supplemental Digital Content

## Supplementary Material

Supplemental Digital Content

## Supplementary Material

Supplemental Digital Content

## Supplementary Material

Supplemental Digital Content

## Supplementary Material

Supplemental Digital Content

## Supplementary Material

Supplemental Digital Content

## Supplementary Material

Supplemental Digital Content

## Supplementary Material

Supplemental Digital Content

## Supplementary Material

Supplemental Digital Content

## Supplementary Material

Supplemental Digital Content

## Supplementary Material

Supplemental Digital Content

## Supplementary Material

Supplemental Digital Content

## Supplementary Material

Supplemental Digital Content

## Supplementary Material

Supplemental Digital Content

## Supplementary Material

Supplemental Digital Content

## Supplementary Material

Supplemental Digital Content

## Supplementary Material

Supplemental Digital Content

## Supplementary Material

Supplemental Digital Content

## Supplementary Material

Supplemental Digital Content

## Supplementary Material

Supplemental Digital Content
