# Supplementary material for: Integration of summary data from GWAS and eQTL studies identified novel risk genes for coronary artery disease
Source: Medicine (Baltimore). 2021 Mar 19;100(11):e24769. doi: 10.1097/MD.0000000000024769 (PMC7982177; doi:10.1097/MD.0000000000024769)
Supplement: Supplemental Digital Content [file medi-100-e24769-s001.docx]

**Supplemental Table S1**. **Sherlock Bayesian analysis identifies 634 genes as CAD-associated risk genes in discovery samples (Zeller et al. eQTL dataset)**

| **Gene** | **LBF** | **Simulated P value** | **GWAS Catalog database** |
| --- | --- | --- | --- |
| *GSDML* | -0.04 | 1.43E-04 | Non-documented gene |
| *PAN3* | -0.04 | 1.43E-04 | Non-documented gene |
| *PSMC2* | -0.04 | 2.22E-04 | Non-documented gene |
| *FUBP3* | -0.04 | 3.80E-04 | Non-documented gene |
| *SCML2* | -0.04 | 4.58E-04 | Non-documented gene |
| *CIR* | -0.04 | 5.37E-04 | Non-documented gene |
| *PREP* | -0.04 | 6.16E-04 | Non-documented gene |
| *CCL3L3* | -0.04 | 6.95E-04 | Non-documented gene |
| *WNT2* | -0.04 | 6.96E-04 | Non-documented gene |
| *FKBP2* | -0.04 | 8.52E-04 | Non-documented gene |
| *IMPACT* | -0.04 | 8.52E-04 | Non-documented gene |
| *RECQL4* | -0.04 | 1.01E-03 | Non-documented gene |
| *TRIM39* | -0.04 | 1.09E-03 | Non-documented gene |
| *LBR* | -0.04 | 1.17E-03 | Non-documented gene |
| *GPR173* | -0.04 | 1.25E-03 | Non-documented gene |
| *SEC22A* | -0.04 | 1.32E-03 | Non-documented gene |
| *HNRPR* | -0.04 | 1.40E-03 | Non-documented gene |
| *SCYL2* | -0.04 | 1.40E-03 | Non-documented gene |
| *FBXL10* | -0.04 | 1.48E-03 | Non-documented gene |
| *TMSB10* | -0.04 | 1.64E-03 | Non-documented gene |
| *COX18* | -0.04 | 1.72E-03 | Non-documented gene |
| *ESRRAP2* | -0.04 | 1.80E-03 | Non-documented gene |
| *STAU1* | -0.04 | 1.88E-03 | Non-documented gene |
| *CCHCR1* | -0.04 | 1.95E-03 | Non-documented gene |
| *RABGGTB* | -0.04 | 2.03E-03 | Non-documented gene |
| *ZNF434* | -0.04 | 2.03E-03 | Non-documented gene |
| *STT3A* | -0.05 | 2.19E-03 | Non-documented gene |
| *KLF8* | -0.05 | 2.27E-03 | Non-documented gene |
| *DNAJC10* | -0.05 | 2.35E-03 | Non-documented gene |
| *ADAM17* | -0.05 | 2.43E-03 | Non-documented gene |
| *SLC25A20* | -0.05 | 2.51E-03 | Non-documented gene |
| *ATMIN* | -0.05 | 2.59E-03 | Non-documented gene |
| *DYNLT3* | -0.05 | 2.66E-03 | Non-documented gene |
| *CCT4* | -0.05 | 2.74E-03 | Non-documented gene |
| *IRS2* | -0.05 | 2.74E-03 | Non-documented gene |
| *PIGC* | -0.05 | 2.90E-03 | Non-documented gene |
| *DDI2* | -0.05 | 2.90E-03 | Non-documented gene |
| *GPC4* | -0.05 | 2.98E-03 | Non-documented gene |
| *BCL2A1* | -0.05 | 3.14E-03 | Non-documented gene |
| *ATP6V0E1* | -0.05 | 3.22E-03 | Non-documented gene |
| *FREM2* | -0.05 | 3.29E-03 | Non-documented gene |
| *HNRNPU* | -0.05 | 3.37E-03 | Non-documented gene |
| *GPR26* | -0.05 | 3.37E-03 | Non-documented gene |
| *KEAP1* | -0.05 | 3.45E-03 | Non-documented gene |
| *KARS* | -0.05 | 3.53E-03 | Non-documented gene |
| *PDPK1* | -0.05 | 3.61E-03 | Non-documented gene |
| *MAOA* | -0.05 | 3.69E-03 | Non-documented gene |
| *PTPN2* | -0.05 | 3.85E-03 | Non-documented gene |
| *ZNF701* | -0.05 | 3.85E-03 | Non-documented gene |
| *NFIL3* | -0.05 | 3.92E-03 | Non-documented gene |
| *SAC* | -0.05 | 4.00E-03 | Non-documented gene |
| *PRY2* | -0.05 | 4.08E-03 | Non-documented gene |
| *CCDC128* | -0.05 | 4.16E-03 | Non-documented gene |
| *VIT* | -0.05 | 4.32E-03 | Non-documented gene |
| *LHFPL4* | -0.05 | 4.32E-03 | Non-documented gene |
| *GABPA* | -0.05 | 4.48E-03 | Non-documented gene |
| *GJB3* | -0.05 | 4.48E-03 | Non-documented gene |
| *CHM* | -0.05 | 4.63E-03 | Non-documented gene |
| *CHCHD3* | -0.05 | 4.63E-03 | Non-documented gene |
| *SHC3* | -0.05 | 4.79E-03 | Non-documented gene |
| *CCDC76* | -0.05 | 4.79E-03 | Non-documented gene |
| *HERPUD1* | -0.05 | 4.95E-03 | Reported gene |
| *GABARAP* | -0.05 | 4.95E-03 | Non-documented gene |
| *EFR3A* | -0.05 | 5.03E-03 | Non-documented gene |
| *HSFX1* | -0.05 | 5.11E-03 | Non-documented gene |
| *STRA13* | -0.05 | 5.18E-03 | Non-documented gene |
| *ATF7IP2* | -0.05 | 5.32E-03 | Non-documented gene |
| *PRMT1* | -0.05 | 5.34E-03 | Non-documented gene |
| *TMED2* | -0.05 | 5.50E-03 | Non-documented gene |
| *MLL5* | -0.05 | 5.58E-03 | Non-documented gene |
| *CCDC97* | -0.05 | 5.58E-03 | Reported gene |
| *FOXP1* | -0.05 | 5.74E-03 | Non-documented gene |
| *UQCRFS1* | -0.05 | 5.82E-03 | Non-documented gene |
| *DHFRL1* | -0.05 | 5.90E-03 | Non-documented gene |
| *MATR3* | -0.05 | 5.98E-03 | Non-documented gene |
| *ZNF655* | -0.05 | 6.05E-03 | Non-documented gene |
| *CCDC89* | -0.05 | 6.05E-03 | Non-documented gene |
| *NCAPG* | -0.05 | 6.21E-03 | Non-documented gene |
| *TALDO1* | -0.05 | 6.21E-03 | Non-documented gene |
| *DNAJC1* | -0.06 | 6.29E-03 | Non-documented gene |
| *ATG3* | -0.06 | 6.37E-03 | Non-documented gene |
| *DAPK2* | -0.06 | 6.45E-03 | Non-documented gene |
| *UBB* | -0.06 | 6.53E-03 | Non-documented gene |
| *GZMK* | -0.06 | 6.61E-03 | Non-documented gene |
| *GRSF1* | -0.06 | 6.75E-03 | Non-documented gene |
| *ACTR6* | -0.06 | 6.76E-03 | Non-documented gene |
| *ASTN2* | -0.06 | 6.84E-03 | Non-documented gene |
| *NUDT21* | -0.06 | 6.92E-03 | Non-documented gene |
| *TCHP* | -0.06 | 7.08E-03 | Non-documented gene |
| *DPM2* | -0.06 | 7.08E-03 | Non-documented gene |
| *OXGR1* | -0.06 | 7.24E-03 | Non-documented gene |
| *CFI* | -0.06 | 7.24E-03 | Non-documented gene |
| *GNG3* | -0.06 | 7.39E-03 | Non-documented gene |
| *TESK1* | -0.06 | 7.39E-03 | Non-documented gene |
| *SCLY* | -0.06 | 7.47E-03 | Non-documented gene |
| *OGFR* | -0.06 | 7.63E-03 | Non-documented gene |
| *RBM42* | -0.06 | 7.71E-03 | Non-documented gene |
| *FAM113B* | -0.06 | 7.71E-03 | Non-documented gene |
| *NDUFA4* | -0.06 | 7.79E-03 | Non-documented gene |
| *CABIN1* | -0.06 | 7.87E-03 | Reported gene |
| *ONECUT2* | -0.06 | 7.94E-03 | Non-documented gene |
| *SMARCAD1* | -0.06 | 8.02E-03 | Non-documented gene |
| *PLEKHM1* | -0.06 | 8.10E-03 | Non-documented gene |
| *GOLGA2* | -0.06 | 8.18E-03 | Non-documented gene |
| *GJC3* | -0.06 | 8.26E-03 | Non-documented gene |
| *JOSD1* | -0.06 | 8.34E-03 | Non-documented gene |
| *DGAT1* | -0.06 | 8.42E-03 | Non-documented gene |
| *DPPA2* | -0.06 | 8.57E-03 | Non-documented gene |
| *COPG* | -0.06 | 8.63E-03 | Non-documented gene |
| *ATP6V1B1* | -0.06 | 8.65E-03 | Non-documented gene |
| *PHF20L1* | -0.06 | 8.73E-03 | Non-documented gene |
| *MAGEH1* | -0.06 | 8.81E-03 | Non-documented gene |
| *SMARCA5* | -0.06 | 8.89E-03 | Non-documented gene |
| *PDE2A* | -0.06 | 8.97E-03 | Non-documented gene |
| *OR2AG2* | -0.06 | 9.05E-03 | Non-documented gene |
| *TBC1D14* | -0.06 | 9.13E-03 | Non-documented gene |
| *SNX14* | -0.06 | 9.21E-03 | Non-documented gene |
| *RAG1AP1* | -0.06 | 9.29E-03 | Non-documented gene |
| *CHKA* | -0.06 | 9.36E-03 | Non-documented gene |
| *FAM122B* | -0.06 | 9.44E-03 | Non-documented gene |
| *SCPEP1* | -0.06 | 9.53E-03 | Non-documented gene |
| *NRAS* | -0.06 | 9.68E-03 | Non-documented gene |
| *FAM152B* | -0.06 | 9.68E-03 | Non-documented gene |
| *TMEM135* | -0.06 | 9.84E-03 | Non-documented gene |
| *CNIH2* | -0.06 | 9.92E-03 | Non-documented gene |
| *CAPN13* | -0.06 | 9.92E-03 | Non-documented gene |
| *SNTB1* | -0.06 | 1.00E-02 | Non-documented gene |
| *ZNF446* | -0.06 | 1.01E-02 | Non-documented gene |
| *STXBP3* | -0.06 | 1.02E-02 | Non-documented gene |
| *PRPS1* | -0.06 | 1.02E-02 | Non-documented gene |
| *KCNIP2* | -0.06 | 1.04E-02 | Non-documented gene |
| *CENTA2* | -0.06 | 1.05E-02 | Non-documented gene |
| *PTMS* | -0.06 | 1.05E-02 | Non-documented gene |
| *MTDH* | -0.06 | 1.05E-02 | Non-documented gene |
| *PRPSAP1* | -0.07 | 1.06E-02 | Non-documented gene |
| *HOXB1* | -0.07 | 1.08E-02 | Non-documented gene |
| *DHX29* | -0.07 | 1.08E-02 | Non-documented gene |
| *UNC5CL* | -0.07 | 1.09E-02 | Non-documented gene |
| *SLC25A42* | -0.07 | 1.09E-02 | Non-documented gene |
| *EWSR1* | -0.07 | 1.10E-02 | Non-documented gene |
| *HIST1H3J* | -0.07 | 1.10E-02 | Non-documented gene |
| *EIF4EBP1* | -0.07 | 1.12E-02 | Non-documented gene |
| *PIN1* | -0.07 | 1.13E-02 | Non-documented gene |
| *LOXL3* | -0.07 | 1.13E-02 | Non-documented gene |
| *ACY1* | -0.07 | 1.14E-02 | Non-documented gene |
| *MSI1* | -0.07 | 1.16E-02 | Non-documented gene |
| *RPUSD1* | -0.07 | 1.16E-02 | Non-documented gene |
| *FTHL11* | -0.07 | 1.16E-02 | Non-documented gene |
| *MTCP1* | -0.07 | 1.17E-02 | Non-documented gene |
| *ADAM10* | -0.07 | 1.18E-02 | Non-documented gene |
| *GOLT1B* | -0.07 | 1.19E-02 | Non-documented gene |
| *TMEM49* | -0.07 | 1.20E-02 | Non-documented gene |
| *CCL3L1* | -0.07 | 1.20E-02 | Non-documented gene |
| *USP3* | -0.07 | 1.21E-02 | Non-documented gene |
| *SFRS2B* | -0.07 | 1.23E-02 | Non-documented gene |
| *C3* | -0.07 | 1.24E-02 | Non-documented gene |
| *FEN1* | -0.07 | 1.24E-02 | Non-documented gene |
| *JARID1D* | -0.07 | 1.25E-02 | Non-documented gene |
| *B3GAT3* | -0.07 | 1.25E-02 | Non-documented gene |
| *LCA5* | -0.07 | 1.27E-02 | Non-documented gene |
| *FDX1L* | -0.07 | 1.27E-02 | Non-documented gene |
| *HADHB* | -0.07 | 1.28E-02 | Non-documented gene |
| *PPTC7* | -0.07 | 1.28E-02 | Non-documented gene |
| *ENO1* | -0.07 | 1.29E-02 | Non-documented gene |
| *PTP4A1* | -0.07 | 1.31E-02 | Non-documented gene |
| *U2AF1L2* | -0.07 | 1.31E-02 | Non-documented gene |
| *SEC61B* | -0.07 | 1.31E-02 | Non-documented gene |
| *SCYL3* | -0.07 | 1.32E-02 | Non-documented gene |
| *MRPL22* | -0.07 | 1.33E-02 | Non-documented gene |
| *POGK* | -0.07 | 1.34E-02 | Non-documented gene |
| *MAZ* | -0.07 | 1.35E-02 | Non-documented gene |
| *ZNF98* | -0.07 | 1.35E-02 | Non-documented gene |
| *GAPDH* | -0.07 | 1.36E-02 | Non-documented gene |
| *ANXA9* | -0.07 | 1.38E-02 | Non-documented gene |
| *PMVK* | -0.07 | 1.38E-02 | Non-documented gene |
| *NDUFV2* | -0.07 | 1.39E-02 | Non-documented gene |
| *SCUBE2* | -0.07 | 1.40E-02 | Non-documented gene |
| *ZNF17* | -0.08 | 1.40E-02 | Non-documented gene |
| *PBX2* | -0.08 | 1.41E-02 | Non-documented gene |
| *ADIG* | -0.08 | 1.42E-02 | Non-documented gene |
| *CRX* | -0.08 | 1.43E-02 | Non-documented gene |
| *ZNF384* | -0.08 | 1.43E-02 | Non-documented gene |
| *RBM11* | -0.08 | 1.45E-02 | Non-documented gene |
| *PDE6D* | -0.08 | 1.45E-02 | Non-documented gene |
| *PTP4A2* | -0.08 | 1.46E-02 | Non-documented gene |
| *DKFZP667M2411* | -0.08 | 1.46E-02 | Non-documented gene |
| *CCDC106* | -0.08 | 1.47E-02 | Non-documented gene |
| *SEMA3F* | -0.08 | 1.48E-02 | Non-documented gene |
| *PCM1* | -0.08 | 1.49E-02 | Non-documented gene |
| *DENND1C* | -0.08 | 1.50E-02 | Non-documented gene |
| *SLC19A1* | -0.08 | 1.50E-02 | Non-documented gene |
| *FNBP4* | -0.08 | 1.51E-02 | Non-documented gene |
| *SPCS2* | -0.08 | 1.52E-02 | Non-documented gene |
| *BOLA2* | -0.08 | 1.54E-02 | Non-documented gene |
| *REXO1* | -0.08 | 1.54E-02 | Non-documented gene |
| *CCNT1* | -0.08 | 1.55E-02 | Non-documented gene |
| *FBXW2* | -0.08 | 1.55E-02 | Non-documented gene |
| *FMNL3* | -0.08 | 1.57E-02 | Non-documented gene |
| *PPP4R1* | -0.08 | 1.57E-02 | Non-documented gene |
| *EP400* | -0.08 | 1.57E-02 | Non-documented gene |
| *FDXR* | -0.08 | 1.58E-02 | Non-documented gene |
| *BRD9* | -0.08 | 1.59E-02 | Non-documented gene |
| *CEPT1* | -0.08 | 1.60E-02 | Non-documented gene |
| *RGS13* | -0.08 | 1.61E-02 | Non-documented gene |
| *KDR* | -0.08 | 1.62E-02 | Non-documented gene |
| *RHOT1* | -0.08 | 1.63E-02 | Non-documented gene |
| *DRG2* | -0.08 | 1.63E-02 | Non-documented gene |
| *DAZAP2* | -0.08 | 1.65E-02 | Non-documented gene |
| *MARCKSL1* | -0.09 | 1.65E-02 | Non-documented gene |
| *EIF2C3* | -0.09 | 1.66E-02 | Non-documented gene |
| *GPR82* | -0.09 | 1.66E-02 | Non-documented gene |
| *XPO1* | -0.09 | 1.67E-02 | Non-documented gene |
| *SBNO2* | -0.09 | 1.69E-02 | Non-documented gene |
| *KPNA3* | -0.09 | 1.69E-02 | Non-documented gene |
| *TMPRSS7* | -0.09 | 1.70E-02 | Non-documented gene |
| *MAD2L1* | -0.09 | 1.70E-02 | Reported gene |
| *CD70* | -0.09 | 1.71E-02 | Non-documented gene |
| *ADAT3* | -0.09 | 1.72E-02 | Non-documented gene |
| *HPCAL4* | -0.09 | 1.73E-02 | Non-documented gene |
| *TSSK6* | -0.09 | 1.74E-02 | Non-documented gene |
| *TMEM126A* | -0.09 | 1.74E-02 | Non-documented gene |
| *CCDC8* | -0.09 | 1.75E-02 | Non-documented gene |
| *APOOL* | -0.09 | 1.75E-02 | Non-documented gene |
| *WDR68* | -0.09 | 1.76E-02 | Non-documented gene |
| *MS4A7* | -0.09 | 1.76E-02 | Non-documented gene |
| *RIT1* | -0.09 | 1.78E-02 | Non-documented gene |
| *LRRC40* | -0.09 | 1.80E-02 | Non-documented gene |
| *ASXL1* | -0.09 | 1.80E-02 | Non-documented gene |
| *PTPN11* | -0.09 | 1.80E-02 | Non-documented gene |
| *ATP6V0A2* | -0.09 | 1.81E-02 | Non-documented gene |
| *WHSC1L1* | -0.09 | 1.82E-02 | Non-documented gene |
| *CDC42BPA* | -0.09 | 1.83E-02 | Non-documented gene |
| *CKAP4* | -0.09 | 1.84E-02 | Non-documented gene |
| *MYEF2* | -0.09 | 1.85E-02 | Non-documented gene |
| *SNORA64* | -0.09 | 1.85E-02 | Non-documented gene |
| *RPS6KA5* | -0.09 | 1.87E-02 | Non-documented gene |
| *HMOX1* | -0.09 | 1.87E-02 | Reported gene |
| *SLC4A5* | -0.09 | 1.87E-02 | Non-documented gene |
| *TMEM170* | -0.09 | 1.88E-02 | Non-documented gene |
| *IL17RA* | -0.09 | 1.90E-02 | Non-documented gene |
| *RAB5A* | -0.09 | 1.90E-02 | Non-documented gene |
| *REN* | -0.09 | 1.91E-02 | Non-documented gene |
| *MPND* | -0.09 | 1.91E-02 | Non-documented gene |
| *CDK5* | -0.09 | 1.92E-02 | Non-documented gene |
| *INTS9* | -0.09 | 1.93E-02 | Non-documented gene |
| *PPARBP* | -0.09 | 1.94E-02 | Non-documented gene |
| *RB1* | -0.09 | 1.95E-02 | Non-documented gene |
| *GPR101* | -0.09 | 1.96E-02 | Non-documented gene |
| *ARS2* | -0.10 | 1.96E-02 | Non-documented gene |
| *ACSL4* | -0.10 | 1.97E-02 | Non-documented gene |
| *ZNF789* | -0.10 | 1.98E-02 | Non-documented gene |
| *SBF2* | -0.10 | 1.99E-02 | Non-documented gene |
| *ZNF282* | -0.10 | 1.99E-02 | Non-documented gene |
| *PCID2* | -0.10 | 2.00E-02 | Non-documented gene |
| *CYP2R1* | -0.10 | 2.01E-02 | Non-documented gene |
| *SHH* | -0.10 | 2.02E-02 | Non-documented gene |
| *DVL2* | -0.10 | 2.03E-02 | Non-documented gene |
| *SLC27A1* | -0.10 | 2.03E-02 | Non-documented gene |
| *CHCHD1* | -0.10 | 2.04E-02 | Non-documented gene |
| *HIST2H2AA3* | -0.10 | 2.05E-02 | Non-documented gene |
| *KATNB1* | -0.10 | 2.06E-02 | Non-documented gene |
| *ARMCX6* | -0.10 | 2.06E-02 | Non-documented gene |
| *ZNF7* | -0.10 | 2.08E-02 | Non-documented gene |
| *CHD4* | -0.10 | 2.09E-02 | Non-documented gene |
| *ATP1B2* | -0.10 | 2.09E-02 | Non-documented gene |
| *FAM126B* | -0.10 | 2.10E-02 | Non-documented gene |
| *MAP3K1* | -0.10 | 2.10E-02 | Reported gene |
| *HNRNPUL2* | -0.10 | 2.11E-02 | Non-documented gene |
| *TNRC6B* | -0.10 | 2.13E-02 | Non-documented gene |
| *CD97* | -0.10 | 2.13E-02 | Non-documented gene |
| *AVPR1B* | -0.10 | 2.14E-02 | Non-documented gene |
| *SPC24* | -0.10 | 2.14E-02 | Non-documented gene |
| *SRP14* | -0.10 | 2.15E-02 | Non-documented gene |
| *MOCS1* | -0.10 | 2.16E-02 | Non-documented gene |
| *HHLA3* | -0.10 | 2.17E-02 | Non-documented gene |
| *RB1CC1* | -0.10 | 2.17E-02 | Non-documented gene |
| *SF3A3* | -0.10 | 2.18E-02 | Reported gene |
| *ETF1* | -0.10 | 2.20E-02 | Non-documented gene |
| *KLHDC8A* | -0.10 | 2.21E-02 | Non-documented gene |
| *CIDECP* | -0.10 | 2.21E-02 | Non-documented gene |
| *ELMOD2* | -0.10 | 2.21E-02 | Non-documented gene |
| *SULT1A3* | -0.10 | 2.22E-02 | Non-documented gene |
| *CCDC63* | -0.10 | 2.23E-02 | Non-documented gene |
| *TCERG1* | -0.10 | 2.24E-02 | Non-documented gene |
| *GALNT6* | -0.10 | 2.25E-02 | Non-documented gene |
| *SQSTM1* | -0.10 | 2.25E-02 | Non-documented gene |
| *PHB* | -0.10 | 2.27E-02 | Reported gene |
| *PWWP2* | -0.10 | 2.28E-02 | Non-documented gene |
| *TXNDC15* | -0.10 | 2.28E-02 | Non-documented gene |
| *TUBG1* | -0.10 | 2.28E-02 | Non-documented gene |
| *SCAMP3* | -0.10 | 2.30E-02 | Non-documented gene |
| *B4GALT3* | -0.10 | 2.30E-02 | Non-documented gene |
| *EMG1* | -0.10 | 2.31E-02 | Non-documented gene |
| *AIG1* | -0.10 | 2.32E-02 | Non-documented gene |
| *GPAM* | -0.10 | 2.32E-02 | Non-documented gene |
| *NBPF10* | -0.10 | 2.33E-02 | Non-documented gene |
| *CT47.7* | -0.10 | 2.34E-02 | Non-documented gene |
| *SLC25A3* | -0.10 | 2.35E-02 | Non-documented gene |
| *PECAM1* | -0.10 | 2.36E-02 | Reported gene |
| *CSGLCA-T* | -0.10 | 2.36E-02 | Non-documented gene |
| *UBE2W* | -0.11 | 2.37E-02 | Non-documented gene |
| *SYF2* | -0.11 | 2.38E-02 | Non-documented gene |
| *RIC8A* | -0.11 | 2.39E-02 | Non-documented gene |
| *UBQLN2* | -0.11 | 2.39E-02 | Non-documented gene |
| *RAB4B* | -0.11 | 2.40E-02 | Non-documented gene |
| *CHD7* | -0.11 | 2.41E-02 | Non-documented gene |
| *LRDD* | -0.11 | 2.42E-02 | Non-documented gene |
| *DYNC1LI2* | -0.11 | 2.42E-02 | Non-documented gene |
| *POU3F4* | -0.11 | 2.43E-02 | Non-documented gene |
| *PTPRH* | -0.11 | 2.44E-02 | Non-documented gene |
| *LDHA* | -0.11 | 2.44E-02 | Non-documented gene |
| *SCHIP1* | -0.11 | 2.46E-02 | Non-documented gene |
| *PMPCB* | -0.11 | 2.46E-02 | Non-documented gene |
| *P4HA1* | -0.11 | 2.46E-02 | Non-documented gene |
| *PF4* | -0.11 | 2.47E-02 | Non-documented gene |
| *DLGAP4* | -0.11 | 2.49E-02 | Non-documented gene |
| *PLEKHO2* | -0.11 | 2.50E-02 | Non-documented gene |
| *ACTR10* | -0.11 | 2.51E-02 | Non-documented gene |
| *JARID2* | -0.11 | 2.52E-02 | Non-documented gene |
| *EFHA1* | -0.11 | 2.53E-02 | Non-documented gene |
| *REEP4* | -0.11 | 2.54E-02 | Non-documented gene |
| *FCGR2C* | -0.11 | 2.54E-02 | Non-documented gene |
| *MRGPRF* | -0.11 | 2.54E-02 | Non-documented gene |
| *LHX6* | -0.11 | 2.55E-02 | Non-documented gene |
| *OR8G2* | -0.11 | 2.56E-02 | Non-documented gene |
| *VPS13B* | -0.11 | 2.58E-02 | Non-documented gene |
| *HINT3* | -0.11 | 2.58E-02 | Non-documented gene |
| *INSL3* | -0.11 | 2.58E-02 | Non-documented gene |
| *CCDC132* | -0.11 | 2.58E-02 | Non-documented gene |
| *NFE2* | -0.11 | 2.59E-02 | Non-documented gene |
| *PDE7B* | -0.11 | 2.61E-02 | Non-documented gene |
| *CYBA* | -0.11 | 2.62E-02 | Non-documented gene |
| *SFRS1* | -0.11 | 2.62E-02 | Non-documented gene |
| *RNF38* | -0.11 | 2.63E-02 | Non-documented gene |
| *TUT1* | -0.11 | 2.63E-02 | Non-documented gene |
| *TTC31* | -0.11 | 2.65E-02 | Non-documented gene |
| *CASZ1* | -0.11 | 2.65E-02 | Non-documented gene |
| *PIK3CA* | -0.11 | 2.65E-02 | Non-documented gene |
| *KRTAP10-8* | -0.11 | 2.66E-02 | Non-documented gene |
| *PITPNM2* | -0.11 | 2.68E-02 | Non-documented gene |
| *AK3* | -0.11 | 2.69E-02 | Non-documented gene |
| *C5AR1* | -0.11 | 2.70E-02 | Non-documented gene |
| *PTS* | -0.11 | 2.70E-02 | Non-documented gene |
| *ZNF3* | -0.11 | 2.72E-02 | Non-documented gene |
| *CCDC126* | -0.11 | 2.72E-02 | Non-documented gene |
| *ARHGEF11* | -0.11 | 2.73E-02 | Non-documented gene |
| *HLA-DRA* | -0.11 | 2.73E-02 | Non-documented gene |
| *LPIN3* | -0.11 | 2.74E-02 | Non-documented gene |
| *IGF2R* | -0.11 | 2.75E-02 | Reported gene |
| *LONRF1* | -0.11 | 2.76E-02 | Non-documented gene |
| *GPR97* | -0.11 | 2.77E-02 | Non-documented gene |
| *ARMCX5* | -0.11 | 2.77E-02 | Non-documented gene |
| *SSR4* | -0.11 | 2.78E-02 | Non-documented gene |
| *FBXO4* | -0.11 | 2.79E-02 | Non-documented gene |
| *SF3B2* | -0.12 | 2.80E-02 | Non-documented gene |
| *PPIE* | -0.12 | 2.81E-02 | Non-documented gene |
| *ZP1* | -0.12 | 2.81E-02 | Non-documented gene |
| *ACMSD* | -0.12 | 2.82E-02 | Non-documented gene |
| *CFL1* | -0.12 | 2.83E-02 | Non-documented gene |
| *RPL22* | -0.12 | 2.84E-02 | Non-documented gene |
| *APOBEC3B* | -0.12 | 2.84E-02 | Non-documented gene |
| *DIAPH1* | -0.12 | 2.85E-02 | Non-documented gene |
| *PCNP* | -0.12 | 2.86E-02 | Non-documented gene |
| *UFC1* | -0.12 | 2.87E-02 | Non-documented gene |
| *RPS19* | -0.12 | 2.87E-02 | Non-documented gene |
| *KIF3B* | -0.12 | 2.88E-02 | Non-documented gene |
| *PABPC4* | -0.12 | 2.89E-02 | Non-documented gene |
| *MDC1* | -0.12 | 2.90E-02 | Non-documented gene |
| *PPP1R2* | -0.12 | 2.90E-02 | Non-documented gene |
| *THAP7* | -0.12 | 2.91E-02 | Non-documented gene |
| *DPH4* | -0.12 | 2.92E-02 | Non-documented gene |
| *CBX5* | -0.12 | 2.93E-02 | Non-documented gene |
| *ARRB2* | -0.12 | 2.95E-02 | Non-documented gene |
| *MAN2A2* | -0.12 | 2.95E-02 | Non-documented gene |
| *USP13* | -0.12 | 2.95E-02 | Non-documented gene |
| *CMTM7* | -0.12 | 2.96E-02 | Non-documented gene |
| *DOLPP1* | -0.12 | 2.97E-02 | Non-documented gene |
| *NUP155* | -0.12 | 2.99E-02 | Non-documented gene |
| *TNFRSF14* | -0.12 | 2.99E-02 | Non-documented gene |
| *MIB2* | -0.12 | 2.99E-02 | Non-documented gene |
| *SIPA1* | -0.12 | 3.00E-02 | Non-documented gene |
| *MTM1* | -0.12 | 3.01E-02 | Non-documented gene |
| *PI4K2B* | -0.12 | 3.02E-02 | Non-documented gene |
| *OBFC2B* | -0.12 | 3.02E-02 | Non-documented gene |
| *FATE1* | -0.12 | 3.03E-02 | Non-documented gene |
| *SEMG1* | -0.12 | 3.05E-02 | Non-documented gene |
| *SEMA6B* | -0.12 | 3.05E-02 | Non-documented gene |
| *PYCARD* | -0.12 | 3.06E-02 | Non-documented gene |
| *TULP3* | -0.12 | 3.06E-02 | Non-documented gene |
| *SLC35B2* | -0.12 | 3.07E-02 | Non-documented gene |
| *SH3BGRL* | -0.12 | 3.08E-02 | Non-documented gene |
| *P2RY13* | -0.12 | 3.09E-02 | Non-documented gene |
| *CFB* | -0.12 | 3.10E-02 | Reported gene |
| *GNAI2* | -0.12 | 3.10E-02 | Non-documented gene |
| *RPL7* | -0.12 | 3.12E-02 | Non-documented gene |
| *UCHL5IP* | -0.12 | 3.12E-02 | Non-documented gene |
| *PPP2R2D* | -0.12 | 3.13E-02 | Non-documented gene |
| *CHIC2* | -0.12 | 3.13E-02 | Non-documented gene |
| *LIMK2* | -0.12 | 3.14E-02 | Non-documented gene |
| *PIM2* | -0.12 | 3.15E-02 | Non-documented gene |
| *HIST1H2AG* | -0.12 | 3.16E-02 | Non-documented gene |
| *ZNF654* | -0.12 | 3.17E-02 | Non-documented gene |
| *TCOF1* | -0.12 | 3.17E-02 | Non-documented gene |
| *ELAC1* | -0.12 | 3.19E-02 | Non-documented gene |
| *RN7SL1* | -0.12 | 3.20E-02 | Non-documented gene |
| *GFI1* | -0.12 | 3.20E-02 | Non-documented gene |
| *MGA* | -0.12 | 3.21E-02 | Non-documented gene |
| *LRRC58* | -0.12 | 3.21E-02 | Non-documented gene |
| *EIF3M* | -0.12 | 3.22E-02 | Non-documented gene |
| *SLC30A9* | -0.12 | 3.23E-02 | Non-documented gene |
| *RHOBTB1* | -0.12 | 3.25E-02 | Non-documented gene |
| *UCK2* | -0.12 | 3.25E-02 | Non-documented gene |
| *PAIP2* | -0.12 | 3.25E-02 | Non-documented gene |
| *MTPN* | -0.12 | 3.27E-02 | Non-documented gene |
| *ABR* | -0.12 | 3.27E-02 | Non-documented gene |
| *KPNB1* | -0.12 | 3.28E-02 | Non-documented gene |
| *OCLM* | -0.12 | 3.28E-02 | Non-documented gene |
| *PPP2R1A* | -0.12 | 3.29E-02 | Non-documented gene |
| *TRY6* | -0.12 | 3.31E-02 | Non-documented gene |
| *PIGH* | -0.13 | 3.32E-02 | Non-documented gene |
| *PPP1CA* | -0.13 | 3.32E-02 | Non-documented gene |
| *RNF181* | -0.13 | 3.33E-02 | Non-documented gene |
| *TUBGCP6* | -0.13 | 3.33E-02 | Non-documented gene |
| *TIMP1* | -0.13 | 3.34E-02 | Non-documented gene |
| *MATN2* | -0.13 | 3.35E-02 | Non-documented gene |
| *RNF4* | -0.13 | 3.36E-02 | Reported gene |
| *TFE3* | -0.13 | 3.36E-02 | Non-documented gene |
| *FARSLB* | -0.13 | 3.37E-02 | Non-documented gene |
| *MID2* | -0.13 | 3.38E-02 | Non-documented gene |
| *SAT1* | -0.13 | 3.39E-02 | Non-documented gene |
| *MRPS18A* | -0.13 | 3.39E-02 | Non-documented gene |
| *SNORD31* | -0.13 | 3.41E-02 | Non-documented gene |
| *NFKBIB* | -0.13 | 3.41E-02 | Non-documented gene |
| *EHMT1* | -0.13 | 3.42E-02 | Non-documented gene |
| *CLUL1* | -0.13 | 3.43E-02 | Non-documented gene |
| *SESN1* | -0.13 | 3.43E-02 | Non-documented gene |
| *TXNDC2* | -0.13 | 3.45E-02 | Non-documented gene |
| *PEX5* | -0.13 | 3.45E-02 | Non-documented gene |
| *SNAP23* | -0.13 | 3.45E-02 | Non-documented gene |
| *KBTBD8* | -0.13 | 3.47E-02 | Non-documented gene |
| *CDC40* | -0.13 | 3.47E-02 | Non-documented gene |
| *INPP4A* | -0.13 | 3.48E-02 | Non-documented gene |
| *EREG* | -0.13 | 3.49E-02 | Non-documented gene |
| *PAK1* | -0.13 | 3.50E-02 | Non-documented gene |
| *WDR26* | -0.13 | 3.51E-02 | Non-documented gene |
| *MAN1A1* | -0.13 | 3.52E-02 | Non-documented gene |
| *ANGPTL2* | -0.13 | 3.52E-02 | Non-documented gene |
| *VTI1B* | -0.13 | 3.53E-02 | Non-documented gene |
| *ANKRD36B* | -0.13 | 3.54E-02 | Non-documented gene |
| *APOF* | -0.13 | 3.55E-02 | Non-documented gene |
| *TSTA3* | -0.13 | 3.55E-02 | Non-documented gene |
| *SSX4B* | -0.13 | 3.56E-02 | Non-documented gene |
| *PDIK1L* | -0.13 | 3.57E-02 | Non-documented gene |
| *THOC4* | -0.13 | 3.58E-02 | Non-documented gene |
| *MAN2A1* | -0.13 | 3.58E-02 | Non-documented gene |
| *PRR19* | -0.13 | 3.59E-02 | Non-documented gene |
| *NOL8* | -0.13 | 3.60E-02 | Non-documented gene |
| *DHRS7B* | -0.13 | 3.61E-02 | Non-documented gene |
| *DCP1A* | -0.13 | 3.61E-02 | Non-documented gene |
| *FFAR2* | -0.13 | 3.62E-02 | Non-documented gene |
| *KHDRBS1* | -0.13 | 3.62E-02 | Non-documented gene |
| *AHCYL2* | -0.13 | 3.63E-02 | Non-documented gene |
| *YBX1* | -0.13 | 3.64E-02 | Non-documented gene |
| *BPTF* | -0.13 | 3.65E-02 | Non-documented gene |
| *SETD6* | -0.13 | 3.66E-02 | Non-documented gene |
| *AP1S2* | -0.13 | 3.66E-02 | Non-documented gene |
| *PIGA* | -0.13 | 3.66E-02 | Non-documented gene |
| *SLC2A1* | -0.13 | 3.67E-02 | Non-documented gene |
| *PCTP* | -0.13 | 3.69E-02 | Non-documented gene |
| *EPRS* | -0.13 | 3.70E-02 | Non-documented gene |
| *NDRG4* | -0.13 | 3.70E-02 | Non-documented gene |
| *NFYC* | -0.13 | 3.71E-02 | Non-documented gene |
| *SLCO2B1* | -0.13 | 3.72E-02 | Non-documented gene |
| *PRO1853* | -0.13 | 3.73E-02 | Non-documented gene |
| *ARL14* | -0.13 | 3.75E-02 | Non-documented gene |
| *ZMAT2* | -0.13 | 3.75E-02 | Non-documented gene |
| *SELO* | -0.13 | 3.76E-02 | Non-documented gene |
| *ABHD8* | -0.13 | 3.77E-02 | Non-documented gene |
| *PIPOX* | -0.13 | 3.77E-02 | Non-documented gene |
| *TMED8* | -0.13 | 3.78E-02 | Non-documented gene |
| *GNS* | -0.13 | 3.79E-02 | Non-documented gene |
| *MAGED1* | -0.13 | 3.80E-02 | Non-documented gene |
| *LAPTM5* | -0.13 | 3.81E-02 | Non-documented gene |
| *TRIP12* | -0.13 | 3.81E-02 | Non-documented gene |
| *CYC1* | -0.13 | 3.81E-02 | Non-documented gene |
| *RAPSN* | -0.13 | 3.82E-02 | Non-documented gene |
| *CR1* | -0.13 | 3.83E-02 | Non-documented gene |
| *MYD88* | -0.13 | 3.84E-02 | Non-documented gene |
| *KLHL22* | -0.13 | 3.84E-02 | Non-documented gene |
| *SULT1A1* | -0.13 | 3.85E-02 | Non-documented gene |
| *MAGEL2* | -0.13 | 3.87E-02 | Non-documented gene |
| *TICAM2* | -0.13 | 3.87E-02 | Non-documented gene |
| *NISCH* | -0.13 | 3.88E-02 | Non-documented gene |
| *ADRA2B* | -0.13 | 3.88E-02 | Non-documented gene |
| *RAB5B* | -0.13 | 3.89E-02 | Non-documented gene |
| *ANAPC11* | -0.13 | 3.90E-02 | Non-documented gene |
| *MAFB* | -0.14 | 3.92E-02 | Non-documented gene |
| *RASA1* | -0.14 | 3.92E-02 | Non-documented gene |
| *PSCD2* | -0.14 | 3.93E-02 | Non-documented gene |
| *ZC3HAV1* | -0.14 | 3.94E-02 | Non-documented gene |
| *CDCA5* | -0.14 | 3.95E-02 | Non-documented gene |
| *ZEB2* | -0.14 | 3.96E-02 | Reported gene |
| *GPR1* | -0.14 | 3.96E-02 | Non-documented gene |
| *MRPS17* | -0.14 | 3.97E-02 | Non-documented gene |
| *PNMA1* | -0.14 | 3.98E-02 | Non-documented gene |
| *CHMP2A* | -0.14 | 3.99E-02 | Non-documented gene |
| *SOX4* | -0.14 | 3.99E-02 | Non-documented gene |
| *VAC14* | -0.14 | 4.00E-02 | Non-documented gene |
| *GOPC* | -0.14 | 4.01E-02 | Non-documented gene |
| *SDHD* | -0.14 | 4.02E-02 | Non-documented gene |
| *CDC14A* | -0.14 | 4.03E-02 | Non-documented gene |
| *CAPN7* | -0.14 | 4.03E-02 | Non-documented gene |
| *ERAL1* | -0.14 | 4.03E-02 | Non-documented gene |
| *SLC30A1* | -0.14 | 4.04E-02 | Non-documented gene |
| *SERPINB5* | -0.14 | 4.05E-02 | Non-documented gene |
| *GOLGA8F* | -0.14 | 4.07E-02 | Non-documented gene |
| *SFRS2IP* | -0.14 | 4.08E-02 | Non-documented gene |
| *SSR2* | -0.14 | 4.08E-02 | Non-documented gene |
| *MBD6* | -0.14 | 4.10E-02 | Non-documented gene |
| *KRTAP5-11* | -0.14 | 4.10E-02 | Non-documented gene |
| *LIN28B* | -0.14 | 4.10E-02 | Non-documented gene |
| *ARMET* | -0.14 | 4.11E-02 | Non-documented gene |
| *SERINC1* | -0.14 | 4.12E-02 | Non-documented gene |
| *KLHDC8B* | -0.14 | 4.13E-02 | Non-documented gene |
| *WNT16* | -0.14 | 4.14E-02 | Non-documented gene |
| *TTTY19* | -0.14 | 4.15E-02 | Non-documented gene |
| *ARMCX3* | -0.14 | 4.16E-02 | Non-documented gene |
| *USP39* | -0.14 | 4.17E-02 | Non-documented gene |
| *TTTY14* | -0.14 | 4.17E-02 | Non-documented gene |
| *RPL15* | -0.14 | 4.18E-02 | Non-documented gene |
| *GPSM3* | -0.14 | 4.18E-02 | Non-documented gene |
| *NUBP1* | -0.14 | 4.20E-02 | Non-documented gene |
| *FBXO11* | -0.14 | 4.20E-02 | Non-documented gene |
| *ADD3* | -0.14 | 4.21E-02 | Non-documented gene |
| *TBL1X* | -0.14 | 4.22E-02 | Non-documented gene |
| *PRAMEF5* | -0.14 | 4.22E-02 | Non-documented gene |
| *DDX60* | -0.14 | 4.23E-02 | Non-documented gene |
| *CIDEA* | -0.14 | 4.24E-02 | Non-documented gene |
| *TEX101* | -0.14 | 4.25E-02 | Non-documented gene |
| *ZNF620* | -0.14 | 4.25E-02 | Non-documented gene |
| *PSMC6* | -0.14 | 4.26E-02 | Non-documented gene |
| *GOLGA3* | -0.14 | 4.27E-02 | Non-documented gene |
| *PFKFB3* | -0.14 | 4.28E-02 | Non-documented gene |
| *ZFP36L2* | -0.14 | 4.29E-02 | Non-documented gene |
| *RPS6KC1* | -0.14 | 4.29E-02 | Non-documented gene |
| *NEUROG3* | -0.14 | 4.30E-02 | Non-documented gene |
| *EXOC6* | -0.14 | 4.31E-02 | Reported gene |
| *HSPB2* | -0.14 | 4.32E-02 | Non-documented gene |
| *AQP9* | -0.14 | 4.32E-02 | Non-documented gene |
| *G0S2* | -0.14 | 4.33E-02 | Non-documented gene |
| *DKFZP564C196* | -0.14 | 4.35E-02 | Non-documented gene |
| *LIPJ* | -0.14 | 4.35E-02 | Non-documented gene |
| *SUCLA2* | -0.14 | 4.36E-02 | Non-documented gene |
| *ZCCHC17* | -0.14 | 4.36E-02 | Non-documented gene |
| *PPM1K* | -0.14 | 4.37E-02 | Non-documented gene |
| *COQ10A* | -0.14 | 4.39E-02 | Non-documented gene |
| *ANKRD11* | -0.14 | 4.39E-02 | Non-documented gene |
| *RBL2* | -0.15 | 4.40E-02 | Non-documented gene |
| *NUMB* | -0.15 | 4.40E-02 | Reported gene |
| *TEPP* | -0.15 | 4.40E-02 | Non-documented gene |
| *CCNG1* | -0.15 | 4.41E-02 | Non-documented gene |
| *EPPB9* | -0.15 | 4.43E-02 | Non-documented gene |
| *SCEL* | -0.15 | 4.43E-02 | Non-documented gene |
| *TRIML2* | -0.15 | 4.44E-02 | Non-documented gene |
| *SLFN12* | -0.15 | 4.45E-02 | Non-documented gene |
| *DTWD1* | -0.15 | 4.47E-02 | Non-documented gene |
| *NOL14* | -0.15 | 4.47E-02 | Non-documented gene |
| *LY6G6C* | -0.15 | 4.47E-02 | Non-documented gene |
| *MACF1* | -0.15 | 4.48E-02 | Non-documented gene |
| *GLCE* | -0.15 | 4.49E-02 | Non-documented gene |
| *CRTC2* | -0.15 | 4.50E-02 | Non-documented gene |
| *NOL11* | -0.15 | 4.51E-02 | Non-documented gene |
| *TBPL1* | -0.15 | 4.51E-02 | Non-documented gene |
| *PRKAR2B* | -0.15 | 4.52E-02 | Non-documented gene |
| *SH2D5* | -0.15 | 4.53E-02 | Non-documented gene |
| *TSC1* | -0.15 | 4.54E-02 | Non-documented gene |
| *SHQ1* | -0.15 | 4.55E-02 | Non-documented gene |
| *PRPF38B* | -0.15 | 4.55E-02 | Non-documented gene |
| *CBWD1* | -0.15 | 4.56E-02 | Non-documented gene |
| *DGAT2* | -0.15 | 4.57E-02 | Non-documented gene |
| *CPSF2* | -0.15 | 4.58E-02 | Non-documented gene |
| *DLG1* | -0.15 | 4.58E-02 | Non-documented gene |
| *TRIM21* | -0.15 | 4.58E-02 | Non-documented gene |
| *EPS15L1* | -0.15 | 4.60E-02 | Non-documented gene |
| *NXF4* | -0.15 | 4.61E-02 | Non-documented gene |
| *TMEM59* | -0.15 | 4.62E-02 | Non-documented gene |
| *INTS7* | -0.15 | 4.62E-02 | Non-documented gene |
| *TXNDC6* | -0.15 | 4.63E-02 | Non-documented gene |
| *ZFYVE21* | -0.15 | 4.64E-02 | Non-documented gene |
| *GBP4* | -0.15 | 4.65E-02 | Non-documented gene |
| *TRMT1* | -0.15 | 4.66E-02 | Non-documented gene |
| *FAM71A* | -0.15 | 4.66E-02 | Non-documented gene |
| *CYP4Z2P* | -0.15 | 4.68E-02 | Non-documented gene |
| *SAE1* | -0.15 | 4.68E-02 | Non-documented gene |
| *EIF4A3* | -0.15 | 4.69E-02 | Non-documented gene |
| *FAM62A* | -0.15 | 4.69E-02 | Non-documented gene |
| *NUP88* | -0.15 | 4.70E-02 | Non-documented gene |
| *CAMP* | -0.15 | 4.72E-02 | Non-documented gene |
| *ZNF497* | -0.15 | 4.72E-02 | Non-documented gene |
| *SFRS12* | -0.15 | 4.73E-02 | Non-documented gene |
| *PRUNE2* | -0.15 | 4.73E-02 | Non-documented gene |
| *XPO6* | -0.15 | 4.74E-02 | Non-documented gene |
| *RPL39* | -0.15 | 4.75E-02 | Non-documented gene |
| *RBM12* | -0.15 | 4.76E-02 | Non-documented gene |
| *FST* | -0.15 | 4.77E-02 | Non-documented gene |
| *IGBP1* | -0.15 | 4.77E-02 | Non-documented gene |
| *BPHL* | -0.15 | 4.78E-02 | Non-documented gene |
| *CNNM4* | -0.15 | 4.79E-02 | Non-documented gene |
| *MRPS35* | -0.15 | 4.81E-02 | Non-documented gene |
| *TRIM69* | -0.15 | 4.81E-02 | Non-documented gene |
| *ZNF133* | -0.15 | 4.81E-02 | Non-documented gene |
| *BAD* | -0.15 | 4.82E-02 | Non-documented gene |
| *TSPAN10* | -0.15 | 4.83E-02 | Non-documented gene |
| *ACAT2* | -0.15 | 4.84E-02 | Non-documented gene |
| *RIOK1* | -0.15 | 4.84E-02 | Non-documented gene |
| *TSG101* | -0.15 | 4.85E-02 | Non-documented gene |
| *ASTL* | -0.15 | 4.86E-02 | Non-documented gene |
| *UBE2H* | -0.15 | 4.87E-02 | Non-documented gene |
| *RPL27A* | -0.15 | 4.88E-02 | Non-documented gene |
| *RPS15* | -0.15 | 4.88E-02 | Non-documented gene |
| *TAOK1* | -0.15 | 4.89E-02 | Non-documented gene |
| *HHEX* | -0.15 | 4.90E-02 | Non-documented gene |
| *ZBTB34* | -0.15 | 4.91E-02 | Non-documented gene |
| *ATP5G2* | -0.16 | 4.92E-02 | Non-documented gene |
| *SDSL* | -0.16 | 4.92E-02 | Non-documented gene |
| *RTN3* | -0.16 | 4.94E-02 | Non-documented gene |
| *RPL9* | -0.16 | 4.95E-02 | Non-documented gene |
| *HNRNPD* | -0.16 | 4.95E-02 | Non-documented gene |
| *ERGIC3* | -0.16 | 4.96E-02 | Non-documented gene |
| *OR5M10* | -0.16 | 4.96E-02 | Non-documented gene |
| *EXOC1* | -0.16 | 4.97E-02 | Non-documented gene |
| *CLDN18* | -0.16 | 4.98E-02 | Non-documented gene |
| *VPS36* | -0.16 | 4.99E-02 | Non-documented gene |
| *BCL6B* | -0.16 | 4.99E-02 | Non-documented gene |
