# Supplementary material for: Integration of summary data from GWAS and eQTL studies identified novel risk genes for coronary artery disease
Source: Medicine (Baltimore). 2021 Mar 19;100(11):e24769. doi: 10.1097/MD.0000000000024769 (PMC7982177; doi:10.1097/MD.0000000000024769)
Supplement: Supplemental Digital Content [file medi-100-e24769-s003.docx]

**Supplemental Table S9. Significant gene sets related to disease based on the Disgenet database enriched by CAD-associated genes identified from Sherlock Bayesian analysis**

| **Gene Set** | **Description** | **Size** | **Expect** | **Ratio** | **P value** |
| --- | --- | --- | --- | --- | --- |
| C0423110 | Downward slant of palpebral fissure | 159 | 5.02 | 2.99 | 1.40E-04 |
| C0240083 | joint abnormality | 5 | 0.16 | 19.01 | 2.96E-04 |
| C1839271 | Birth length greater than 97th percentile | 5 | 0.16 | 19.01 | 2.96E-04 |
| C1458155 | Mammary Neoplasms | 425 | 13.41 | 2.01 | 3.75E-04 |
| C0206620 | Lymphangioma, Cystic | 21 | 0.66 | 7.55 | 4.04E-04 |
| C0240635 | Byzanthine arch palate | 195 | 6.15 | 2.60 | 4.22E-04 |
| C0544886 | Somatic mutation | 62 | 1.96 | 4.09 | 6.78E-04 |
| C1837732 | Thickened helices | 15 | 0.47 | 8.45 | 1.00E-03 |
| C0025990 | Micrognathism | 276 | 8.71 | 2.18 | 1.11E-03 |
| C0240295 | Mandibular hypoplasia | 276 | 8.71 | 2.18 | 1.11E-03 |
