# Supplementary material for: Integration of summary data from GWAS and eQTL studies identified novel risk genes for coronary artery disease
Source: Medicine (Baltimore). 2021 Mar 19;100(11):e24769. doi: 10.1097/MD.0000000000024769 (PMC7982177; doi:10.1097/MD.0000000000024769)
Supplement: Supplemental Digital Content [file medi-100-e24769-s004.docx]

**Supplemental Table S10. Significant gene sets related to disease based on the GLAD4U database enriched by CAD-associated genes identified from Sherlock Bayesian analysis**

| **Gene Set** | **Description** | **Size** | **Expect** | **Ratio** | **P value** |
| --- | --- | --- | --- | --- | --- |
| PA165108956 | Parkes Weber syndrome | 7 | 0.22 | 13.58 | 9.95E-04 |
| PA165109076 | Unspecified injury of hand | 17 | 0.54 | 7.45 | 1.68E-03 |
| PA444679 | Ketosis | 31 | 0.98 | 5.11 | 2.64E-03 |
| PA165108622 | Drug interaction with drug | 484 | 15.28 | 1.77 | 3.09E-03 |
| PA443653 | Cell Transformation, Neoplastic | 307 | 9.69 | 1.96 | 4.13E-03 |
| PA446254 | Choroid Diseases | 65 | 2.05 | 3.41 | 4.28E-03 |
| PA447230 | HIV | 854 | 26.96 | 1.52 | 4.86E-03 |
| PA446687 | Gliosarcoma | 12 | 0.38 | 7.92 | 5.56E-03 |
| PA165108899 | Hypertensive encephalopathy | 12 | 0.38 | 7.92 | 5.56E-03 |
| PA165108227 | Juvenile chronic myeloid leukaemia | 25 | 0.79 | 5.07 | 7.33E-03 |
