# Supplementary material for: Integration of summary data from GWAS and eQTL studies identified novel risk genes for coronary artery disease
Source: Medicine (Baltimore). 2021 Mar 19;100(11):e24769. doi: 10.1097/MD.0000000000024769 (PMC7982177; doi:10.1097/MD.0000000000024769)
Supplement: Supplemental Digital Content [file medi-100-e24769-s005.pptx]

## Slide 1
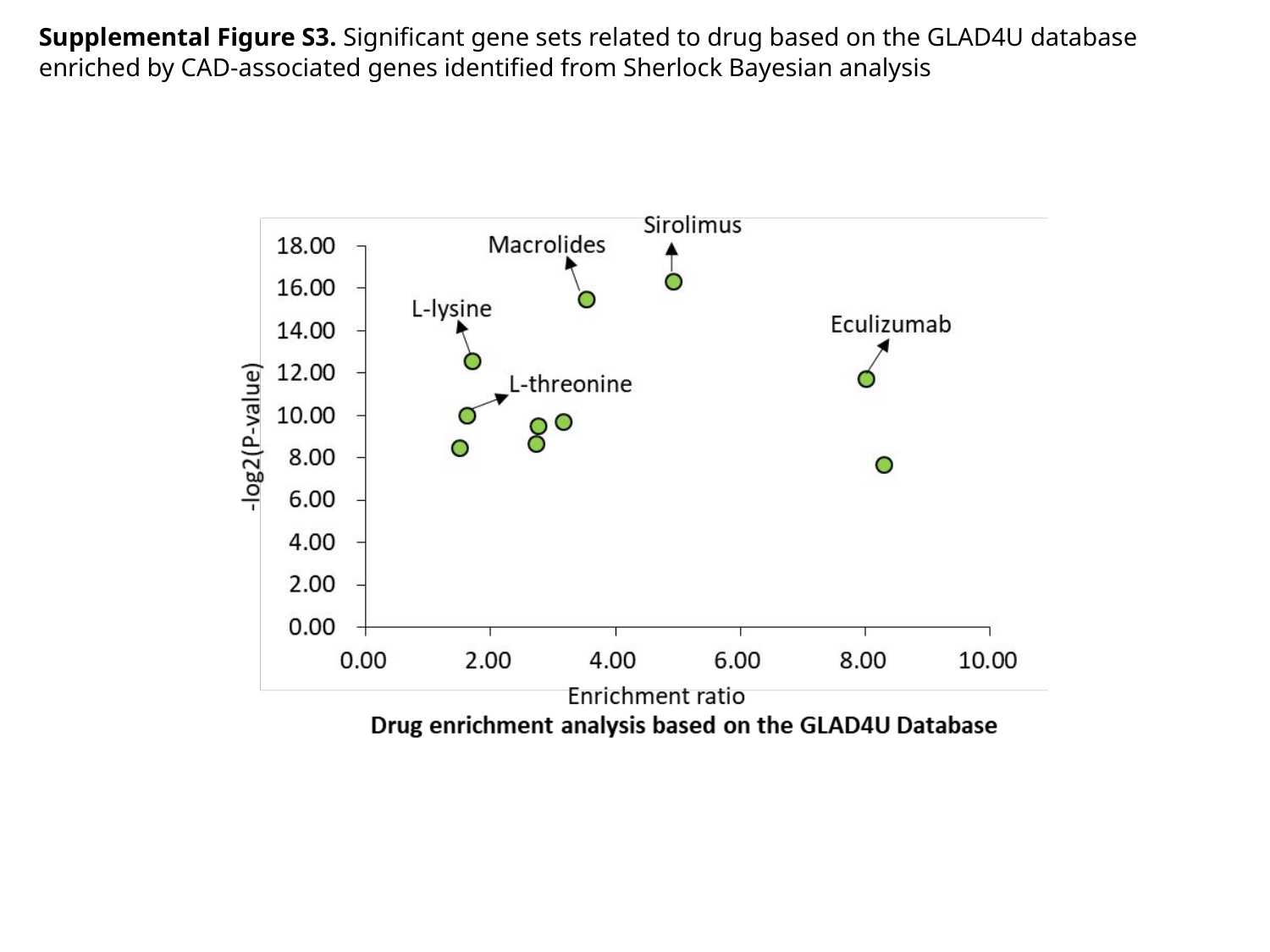

Supplemental Figure S3. Significant gene sets related to drug based on the GLAD4U database enriched by CAD-associated genes identified from Sherlock Bayesian analysis
