# Supplementary material for: Integration of summary data from GWAS and eQTL studies identified novel risk genes for coronary artery disease
Source: Medicine (Baltimore). 2021 Mar 19;100(11):e24769. doi: 10.1097/MD.0000000000024769 (PMC7982177; doi:10.1097/MD.0000000000024769)
Supplement: Supplemental Digital Content [file medi-100-e24769-s006.docx]

**Supplemental Table S11. Significant gene sets related to drug based on the GLAD4U database enriched by CAD-associated genes identified from Sherlock Bayesian analysis**

| **Gene Set** | **Description** | **Size** | **Expect** | **Ratio** | **P Value** |
| --- | --- | --- | --- | --- | --- |
| PA451365 | sirolimus | 68 | 2.24 | 4.92 | 1.21E-05 |
| PA164712882 | Macrolides | 129 | 4.24 | 3.54 | 2.15E-05 |
| PA450280 | l-lysine | 874 | 28.74 | 1.71 | 1.62E-04 |
| PA164776637 | eculizumab | 19 | 0.62 | 8.00 | 2.98E-04 |
| PA451673 | l-threonine | 848 | 27.88 | 1.61 | 9.71E-04 |
| PA164713094 | Other immunosuppressants | 96 | 3.16 | 3.17 | 1.20E-03 |
| PA449783 | glycerin | 132 | 4.34 | 2.76 | 1.35E-03 |
| PA164712561 | Bioflavonoids | 123 | 4.04 | 2.72 | 2.41E-03 |
| PA451330 | l-serine | 970 | 31.90 | 1.50 | 2.80E-03 |
| PA164746890 | temsirolimus | 11 | 0.36 | 8.29 | 4.78E-03 |
