# Supplementary material for: Integration of summary data from GWAS and eQTL studies identified novel risk genes for coronary artery disease
Source: Medicine (Baltimore). 2021 Mar 19;100(11):e24769. doi: 10.1097/MD.0000000000024769 (PMC7982177; doi:10.1097/MD.0000000000024769)
Supplement: Supplemental Digital Content [file medi-100-e24769-s007.docx]

**Supplemental Table S12. 72 Sherlock-identified genes from discovery samples (Zeller et al. eQTL dataset) overlapped with MAGMA-identified genes**

| **Gene name** | **LBF** | **Sherlock-identified P-value (Zeller et al. Dataset #3)** | **MAGMA-identified P-value (Dataset #1)** | **MAGMA-identified P value (Dataset #2, negative control)** | **GWAS Catalog documented genes** |
| --- | --- | --- | --- | --- | --- |
| *WNT2* | -0.04 | 6.96E-04 | 1.29E-04 | 0.424 | Non-documented gene |
| *CCHCR1* | -0.04 | 1.95E-03 | 4.86E-03 | 0.790 | Non-documented gene |
| *DNAJC10* | -0.05 | 2.35E-03 | 2.58E-02 | 0.963 | Non-documented gene |
| *ADAM17* | -0.05 | 2.43E-03 | 3.48E-02 | 0.133 | Non-documented gene |
| *ATMIN* | -0.05 | 2.59E-03 | 2.75E-02 | 0.267 | Non-documented gene |
| *CCDC97* | -0.05 | 5.58E-03 | 5.25E-06 | 0.347 | Reported gene |
| *FOXP1* | -0.05 | 5.74E-03 | 2.11E-03 | 0.261 | Non-documented gene |
| *CABIN1* | -0.06 | 7.87E-03 | 3.62E-03 | 0.232 | Reported gene |
| *PLEKHM1* | -0.06 | 8.10E-03 | 2.62E-02 | 0.836 | Non-documented gene |
| *GOLGA2* | -0.06 | 8.18E-03 | 4.03E-02 | 0.422 | Non-documented gene |
| *GJC3* | -0.06 | 8.26E-03 | 6.81E-05 | 0.542 | Non-documented gene |
| *SMARCA5* | -0.06 | 8.89E-03 | 1.74E-02 | 0.652 | Non-documented gene |
| *FEN1* | -0.07 | 1.24E-02 | 6.16E-04 | 0.123 | Non-documented gene |
| *ANXA9* | -0.07 | 1.38E-02 | 7.78E-05 | 0.905 | Non-documented gene |
| *PMVK* | -0.07 | 1.38E-02 | 1.67E-02 | 0.181 | Non-documented gene |
| *SCUBE2* | -0.07 | 1.40E-02 | 1.62E-03 | 0.880 | Non-documented gene |
| *SEMA3F* | -0.08 | 1.48E-02 | 8.43E-03 | 0.301 | Non-documented gene |
| *KDR* | -0.08 | 1.62E-02 | 4.31E-02 | 0.045 | Non-documented gene |
| *DRG2* | -0.08 | 1.63E-02 | 1.88E-05 | 0.181 | Non-documented gene |
| *MAD2L1* | -0.09 | 1.70E-02 | 1.13E-02 | 0.636 | Reported gene |
| *CD70* | -0.09 | 1.71E-02 | 4.08E-02 | 0.892 | Non-documented gene |
| *PTPN11* | -0.09 | 1.80E-02 | 1.22E-04 | 0.469 | Non-documented gene |
| *SBF2* | -0.10 | 1.99E-02 | 2.95E-04 | 0.588 | Non-documented gene |
| *ZNF282* | -0.10 | 1.99E-02 | 1.12E-02 | 0.525 | Non-documented gene |
| *CHCHD1* | -0.10 | 2.04E-02 | 1.82E-03 | 0.736 | Non-documented gene |
| *SPC24* | -0.10 | 2.14E-02 | 2.08E-05 | 0.635 | Non-documented gene |
| *SF3A3* | -0.10 | 2.18E-02 | 4.44E-03 | 0.966 | Reported gene |
| *PHB* | -0.10 | 2.27E-02 | 6.96E-05 | 0.677 | Reported gene |
| *TUBG1* | -0.10 | 2.28E-02 | 3.12E-04 | 0.030 | Non-documented gene |
| *AIG1* | -0.10 | 2.32E-02 | 1.15E-02 | 0.286 | Non-documented gene |
| *PECAM1* | -0.10 | 2.36E-02 | 1.07E-05 | 0.001 | Reported gene |
| *UBE2W* | -0.11 | 2.37E-02 | 3.01E-03 | 0.665 | Non-documented gene |
| *RIC8A* | -0.11 | 2.39E-02 | 4.42E-03 | 0.776 | Non-documented gene |
| *ACTR10* | -0.11 | 2.51E-02 | 2.02E-02 | 0.712 | Non-documented gene |
| *MRGPRF* | -0.11 | 2.54E-02 | 1.96E-03 | 0.101 | Non-documented gene |
| *LHX6* | -0.11 | 2.55E-02 | 8.27E-04 | 0.875 | Non-documented gene |
| *RNF38* | -0.11 | 2.63E-02 | 3.74E-02 | 0.347 | Non-documented gene |
| *ZNF3* | -0.11 | 2.72E-02 | 3.73E-03 | 0.234 | Non-documented gene |
| *LPIN3* | -0.11 | 2.74E-02 | 1.96E-03 | 0.054 | Non-documented gene |
| *IGF2R* | -0.11 | 2.75E-02 | 3.24E-07 | 0.562 | Reported gene |
| *DIAPH1* | -0.12 | 2.85E-02 | 1.72E-03 | 0.328 | Non-documented gene |
| *MAN2A2* | -0.12 | 2.95E-02 | 2.93E-12 | 0.684 | Non-documented gene |
| *SIPA1* | -0.12 | 3.00E-02 | 1.57E-04 | 0.355 | Non-documented gene |
| *TULP3* | -0.12 | 3.06E-02 | 2.69E-03 | 0.562 | Non-documented gene |
| *CFB* | -0.12 | 3.10E-02 | 7.79E-03 | 0.528 | Reported gene |
| *GNAI2* | -0.12 | 3.10E-02 | 2.86E-02 | 0.217 | Non-documented gene |
| *GFI1* | -0.12 | 3.20E-02 | 5.11E-04 | 0.951 | Non-documented gene |
| *KPNB1* | -0.12 | 3.28E-02 | 3.81E-02 | 0.256 | Non-documented gene |
| *OCLM* | -0.12 | 3.28E-02 | 3.29E-02 | 2.87E-04 | Non-documented gene |
| *RNF181* | -0.13 | 3.33E-02 | 1.75E-11 | 0.208 | Non-documented gene |
| *EHMT1* | -0.13 | 3.42E-02 | 4.73E-02 | NA | Non-documented gene |
| *PAK1* | -0.13 | 3.50E-02 | 1.12E-03 | 0.103 | Non-documented gene |
| *APOF* | -0.13 | 3.55E-02 | 3.23E-06 | 0.468 | Non-documented gene |
| *BPTF* | -0.13 | 3.65E-02 | 9.34E-03 | 0.088 | Non-documented gene |
| *CDCA5* | -0.14 | 3.95E-02 | 4.05E-02 | 0.277 | Non-documented gene |
| *ZEB2* | -0.14 | 3.96E-02 | 7.26E-06 | 0.349 | Reported gene |
| *MRPS17* | -0.14 | 3.97E-02 | 9.74E-03 | 0.679 | Non-documented gene |
| *CAPN7* | -0.14 | 4.03E-02 | 4.27E-02 | 0.841 | Non-documented gene |
| *ERAL1* | -0.14 | 4.03E-02 | 5.65E-03 | 0.567 | Non-documented gene |
| *USP39* | -0.14 | 4.17E-02 | 4.02E-10 | 0.008 | Non-documented gene |
| *DDX60* | -0.14 | 4.23E-02 | 2.12E-02 | 0.963 | Non-documented gene |
| *GOLGA3* | -0.14 | 4.27E-02 | 3.87E-02 | NA | Non-documented gene |
| *ZFP36L2* | -0.14 | 4.29E-02 | 6.09E-07 | 0.606 | Non-documented gene |
| *COQ10A* | -0.14 | 4.39E-02 | 6.90E-05 | 0.667 | Non-documented gene |
| *RBL2* | -0.15 | 4.40E-02 | 2.56E-02 | 0.597 | Non-documented gene |
| *LY6G6C* | -0.15 | 4.47E-02 | 3.48E-02 | 0.920 | Non-documented gene |
| *CAMP* | -0.15 | 4.72E-02 | 1.46E-02 | NA | Non-documented gene |
| *XPO6* | -0.15 | 4.74E-02 | 1.42E-02 | 0.859 | Non-documented gene |
| *RBM12* | -0.15 | 4.76E-02 | 2.61E-02 | NA | Non-documented gene |
| *UBE2H* | -0.15 | 4.87E-02 | 2.43E-02 | 0.486 | Non-documented gene |
| *HNRNPD* | -0.16 | 4.95E-02 | 1.21E-02 | 0.737 | Non-documented gene |
| *OR5M10* | -0.16 | 4.96E-02 | 1.75E-02 | 0.550 | Non-documented gene |

Note: NA means not applicable
