# Supplementary material for: Integration of summary data from GWAS and eQTL studies identified novel risk genes for coronary artery disease
Source: Medicine (Baltimore). 2021 Mar 19;100(11):e24769. doi: 10.1097/MD.0000000000024769 (PMC7982177; doi:10.1097/MD.0000000000024769)
Supplement: Supplemental Digital Content [file medi-100-e24769-s008.docx]

**Supplemental Table S14. Sherlock- and MAGMA-identified genes reported in previous GWASs**

| **Gene name** | **P values from Sherlock/MAGMA** | **Geneset resource** | **Reported in Previous GWAS** |
| --- | --- | --- | --- |
| *HERPUD1* | 4.95E-03 | Geneset #1 | Reported gene |
| *CCDC97* | 5.58E-03 | Geneset #1 | Reported gene |
| *CABIN1* | 7.87E-03 | Geneset #1 | Reported gene |
| *MAD2L1* | 1.70E-02 | Geneset #1 | Reported gene |
| *HMOX1* | 1.87E-02 | Geneset #1 | Reported gene |
| *MAP3K1* | 2.10E-02 | Geneset #1 | Reported gene |
| *SF3A3* | 2.18E-02 | Geneset #1 | Reported gene |
| *PHB* | 2.27E-02 | Geneset #1 | Reported gene |
| *PECAM1* | 2.36E-02 | Geneset #1 | Reported gene |
| *IGF2R* | 2.75E-02 | Geneset #1 | Reported gene |
| *CFB* | 3.10E-02 | Geneset #1 | Reported gene |
| *RNF4* | 3.36E-02 | Geneset #1 | Reported gene |
| *ZEB2* | 3.96E-02 | Geneset #1 | Reported gene |
| *EXOC6* | 4.31E-02 | Geneset #1 | Reported gene |
| *NUMB* | 4.40E-02 | Geneset #1 | Reported gene |
| *HSF2BP* | 3.12E-03 | Geneset #2 | Reported gene |
| *XKR4* | 5.17E-03 | Geneset #2 | Reported gene |
| *PSRC1* | 6.62E-03 | Geneset #2 | Reported gene |
| *MPHOSPH10* | 8.59E-03 | Geneset #2 | Reported gene |
| *TOMM40* | 1.07E-02 | Geneset #2 | Reported gene |
| *USP43* | 1.15E-02 | Geneset #2 | Reported gene |
| *TRIM22* | 1.16E-02 | Geneset #2 | Reported gene |
| *RASD1* | 1.19E-02 | Geneset #2 | Reported gene |
| *GEM* | 1.32E-02 | Geneset #2 | Reported gene |
| *OBFC1* | 1.92E-02 | Geneset #2 | Reported gene |
| *HMOX1* | 2.15E-02 | Geneset #2 | Reported gene |
| *CORO6* | 2.18E-02 | Geneset #2 | Reported gene |
| *FBXO15* | 2.19E-02 | Geneset #2 | Reported gene |
| *ANKRD13B* | 2.41E-02 | Geneset #2 | Reported gene |
| *TBX20* | 2.65E-02 | Geneset #2 | Reported gene |
| *SNF8* | 3.61E-02 | Geneset #2 | Reported gene |
| *SKIV2L* | 3.86E-02 | Geneset #2 | Reported gene |
| *HDGFL1* | 4.67E-02 | Geneset #2 | Reported gene |
| *CDKN2A* | 2.14E-39 | Geneset #3 | Reported gene |
| *CDKN2B* | 5.53E-34 | Geneset #3 | Reported gene |
| *PHACTR1* | 7.54E-20 | Geneset #3 | Reported gene |
| *PSRC1* | 1.04E-19 | Geneset #3 | Reported gene |
| *ADAMTS7* | 5.72E-19 | Geneset #3 | Reported gene |
| *LDLR* | 9.66E-18 | Geneset #3 | Reported gene |
| *LPA* | 2.34E-17 | Geneset #3 | Reported gene |
| *MORF4L1* | 4.06E-16 | Geneset #3 | Reported gene |
| *WDR12* | 1.98E-15 | Geneset #3 | Reported gene |
| *PLG* | 1.00E-14 | Geneset #3 | Reported gene |
| *CELSR2* | 1.74E-14 | Geneset #3 | Reported gene |
| *FURIN* | 1.97E-14 | Geneset #3 | Reported gene |
| *FES* | 2.48E-14 | Geneset #3 | Reported gene |
| *COL4A1* | 8.70E-14 | Geneset #3 | Reported gene |
| *MIA3* | 3.64E-13 | Geneset #3 | Reported gene |
| *NBEAL1* | 3.98E-13 | Geneset #3 | Reported gene |
| *MAT2A* | 8.32E-13 | Geneset #3 | Reported gene |
| *GGCX* | 1.01E-12 | Geneset #3 | Reported gene |
| *APOC1* | 1.29E-12 | Geneset #3 | Reported gene |
| *VAMP5* | 5.23E-12 | Geneset #3 | Reported gene |
| *KIAA1462* | 7.76E-12 | Geneset #3 | Reported gene |
| *APOE* | 1.06E-11 | Geneset #3 | Reported gene |
| *VAMP8* | 1.15E-11 | Geneset #3 | Reported gene |
| *LIPA* | 1.25E-11 | Geneset #3 | Reported gene |
| *SMARCA4* | 2.04E-11 | Geneset #3 | Reported gene |
| *AIDA* | 2.49E-11 | Geneset #3 | Reported gene |
| *SLC22A3* | 3.00E-11 | Geneset #3 | Reported gene |
| *SMG6* | 6.25E-11 | Geneset #3 | Reported gene |
| *TCF21* | 7.15E-11 | Geneset #3 | Reported gene |
| *TGFB1* | 1.90E-10 | Geneset #3 | Reported gene |
| *LPL* | 9.76E-10 | Geneset #3 | Reported gene |
| *TOMM40* | 1.70E-09 | Geneset #3 | Reported gene |
| *PPAP2B* | 1.70E-09 | Geneset #3 | Reported gene |
| *SKI* | 3.29E-09 | Geneset #3 | Reported gene |
| *MRAS* | 3.79E-09 | Geneset #3 | Reported gene |
| *ARNTL* | 4.29E-09 | Geneset #3 | Reported gene |
| *COL4A2* | 5.25E-09 | Geneset #3 | Reported gene |
| *TMEM170A* | 7.49E-09 | Geneset #3 | Reported gene |
| *ATXN2* | 1.48E-08 | Geneset #3 | Reported gene |
| *FGD5* | 2.02E-08 | Geneset #3 | Reported gene |
| *APOB* | 2.04E-08 | Geneset #3 | Reported gene |
| *HHIPL1* | 3.72E-08 | Geneset #3 | Reported gene |
| *BCAS3* | 4.01E-08 | Geneset #3 | Reported gene |
| *BCAR1* | 5.79E-08 | Geneset #3 | Reported gene |
| *IL6R* | 6.25E-08 | Geneset #3 | Reported gene |
| *CYP17A1* | 6.36E-08 | Geneset #3 | Reported gene |
| *B9D2* | 6.73E-08 | Geneset #3 | Reported gene |
| *CTAGE1* | 6.86E-08 | Geneset #3 | Reported gene |
| *AS3MT* | 7.02E-08 | Geneset #3 | Reported gene |
| *CFDP1* | 7.31E-08 | Geneset #3 | Reported gene |
| *HNF1A* | 1.16E-07 | Geneset #3 | Reported gene |
| *LINGO4* | 1.50E-07 | Geneset #3 | Reported gene |
| *HTRA1* | 2.60E-07 | Geneset #3 | Reported gene |
| *CTRB2* | 2.66E-07 | Geneset #3 | Reported gene |
| *HSPB9* | 2.85E-07 | Geneset #3 | Reported gene |
| *CYP46A1* | 2.98E-07 | Geneset #3 | Reported gene |
| *SH2B3* | 3.04E-07 | Geneset #3 | Reported gene |
| *IGF2R* | 3.24E-07 | Geneset #3 | Reported gene |
| *ABCG8* | 3.49E-07 | Geneset #3 | Reported gene |
| *EDNRA* | 3.71E-07 | Geneset #3 | Reported gene |
| *ABCG5* | 3.77E-07 | Geneset #3 | Reported gene |
| *GALNT4* | 4.06E-07 | Geneset #3 | Reported gene |
| *APOC4* | 4.12E-07 | Geneset #3 | Reported gene |
| *ZNF827* | 4.40E-07 | Geneset #3 | Reported gene |
| *BCAP29* | 4.94E-07 | Geneset #3 | Reported gene |
| *DHX58* | 5.04E-07 | Geneset #3 | Reported gene |
| *SNRPD2* | 6.44E-07 | Geneset #3 | Reported gene |
| *ATP2B1* | 6.68E-07 | Geneset #3 | Reported gene |
| *GIP* | 6.97E-07 | Geneset #3 | Reported gene |
| *SRR* | 7.88E-07 | Geneset #3 | Reported gene |
| *TCTA* | 7.90E-07 | Geneset #3 | Reported gene |
| *AMT* | 7.93E-07 | Geneset #3 | Reported gene |
| *CTRB1* | 9.43E-07 | Geneset #3 | Reported gene |
| *KCNJ13* | 9.87E-07 | Geneset #3 | Reported gene |
| *RHOA* | 1.22E-06 | Geneset #3 | Reported gene |
| *POC1B* | 1.39E-06 | Geneset #3 | Reported gene |
| *GUCY1A3* | 1.42E-06 | Geneset #3 | Reported gene |
| *FCHO1* | 1.46E-06 | Geneset #3 | Reported gene |
| *SNF8* | 1.62E-06 | Geneset #3 | Reported gene |
| *HDGFL1* | 1.73E-06 | Geneset #3 | Reported gene |
| *KAT2A* | 1.81E-06 | Geneset #3 | Reported gene |
| *RAI1* | 1.88E-06 | Geneset #3 | Reported gene |
| *NEK9* | 2.13E-06 | Geneset #3 | Reported gene |
| *SMAD3* | 2.40E-06 | Geneset #3 | Reported gene |
| *THADA* | 2.46E-06 | Geneset #3 | Reported gene |
| *RAB5C* | 2.61E-06 | Geneset #3 | Reported gene |
