# Supplementary material for: Integration of summary data from GWAS and eQTL studies identified novel risk genes for coronary artery disease
Source: Medicine (Baltimore). 2021 Mar 19;100(11):e24769. doi: 10.1097/MD.0000000000024769 (PMC7982177; doi:10.1097/MD.0000000000024769)
Supplement: Supplemental Digital Content [file medi-100-e24769-s009.docx]

**Supplemental Table S13. 29 Sherlock-identified genes from discovery samples (Zeller et al. eQTL dataset) overlapped with Sherlock-identified genes from replication samples (Dixon et al. eQTL dataset)**

| **Gene name** | **LBF** | **Sherlock-identified P-value (Zeller et al. Dataset #3)** | **Sherlock-**identified P-value (Dixon et al. Dataset #4) | **MAGMA-identified P value (Dataset #2, negative control)** | **GWAS Catalog documented genes** |
| --- | --- | --- | --- | --- | --- |
| *SCML2* | -0.04 | 4.58E-04 | 8.06E-03 | NA | Non-documented gene |
| *GPR173* | -0.04 | 1.25E-03 | 2.71E-02 | NA | Non-documented gene |
| *FREM2* | -0.05 | 3.29E-03 | 7.60E-03 | 0.308 | Non-documented gene |
| *GABARAP* | -0.05 | 4.95E-03 | 1.00E-02 | 0.286 | Non-documented gene |
| *UQCRFS1* | -0.05 | 5.82E-03 | 4.20E-02 | NA | Non-documented gene |
| *MAGEH1* | -0.06 | 8.81E-03 | 1.48E-02 | NA | Non-documented gene |
| *UNC5CL* | -0.07 | 1.09E-02 | 1.43E-02 | 0.186 | Non-documented gene |
| *MSI1* | -0.07 | 1.16E-02 | 4.11E-03 | 0.880 | Non-documented gene |
| *DENND1C* | -0.08 | 1.50E-02 | 5.02E-03 | 0.985 | Non-documented gene |
| *MARCKSL1* | -0.09 | 1.65E-02 | 1.70E-02 | 0.169 | Non-documented gene |
| *HMOX1* | -0.09 | 1.87E-02 | 2.15E-02 | 0.498 | Reported gene |
| *CHCHD1* | -0.10 | 2.04E-02 | 1.32E-02 | 0.736 | Non-documented gene |
| *TUBG1* | -0.10 | 2.28E-02 | 6.46E-03 | 0.030 | Non-documented gene |
| *ZP1* | -0.12 | 2.81E-02 | 4.39E-02 | 0.476 | Non-documented gene |
| *PI4K2B* | -0.12 | 3.02E-02 | 2.94E-02 | 0.627 | Non-documented gene |
| *SLC35B2* | -0.12 | 3.07E-02 | 1.60E-02 | 0.203 | Non-documented gene |
| *PPP2R1A* | -0.12 | 3.29E-02 | 2.26E-02 | 0.085 | Non-documented gene |
| *RAPSN* | -0.13 | 3.82E-02 | 2.05E-03 | 0.864 | Non-documented gene |
| *MAGEL2* | -0.13 | 3.87E-02 | 4.68E-02 | 0.062 | Non-documented gene |
| *MRPS17* | -0.14 | 3.97E-02 | 2.30E-02 | 0.679 | Non-documented gene |
| *LIN28B* | -0.14 | 4.10E-02 | 4.33E-02 | 0.549 | Non-documented gene |
| *PSMC6* | -0.14 | 4.26E-02 | 3.73E-03 | 0.357 | Non-documented gene |
| *SUCLA2* | -0.14 | 4.36E-02 | 1.51E-02 | 0.183 | Non-documented gene |
| *TEPP* | -0.15 | 4.40E-02 | 4.59E-02 | 0.302 | Non-documented gene |
| *LY6G6C* | -0.15 | 4.47E-02 | 1.06E-02 | 0.920 | Non-documented gene |
| *PRKAR2B* | -0.15 | 4.52E-02 | 4.21E-02 | 0.585 | Non-documented gene |
| *TMEM59* | -0.15 | 4.62E-02 | 2.11E-02 | 0.467 | Non-documented gene |
| *MRPS35* | -0.15 | 4.81E-02 | 9.58E-03 | 0.854 | Non-documented gene |
| *RPL27A* | -0.15 | 4.88E-02 | 2.97E-02 | 0.446 | Non-documented gene |

Note: NA means not applicable
