# Supplementary material for: Integration of summary data from GWAS and eQTL studies identified novel risk genes for coronary artery disease
Source: Medicine (Baltimore). 2021 Mar 19;100(11):e24769. doi: 10.1097/MD.0000000000024769 (PMC7982177; doi:10.1097/MD.0000000000024769)
Supplement: Supplemental Digital Content [file medi-100-e24769-s010.docx]

**Supplemental Table S15. Multiple eSNPs in each of these 4 identified genes for CAD.**

| **Gene name** | **SNP ID** | **CHR** | **Position** | **Proximity** | **eQTL P values** | **GWAS P values** | **LBF** | **Gene resources** |
| --- | --- | --- | --- | --- | --- | --- | --- | --- |
| ***CHCHD1*** | rs84314 | 1 | 33367799 | trans | 7.09E-06 | 8.16E-03 | -0.05 | Geneset #1 |
| ***CHCHD1*** | rs2206692 | 20 | 6826870 | trans | 7.76E-06 | 8.20E-03 | -0.05 | Geneset #1 |
| ***CHCHD1*** | rs153677 | 16 | 64431866 | trans | 9.10E-06 | 8.58E-03 | -0.07 | Geneset #2 |
| ***TUBG1*** | rs1975283 | 1 | 117944081 | trans | 6.67E-06 | 9.09E-03 | -0.06 | Geneset #1 |
| ***TUBG1*** | rs1422601 | 5 | 120362701 | trans | 8.38E-06 | 8.88E-03 | -0.05 | Geneset #1 |
| ***TUBG1*** | rs1035665 | 8 | 72766586 | trans | 9.90E-06 | 8.25E-03 | -0.06 | Geneset #2 |
| ***MRPS17*** | rs2172110 | 3 | 4361378 | trans | 8.80E-06 | 1.09E-02 | -0.06 | Geneset #1 |
| ***MRPS17*** | rs17761403 | 9 | 27113348 | trans | 4.73E-06 | 1.36E-02 | -0.08 | Geneset #1 |
| ***MRPS17*** | rs1043991 | 9 | 110932820 | trans | 8.20E-06 | 8.35E-03 | -0.07 | Geneset #2 |
| ***LY6G6C*** | rs12049288 | 1 | 186757122 | trans | 9.34E-06 | 8.23E-03 | -0.04 | Geneset #1 |
| ***LY6G6C*** | rs16990865 | 4 | 34916561 | trans | 9.39E-06 | 1.24E-02 | -0.06 | Geneset #1 |
| ***LY6G6C*** | rs973716 | 11 | 87897830 | trans | 9.61E-06 | 1.20E-02 | -0.05 | Geneset #1 |
| ***LY6G6C*** | rs1805105 | 16 | 336265 | trans | 9.50E-06 | 8.80E-03 | -0.06 | Geneset #2 |
