# Supplementary material for: Integration of summary data from GWAS and eQTL studies identified novel risk genes for coronary artery disease
Source: Medicine (Baltimore). 2021 Mar 19;100(11):e24769. doi: 10.1097/MD.0000000000024769 (PMC7982177; doi:10.1097/MD.0000000000024769)
Supplement: Supplemental Digital Content [file medi-100-e24769-s011.docx]

**Supplemental Table S16. The co-expression analysis of the 4 identified genes based on the Pearson correlation method in healthy controls**

|  | *CHCHD1* | *TUBG1* | *LY6G6C* | *MRPS17* |
| --- | --- | --- | --- | --- |
| *CHCHD1* | 1.00 | 0.66 | -0.39 | 0.17 |
| *TUBG1* | 0.66 | 1.00 | -0.80 | -0.01 |
| *LY6G6C* | -0.39 | -0.80 | 1.00 | 0.08 |
| *MRPS17* | 0.17 | -0.01 | 0.08 | 1.00 |
