# Supplementary material for: Integration of summary data from GWAS and eQTL studies identified novel risk genes for coronary artery disease
Source: Medicine (Baltimore). 2021 Mar 19;100(11):e24769. doi: 10.1097/MD.0000000000024769 (PMC7982177; doi:10.1097/MD.0000000000024769)
Supplement: Supplemental Digital Content [file medi-100-e24769-s012.docx]

**Supplemental Table S2. Significant KEGG pathways enriched by CAD-associated genes identified from Sherlock Bayesian analysis**

| **ID** | **KEGG ID** | **KEGG Name** | **Enriched P-value** | **Proportion of associated genes (%)** | **Number of associated genes** | **Identified CAD-associated Genes** |
| --- | --- | --- | --- | --- | --- | --- |
| 1 | KEGG:03040 | Spliceosome | 0.0007 | 10.45 | 14.00 | *ALYREF, CDC40, EIF4A3, HNRNPU, PPIE, PRPF38B, SF3A3, SF3B2, SRSF1, SRSF8, SYF2, TCERG1, USP39, ZMAT2* |
| 2 | KEGG:00510 | N-Glycan biosynthesis | 0.0027 | 14.29 | 7.00 | *B4GALT3, DOLPP1, DPM2, MAN1A1, MAN2A1, MAN2A2, STT3A* |
| 3 | KEGG:03015 | mRNA surveillance pathway | 0.0028 | 10.99 | 10.00 | *ALYREF, CPSF2, EIF4A3, ETF1, MSI1, NUDT21, PABPC4, PPP1CA, PPP2R1A, PPP2R2D* |
| 4 | KEGG:04140 | Autophagy | 0.0042 | 9.38 | 12.00 | *ATG3, BAD, DAPK2, GABARAP, IGBP1, IRS2, NRAS, PDPK1, PIK3CA, RB1CC1, TSC1, VMP1* |
| 5 | KEGG:05140 | Leishmaniasis | 0.0080 | 10.81 | 8.00 | *C3, CR1, CYBA, FCGR2C, HLA-DRA, MARCKSL1, MYD88, NFKBIB* |
| 6 | KEGG:00563 | Glycosylphosphatidylinositol (GPI)-anchor biosynthesis | 0.015 | 16.00 | 4.00 | *DPM2, PIGA, PIGC, PIGH* |
| 7 | KEGG:04066 | HIF-1 signaling pathway | 0.016 | 9.00 | 9.00 | *EIF4EBP1, ENO1, GAPDH, HMOX1, LDHA, PFKFB3, PIK3CA, SLC2A1, TIMP1* |
| 8 | KEGG:05120 | Epithelial cell signaling in Helicobacter pylori infection | 0.016 | 10.29 | 7.00 | *ADAM10, ADAM17, ATP6V0A2, ATP6V0E1, ATP6V1B1, PAK1, PTPN11* |
| 9 | KEGG:05211 | Renal cell carcinoma | 0.018 | 10.14 | 7.00 | *BAD, NRAS, PAK1, PIK3CA, PTPN11, SLC2A1, TFE3* |
| 10 | KEGG:05150 | Staphylococcus aureus infection | 0.021 | 10.71 | 6.00 | *C3, C5AR1, CFB, CFI, FCGR2C, HLA-DRA* |
| 11 | KEGG:04211 | Longevity regulating pathway | 0.023 | 8.99 | 8.00 | *EHMT1, EIF4EBP1, IRS2, NRAS, PIK3CA, RB1CC1, SESN1, TSC1* |
| 12 | KEGG:04722 | Neurotrophin signaling pathway | 0.026 | 8.40 | 10.00 | *BAD, MAGED1, MAP3K1, NFKBIB, NRAS, PDPK1, PIK3CA, PTPN11, RPS6KA5, SHC3* |
| 13 | KEGG:04137 | Mitophagy | 0.041 | 9.23 | 6.00 | *GABARAP, NRAS, RHOT1, SQSTM1, TFE3, UBB* |
| 14 | KEGG:05221 | Acute myeloid leukemia | 0.044 | 9.09 | 6.00 | *BAD, BCL2A1, EIF4EBP1, NRAS, PIK3CA, PIM2* |
| 15 | KEGG:05010 | Alzheimer's disease | 0.044 | 7.02 | 12.00 | *ADAM10, ADAM17, ATP5G2, BAD, CDK5, CYC1, GAPDH, NDUFA4, NDUFV2, RTN3, SDHD, UQCRFS1* |
| 16 | KEGG:04360 | Axon guidance | 0.048 | 6.86 | 12.00 | *CDK5, CFL1, GNAI2, LIMK2, NRAS, PAK1, PIK3CA, PTPN11, RASA1, SEMA3F, SEMA6B, SHH* |
| 17 | KEGG:04145 | Phagosome | 0.052 | 7.24 | 11.00 | *ATP6V0A2, ATP6V0E1, ATP6V1B1, C3, CYBA, DYNC1LI2, FCGR2C, HLA-DRA, RAB5A, RAB5B, SEC61B* |
| 18 | KEGG:03010 | Ribosome | 0.054 | 7.14 | 11.00 | *MRPL22, MRPS17, MRPS18A, RPL15, RPL22, RPL27A, RPL39, RPL7, RPL9, RPS15, RPS19* |
| 19 | KEGG:04152 | AMPK signaling pathway | 0.055 | 7.44 | 9.00 | *CRTC2, EIF4EBP1, IRS2, PDPK1, PFKFB3, PIK3CA, PPP2R1A, PPP2R2D, TSC1* |
| 20 | KEGG:03060 | Protein export | 0.059 | 13.04 | 3.00 | *SEC61B, SPCS2, SRP14* |
| 21 | KEGG:05142 | Chagas disease (American trypanosomiasis) | 0.062 | 7.84 | 8.00 | *C3, CCL3L1, CCL3L3, GNAI2, MYD88, PIK3CA, PPP2R1A, PPP2R2D* |
| 22 | KEGG:05166 | HTLV-I infection | 0.067 | 6.27 | 16.00 | *ANAPC11, CRTC2, DLG1, DVL2, HLA-DRA, MAD2L1, MAP3K1, NRAS, PIK3CA, RB1, SLC2A1, TBPL1, VAC14, WNT16, WNT2, XPO1* |
| 23 | KEGG:04910 | Insulin signaling pathway | 0.070 | 7.25 | 10.00 | *BAD, EIF4EBP1, IRS2, NRAS, PDPK1, PIK3CA, PPP1CA, PRKAR2B, SHC3, TSC1* |
| 24 | KEGG:04931 | Insulin resistance | 0.071 | 7.48 | 8.00 | *CRTC2, IRS2, PDPK1, PIK3CA, PPP1CA, PTPN11, SLC27A1, SLC2A1* |
| 25 | KEGG:05219 | Bladder cancer | 0.075 | 9.76 | 4.00 | *DAPK2, NRAS, RB1, RPS6KA5* |
| 26 | KEGG:00790 | Folate biosynthesis | 0.079 | 11.54 | 3.00 | *DHFR2, MOCS1, PTS* |
| 27 | KEGG:00310 | Lysine degradation | 0.080 | 8.47 | 5.00 | *ACAT2, EHMT1, KMT2E, NSD3, PIPOX* |
| 28 | KEGG:04012 | ErbB signaling pathway | 0.082 | 8.14 | 7.00 | *BAD, EIF4EBP1, EREG, NRAS, PAK1, PIK3CA, SHC3* |
| 29 | KEGG:04966 | Collecting duct acid secretion | 0.087 | 11.11 | 3.00 | *ATP6V0A2, ATP6V0E1, ATP6V1B1* |
