# Supplementary material for: Integration of summary data from GWAS and eQTL studies identified novel risk genes for coronary artery disease
Source: Medicine (Baltimore). 2021 Mar 19;100(11):e24769. doi: 10.1097/MD.0000000000024769 (PMC7982177; doi:10.1097/MD.0000000000024769)
Supplement: Supplemental Digital Content [file medi-100-e24769-s013.docx]

**Supplemental Table S17. The co-expression analysis of the 4 identified genes based on the Pearson correlation method in CAD patients**

|  | *CHCHD1* | *TUBG1* | *LY6G6C* | *MRPS17* |
| --- | --- | --- | --- | --- |
| *CHCHD1* | 1.00 | 0.46 | -0.76 | -0.09 |
| *TUBG1* | 0.46 | 1.00 | -0.58 | -0.35 |
| *LY6G6C* | -0.76 | -0.58 | 1.00 | -0.06 |
| *MRPS17* | -0.09 | -0.35 | -0.06 | 1.00 |
