# Supplementary material for: Integration of summary data from GWAS and eQTL studies identified novel risk genes for coronary artery disease
Source: Medicine (Baltimore). 2021 Mar 19;100(11):e24769. doi: 10.1097/MD.0000000000024769 (PMC7982177; doi:10.1097/MD.0000000000024769)
Supplement: Supplemental Digital Content [file medi-100-e24769-s015.docx]

**Supplemental Table S3. Significant Wiki pathways enriched by CAD-associated genes identified from Sherlock Bayesian analysis**

| **ID** | **Wiki Pathway ID** | **Wiki Pathway Name** | **Enriched P-value** | **Proportion of associated genes (%)** | **Number of associated genes** |
| --- | --- | --- | --- | --- | --- |
| 1 | WP:2380 | Brain-Derived Neurotrophic Factor (BDNF) signaling pathway | 6.26E-04 | 9.72 | 14 |
| 2 | WP:3969 | H19 action Rb-E2F1 signaling and CDK-catenin activity | 1.59E-03 | 26.67 | 4 |
| 3 | WP:411 | mRNA Processing | 1.92E-03 | 9.45 | 12 |
| 4 | WP:1946 | Cori Cycle | 2.06E-03 | 25.00 | 4 |
| 5 | WP:3844 | PI3K-AKT-mTOR signaling pathway and therapeutic opportunities | 3.84E-03 | 16.67 | 5 |
| 6 | WP:481 | Insulin Signaling | 7.65E-03 | 8.13 | 13 |
| 7 | WP:3303 | Rac1/Pak1/p38/MMP-2 pathway | 9.62E-03 | 10.45 | 7 |
| 8 | WP:585 | Interferon type I signaling pathways | 1.21E-02 | 11.11 | 6 |
| 9 | WP:195 | IL-1 signaling pathway | 1.32E-02 | 10.91 | 6 |
| 10 | WP:477 | Cytoplasmic Ribosomal Proteins | 1.39E-02 | 8.99 | 8 |
| 11 | WP:3 | Transcriptional activation by NRF2 | 1.49E-02 | 20.00 | 3 |
| 12 | WP:313 | Signaling of Hepatocyte Growth Factor Receptor | 3.18E-02 | 11.76 | 4 |
| 13 | WP:395 | IL-4 Signaling Pathway | 4.27E-02 | 9.26 | 5 |
| 14 | WP:2509 | Nanoparticle triggered autophagic cell death | 4.70E-02 | 13.04 | 3 |
| 15 | WP:2526 | PDGF Pathway | 4.91E-02 | 10.26 | 4 |
| 16 | WP:437 | EGF/EGFR Signaling Pathway | 5.00E-02 | 6.75 | 11 |
