# Supplementary material for: Integration of summary data from GWAS and eQTL studies identified novel risk genes for coronary artery disease
Source: Medicine (Baltimore). 2021 Mar 19;100(11):e24769. doi: 10.1097/MD.0000000000024769 (PMC7982177; doi:10.1097/MD.0000000000024769)
Supplement: Supplemental Digital Content [file medi-100-e24769-s016.docx]

**Supplemental Table S4. Significant Reactome pathways enriched by CAD-associated genes identified from Sherlock Bayesian analysis**

| **ID** | **Reactome ID** | **Reactome Name** | **Enriched P-value** | **Proportion of associated genes (%)** | **Number of associated genes** |
| --- | --- | --- | --- | --- | --- |
| 1 | R-HSA:3266557 | Factor I cleaves iC3b | 4.51E-05 | 100.00 | 3 |
| 2 | R-HSA:162730 | phosphatidylinositol + UDP-N-acetyl-D-glucosamine -> N-acetylglucosaminyl-PI + UDP | 5.13E-05 | 57.14 | 4 |
| 3 | R-HSA:72766 | Translation | 6.82E-05 | 8.56 | 25 |
| 4 | R-HSA:8953854 | Metabolism of RNA | 1.03E-04 | 6.50 | 44 |
| 5 | R-HSA:1799339 | SRP-dependent cotranslational protein targeting to membrane | 1.88E-04 | 11.50 | 13 |
| 6 | R-HSA:1799329 | Signal peptidase hydrolyzes signal peptide from ribosome-associated nascent protein | 3.52E-04 | 11.43 | 12 |
| 7 | R-HSA:72143 | Lariat Formation and 5'-Splice Site Cleavage | 3.70E-04 | 9.80 | 15 |
| 8 | R-HSA:156661 | Formation of Exon Junction Complex | 4.55E-04 | 9.62 | 15 |
| 9 | R-HSA:1799326 | Signal-containing nascent peptide translocates to endoplasmic reticulum | 4.56E-04 | 11.11 | 12 |
| 10 | R-HSA:72172 | mRNA Splicing | 4.82E-04 | 8.90 | 17 |
| 11 | R-HSA:392499 | Metabolism of proteins | 6.22E-04 | 4.90 | 100 |
| 12 | R-HSA:72160 | Cleavage at the 3'-Splice Site and Exon Ligation | 6.36E-04 | 9.32 | 15 |
| 13 | R-HSA:2122948 | Activated NOTCH1 Transmits Signal to the Nucleus | 6.85E-04 | 19.35 | 6 |
| 14 | R-HSA:177929 | Signaling by EGFR | 7.40E-04 | 16.28 | 7 |
| 15 | R-HSA:72163 | mRNA Splicing - Major Pathway | 8.51E-04 | 8.74 | 16 |
| 16 | R-HSA:927802 | Nonsense-Mediated Decay (NMD) | 8.71E-04 | 10.34 | 12 |
| 17 | R-HSA:927813 | p-4S-UPF1 recruits SMG5, SMG7, SMG6, PNRC2, DCP1A, and PP2A | 8.71E-04 | 10.34 | 12 |
| 18 | R-HSA:975957 | Nonsense Mediated Decay (NMD) enhanced by the Exon Junction Complex (EJC) | 8.71E-04 | 10.34 | 12 |
| 19 | R-HSA:75109 | Triglyceride biosynthesis | 8.82E-04 | 30.77 | 4 |
| 20 | R-HSA:72139 | Formation of the active Spliceosomal C (B*) complex | 9.38E-04 | 9.33 | 14 |
| 21 | R-HSA:72130 | Formation of an intermediate Spliceosomal C (Bact) complex | 9.88E-04 | 8.93 | 15 |
| 22 | R-HSA:6798743 | Exocytosis of secretory granule membrane proteins | 1.21E-03 | 12.16 | 9 |
| 23 | R-HSA:72203 | Processing of Capped Intron-Containing Pre-mRNA | 1.27E-03 | 7.82 | 19 |
| 24 | R-HSA:977375 | CR1 binds C3bBb/C4bC2a | 1.42E-03 | 42.86 | 3 |
| 25 | R-HSA:977602 | Complement factor I binds to MCP, CR1:C4b, C3b | 1.42E-03 | 42.86 | 3 |
| 26 | R-HSA:977615 | Factor I inactivates MCP/CR1-bound C4b/C3b | 1.42E-03 | 42.86 | 3 |
| 27 | R-HSA:977629 | Displacement of C2a/Bb by CR1 | 1.42E-03 | 42.86 | 3 |
| 28 | R-HSA:166520 | Signalling by NGF | 1.49E-03 | 8.57 | 15 |
| 29 | R-HSA:2691230 | Signaling by NOTCH1 HD Domain Mutants in Cancer | 1.59E-03 | 26.67 | 4 |
| 30 | R-HSA:2691232 | Constitutive Signaling by NOTCH1 HD Domain Mutants | 1.59E-03 | 26.67 | 4 |
| 31 | R-HSA:927836 | SMG6 hydrolyzes mRNA with premature termination codon | 2.00E-03 | 9.91 | 11 |
| 32 | R-HSA:1660516 | Synthesis of PIPs at the early endosome membrane | 2.06E-03 | 25.00 | 4 |
| 33 | R-HSA:5362798 | Release of Hh-Np from the secreting cell | 2.21E-03 | 37.50 | 3 |
| 34 | R-HSA:168254 | Influenza Infection | 2.47E-03 | 8.43 | 14 |
| 35 | R-HSA:162710 | Synthesis of glycosylphosphatidylinositol (GPI) | 3.28E-03 | 22.22 | 4 |
| 36 | R-HSA:2408522 | Selenoamino acid metabolism | 3.46E-03 | 9.24 | 11 |
| 37 | R-HSA:168255 | Influenza Life Cycle | 3.64E-03 | 8.39 | 13 |
| 38 | R-HSA:5690790 | Histone H2A is dubiquitinated by the PR-DUB complex | 3.84E-03 | 16.67 | 5 |
| 39 | R-HSA:9014610 | Translation of ROBO3.2 mRNA initiates NMD | 3.93E-03 | 9.62 | 10 |
| 40 | R-HSA:9014652 | Translation of ROBO3.2 mRNA is negatively regulated by NMD | 3.93E-03 | 9.62 | 10 |
| 41 | R-HSA:1483257 | Phospholipid metabolism | 4.27E-03 | 7.55 | 16 |
| 42 | R-HSA:141671 | Polypeptide release from the eRF3-GDP:eRF1:mRNA:80S Ribosome complex | 5.07E-03 | 9.89 | 9 |
| 43 | R-HSA:141673 | GTP Hydrolysis by eRF3 bound to the eRF1:mRNA:polypeptide:80S Ribosome complex | 5.07E-03 | 9.89 | 9 |
| 44 | R-HSA:141691 | GTP bound eRF3:eRF1 complex binds the peptidyl tRNA:mRNA:80S Ribosome complex | 5.07E-03 | 9.89 | 9 |
| 45 | R-HSA:74752 | Signaling by Insulin receptor | 5.11E-03 | 10.67 | 8 |
| 46 | R-HSA:927832 | UPF1 binds an mRNP with a termination codon preceding an Exon Junction Complex | 5.15E-03 | 9.26 | 10 |
| 47 | R-HSA:927889 | SMG1 phosphorylates UPF1 (enhanced by Exon Junction Complex) | 5.15E-03 | 9.26 | 10 |
| 48 | R-HSA:9010553 | Regulation of expression of SLITs and ROBOs | 5.26E-03 | 8.14 | 14 |
| 49 | R-HSA:68886 | M Phase | 5.28E-03 | 6.33 | 25 |
| 50 | R-HSA:5218921 | VEGFR2 mediated cell proliferation | 5.89E-03 | 19.05 | 4 |
| 51 | R-HSA:199991 | Membrane Trafficking | 5.93E-03 | 5.63 | 36 |
| 52 | R-HSA:157632 | Complex of NOTCH1 with its ligand is cleaved to produce NEXT1 | 6.00E-03 | 27.27 | 3 |
| 53 | R-HSA:2220944 | ADAM10/17 cleaves ligand-bound NOTCH1 PEST domain mutants to produce NEXT1 PEST domain mutants | 6.00E-03 | 27.27 | 3 |
| 54 | R-HSA:2220976 | NOTCH1 HD+PEST domain mutants are cleaved by ADAM10/17 irrespective of ligand binding | 6.00E-03 | 27.27 | 3 |
| 55 | R-HSA:2730752 | NOTCH1 HD domain mutants are cleaved to produce NEXT1 irrespective of ligand binding | 6.00E-03 | 27.27 | 3 |
| 56 | R-HSA:157118 | Signaling by NOTCH | 6.24E-03 | 9.01 | 10 |
| 57 | R-HSA:72124 | Formation of the Spliceosomal A Complex | 6.24E-03 | 9.01 | 10 |
| 58 | R-HSA:1799332 | Nascent polypeptide:mRNA:ribosome complex binds signal recognition particle (SRP) | 6.27E-03 | 9.57 | 9 |
| 59 | R-HSA:72764 | Eukaryotic Translation Termination | 6.27E-03 | 9.57 | 9 |
| 60 | R-HSA:72127 | Formation of the Spliceosomal B Complex | 6.33E-03 | 8.16 | 12 |
| 61 | R-HSA:1799330 | The SRP receptor binds the SRP:nascent peptide:ribosome complex | 7.18E-03 | 9.38 | 9 |
| 62 | R-HSA:927789 | Formation of UPF1:eRF3 complex on mRNA with a premature termination codon and no Exon Junction Complex | 7.18E-03 | 9.38 | 9 |
| 63 | R-HSA:975956 | Nonsense Mediated Decay (NMD) independent of the Exon Junction Complex (EJC) | 7.18E-03 | 9.38 | 9 |
| 64 | R-HSA:5579029 | Metabolic disorders of biological oxidation enzymes | 7.57E-03 | 14.29 | 5 |
| 65 | R-HSA:5368279 | MTIF3 binds 28S ribosomal subunit | 7.57E-03 | 14.29 | 5 |
| 66 | R-HSA:1643685 | Disease | 8.04E-03 | 5.06 | 56 |
| 67 | R-HSA:5389849 | 28S subunit:MTIF3 binds MTIF2:GTP, mRNA, and formylMet-tRNA | 8.54E-03 | 13.89 | 5 |
| 68 | R-HSA:192841 | Viral Protein Synthesis | 9.31E-03 | 9.00 | 9 |
| 69 | R-HSA:8979227 | Triglyceride metabolism | 9.59E-03 | 13.51 | 5 |
| 70 | R-HSA:1483213 | Synthesis of PE | 9.86E-03 | 23.08 | 3 |
| 71 | R-HSA:209543 | p75NTR recruits signalling complexes | 9.86E-03 | 23.08 | 3 |
| 72 | R-HSA:192823 | Viral mRNA Translation | 9.91E-03 | 8.91 | 9 |
| 73 | R-HSA:983169 | Class I MHC mediated antigen processing & presentation | 1.00E-02 | 6.17 | 23 |
| 74 | R-HSA:9006934 | Signaling by Receptor Tyrosine Kinases | 1.02E-02 | 5.79 | 30 |
| 75 | R-HSA:1660499 | Synthesis of PIPs at the plasma membrane | 1.11E-02 | 11.32 | 6 |
| 76 | R-HSA:156826 | Dissociation of L13a from the 60s ribosomal subunit | 1.11E-02 | 11.32 | 6 |
| 77 | R-HSA:72689 | Formation of a pool of free 40S subunits | 1.12E-02 | 8.74 | 9 |
| 78 | R-HSA:72613 | Eukaryotic Translation Initiation | 1.12E-02 | 8.26 | 10 |
| 79 | R-HSA:72737 | Cap-dependent Translation Initiation | 1.12E-02 | 8.26 | 10 |
| 80 | R-HSA:400253 | Circadian Clock | 1.21E-02 | 10.00 | 7 |
| 81 | R-HSA:180024 | DARPP-32 events | 1.28E-02 | 15.38 | 4 |
| 82 | R-HSA:156912 | Peptide transfer from P-site tRNA to the A-site tRNA | 1.31E-02 | 9.09 | 8 |
| 83 | R-HSA:1799335 | Synthesis of nascent polypeptide containing signal sequence | 1.31E-02 | 9.09 | 8 |
| 84 | R-HSA:1655829 | Regulation of cholesterol biosynthesis by SREBP (SREBF) | 1.32E-02 | 10.91 | 6 |
| 85 | R-HSA:5654741 | Signaling by FGFR3 | 1.33E-02 | 12.50 | 5 |
| 86 | R-HSA:1430728 | Metabolism | 1.34E-02 | 4.50 | 94 |
| 87 | R-HSA:5687128 | MAPK6/MAPK4 signaling | 1.39E-02 | 8.99 | 8 |
| 88 | R-HSA:156907 | Aminoacyl-tRNA binds to the ribosome at the A-site | 1.39E-02 | 8.99 | 8 |
| 89 | R-HSA:156923 | Hydrolysis of eEF1A:GTP | 1.39E-02 | 8.99 | 8 |
| 90 | R-HSA:192704 | Synthesis of PB1-F2 | 1.39E-02 | 8.99 | 8 |
| 91 | R-HSA:72671 | eIF5B:GTP is hydrolyzed and released | 1.39E-02 | 8.99 | 8 |
| 92 | R-HSA:72672 | The 60S subunit joins the translation initiation complex | 1.39E-02 | 8.99 | 8 |
| 93 | R-HSA:1169408 | ISG15 antiviral mechanism | 1.40E-02 | 9.72 | 7 |
| 94 | R-HSA:1169410 | Antiviral mechanism by IFN-stimulated genes | 1.40E-02 | 9.72 | 7 |
| 95 | R-HSA:388841 | Costimulation by the CD28 family | 1.40E-02 | 9.72 | 7 |
| 96 | R-HSA:72107 | Formation of the Spliceosomal E complex | 1.40E-02 | 9.72 | 7 |
| 97 | R-HSA:5654743 | Signaling by FGFR4 | 1.47E-02 | 12.20 | 5 |
| 98 | R-HSA:156902 | Peptide chain elongation | 1.48E-02 | 8.89 | 8 |
| 99 | R-HSA:156915 | Translocation of ribosome by 3 bases in the 3' direction | 1.48E-02 | 8.89 | 8 |
| 100 | R-HSA:2408529 | Sec-tRNA(Sec):EEFSEC:GTP binds to 80S Ribosome | 1.48E-02 | 8.89 | 8 |
| 101 | R-HSA:5333615 | 80S:Met-tRNAi:mRNA:SECISBP2:Sec-tRNA(Sec):EEFSEC:GTP is hydrolysed to 80S:Met-tRNAi:mRNA:SECISBP2:Sec and EEFSEC:GDP by EEFSEC | 1.48E-02 | 8.89 | 8 |
| 102 | R-HSA:72673 | Release of 40S and 60S subunits from the 80S ribosome | 1.48E-02 | 8.89 | 8 |
| 103 | R-HSA:983140 | Transfer of Ub from E2 to substrate and release of E2 | 1.48E-02 | 6.67 | 17 |
| 104 | R-HSA:983156 | Polyubiquitination of substrate | 1.48E-02 | 6.67 | 17 |
| 105 | R-HSA:983157 | Interaction of E3 with substrate and E2-Ub complex | 1.48E-02 | 6.67 | 17 |
| 106 | R-HSA:8875320 | RAB5 GEFs exchange GTP for GDP on RAB5 | 1.49E-02 | 20.00 | 3 |
| 107 | R-HSA:1989750 | Expression of FATP1 (SLC27A1) | 1.49E-02 | 20.00 | 3 |
| 108 | R-HSA:1989759 | Expression of G0S2 | 1.49E-02 | 20.00 | 3 |
| 109 | R-HSA:376176 | Signaling by ROBO receptors | 1.49E-02 | 6.85 | 15 |
| 110 | R-HSA:1980143 | Signaling by NOTCH1 | 1.50E-02 | 9.59 | 7 |
| 111 | R-HSA:2426168 | Activation of gene expression by SREBF (SREBP) | 1.62E-02 | 11.90 | 5 |
| 112 | R-HSA:1483191 | Synthesis of PC | 1.66E-02 | 14.29 | 4 |
| 113 | R-HSA:72180 | Cleavage of mRNA at the 3'-end | 1.68E-02 | 10.34 | 6 |
| 114 | R-HSA:72187 | mRNA 3'-end processing | 1.68E-02 | 10.34 | 6 |
| 115 | R-HSA:8849157 | TREX complex binds spliced, capped mRNA:CBC:EJC cotranscriptionally | 1.68E-02 | 10.34 | 6 |
| 116 | R-HSA:8949664 | Processing of SMDT1 | 1.78E-02 | 18.75 | 3 |
| 117 | R-HSA:917696 | Cargo Sequestration | 1.78E-02 | 18.75 | 3 |
| 118 | R-HSA:917700 | MVB Vesicle Formation | 1.78E-02 | 18.75 | 3 |
| 119 | R-HSA:2559584 | Formation of Senescence-Associated Heterochromatin Foci (SAHF) | 1.78E-02 | 18.75 | 3 |
| 120 | R-HSA:4647594 | SAHF formation | 1.78E-02 | 18.75 | 3 |
| 121 | R-HSA:193639 | p75NTR signals via NF-kB | 1.78E-02 | 18.75 | 3 |
| 122 | R-HSA:1295596 | Spry regulation of FGF signaling | 1.78E-02 | 18.75 | 3 |
| 123 | R-HSA:5653656 | Vesicle-mediated transport | 1.82E-02 | 5.31 | 36 |
| 124 | R-HSA:186763 | Downstream signal transduction | 1.87E-02 | 13.79 | 4 |
| 125 | R-HSA:156842 | Eukaryotic Translation Elongation | 1.89E-02 | 8.51 | 8 |
| 126 | R-HSA:2408557 | Selenocysteine synthesis | 1.89E-02 | 8.51 | 8 |
| 127 | R-HSA:983168 | Antigen processing: Ubiquitination & Proteasome degradation | 1.93E-02 | 6.13 | 19 |
| 128 | R-HSA:1236394 | Signaling by ERBB4 | 1.95E-02 | 11.36 | 5 |
| 129 | R-HSA:156827 | L13a-mediated translational silencing of Ceruloplasmin expression | 1.96E-02 | 7.96 | 9 |
| 130 | R-HSA:168273 | Influenza Viral RNA Transcription and Replication | 1.97E-02 | 7.59 | 11 |
| 131 | R-HSA:8957322 | Metabolism of steroids | 2.02E-02 | 7.53 | 11 |
| 132 | R-HSA:72706 | GTP hydrolysis and joining of the 60S ribosomal subunit | 2.06E-02 | 7.89 | 9 |
| 133 | R-HSA:187037 | NGF signalling via TRKA from the plasma membrane | 2.10E-02 | 8.97 | 7 |
| 134 | R-HSA:8934593 | Regulation of RUNX1 Expression and Activity | 2.11E-02 | 17.65 | 3 |
| 135 | R-HSA:432142 | Platelet sensitization by LDL | 2.11E-02 | 17.65 | 3 |
| 136 | R-HSA:112399 | IRS-mediated signalling | 2.13E-02 | 11.11 | 5 |
| 137 | R-HSA:5358351 | Signaling by Hedgehog | 2.20E-02 | 7.38 | 11 |
| 138 | R-HSA:193704 | p75 NTR receptor-mediated signalling | 2.24E-02 | 8.25 | 8 |
| 139 | R-HSA:8936459 | RUNX1 regulates genes involved in megakaryocyte differentiation and platelet function | 2.24E-02 | 8.25 | 8 |
| 140 | R-HSA:8854214 | TBC/RABGAPs | 2.33E-02 | 10.87 | 5 |
| 141 | R-HSA:210993 | Tie2 Signaling | 2.47E-02 | 16.67 | 3 |
| 142 | R-HSA:5205867 | PRMT1 methylates arginine-12 of histone H2A (H2AR11) | 2.47E-02 | 16.67 | 3 |
| 143 | R-HSA:936964 | Activation of IRF3/IRF7 mediated by TBK1/IKK epsilon | 2.47E-02 | 16.67 | 3 |
| 144 | R-HSA:977606 | Regulation of Complement cascade | 2.53E-02 | 10.64 | 5 |
| 145 | R-HSA:1852241 | Organelle biogenesis and maintenance | 2.56E-02 | 6.06 | 18 |
| 146 | R-HSA:917729 | Endosomal Sorting Complex Required For Transport (ESCRT) | 2.61E-02 | 12.50 | 4 |
| 147 | R-HSA:1222556 | ROS, RNS production in phagocytes | 2.61E-02 | 12.50 | 4 |
| 148 | R-HSA:8873719 | RAB geranylgeranylation | 2.61E-02 | 9.38 | 6 |
| 149 | R-HSA:611105 | Respiratory electron transport | 2.77E-02 | 7.92 | 8 |
| 150 | R-HSA:5358346 | Hedgehog ligand biogenesis | 2.79E-02 | 9.23 | 6 |
| 151 | R-HSA:72202 | Transport of Mature Transcript to Cytoplasm | 2.84E-02 | 8.43 | 7 |
| 152 | R-HSA:1236382 | Constitutive Signaling by Ligand-Responsive EGFR Cancer Variants | 2.85E-02 | 15.79 | 3 |
| 153 | R-HSA:1643713 | Signaling by EGFR in Cancer | 2.85E-02 | 15.79 | 3 |
| 154 | R-HSA:5637815 | Signaling by Ligand-Responsive EGFR Variants in Cancer | 2.85E-02 | 15.79 | 3 |
| 155 | R-HSA:71387 | Metabolism of carbohydrates | 2.96E-02 | 6.20 | 17 |
| 156 | R-HSA:2428928 | IRS-related events triggered by IGF1R | 2.97E-02 | 10.20 | 5 |
| 157 | R-HSA:1483255 | PI Metabolism | 3.01E-02 | 8.33 | 7 |
| 158 | R-HSA:3700989 | Transcriptional Regulation by TP53 | 3.13E-02 | 5.71 | 21 |
| 159 | R-HSA:6798695 | Neutrophil degranulation | 3.17E-02 | 5.43 | 26 |
| 160 | R-HSA:109688 | Cleavage of Growing Transcript in the Termination Region | 3.18E-02 | 8.96 | 6 |
| 161 | R-HSA:73856 | RNA Polymerase II Transcription Termination | 3.18E-02 | 8.96 | 6 |
| 162 | R-HSA:1227986 | Signaling by ERBB2 | 3.21E-02 | 10.00 | 5 |
| 163 | R-HSA:5654736 | Signaling by FGFR1 | 3.21E-02 | 10.00 | 5 |
| 164 | R-HSA:2428924 | IGF1R signaling cascade | 3.21E-02 | 10.00 | 5 |
| 165 | R-HSA:72185 | mRNA polyadenylation | 3.21E-02 | 10.00 | 5 |
| 166 | R-HSA:68877 | Mitotic Prometaphase | 3.26E-02 | 6.50 | 13 |
| 167 | R-HSA:8847635 | Formation of cis-SNARE complex and membrane fusion in intra-Golgi retrograde traffic | 3.27E-02 | 15.00 | 3 |
| 168 | R-HSA:1655848 | Expression of Hydroxymethylglutaryl coenzyme A synthase (HMGCS1) | 3.27E-02 | 15.00 | 3 |
| 169 | R-HSA:5668914 | Diseases of metabolism | 3.39E-02 | 7.62 | 8 |
| 170 | R-HSA:2404192 | Signaling by Type 1 Insulin-like Growth Factor 1 Receptor (IGF1R) | 3.45E-02 | 9.80 | 5 |
| 171 | R-HSA:74751 | Insulin receptor signalling cascade | 3.45E-02 | 9.80 | 5 |
| 172 | R-HSA:2559585 | Oncogene Induced Senescence | 3.49E-02 | 11.43 | 4 |
| 173 | R-HSA:5683057 | MAPK family signaling cascades | 3.63E-02 | 5.90 | 17 |
| 174 | R-HSA:5621575 | CD209 (DC-SIGN) signaling | 3.72E-02 | 14.29 | 3 |
| 175 | R-HSA:8847544 | The COG complex and CUX1 and GOLGA5 dimers contribute to intra-Golgi vesicle tethering | 3.72E-02 | 14.29 | 3 |
| 176 | R-HSA:1655826 | Expression of 3-Hydroxy-3-methylglutaryl-coenzyme A Reductase (HMGCR) | 3.72E-02 | 14.29 | 3 |
| 177 | R-HSA:1655850 | Expression of Farnesyldiphosphate Farnesyltransferase (FDFT1, Squalene Synthase) | 3.72E-02 | 14.29 | 3 |
| 178 | R-HSA:6804757 | Regulation of TP53 Degradation | 3.82E-02 | 11.11 | 4 |
| 179 | R-HSA:177243 | Interactions of Rev with host cellular proteins | 3.82E-02 | 11.11 | 4 |
| 180 | R-HSA:5694446 | BET1:GOSR2:STX5 bind v-SNARES on tethered vesicle | 3.82E-02 | 11.11 | 4 |
| 181 | R-HSA:70171 | Glycolysis | 3.83E-02 | 8.57 | 6 |
| 182 | R-HSA:983147 | Release of E3 from polyubiquitinated substrate | 3.85E-02 | 6.45 | 14 |
| 183 | R-HSA:168256 | Immune System | 3.88E-02 | 4.33 | 92 |
| 184 | R-HSA:75096 | Docking of the TAP:EJC Complex with the NPC | 4.06E-02 | 8.45 | 6 |
| 185 | R-HSA:6806003 | Regulation of TP53 Expression and Degradation | 4.17E-02 | 10.81 | 4 |
| 186 | R-HSA:1176059 | Translocation of Influenza A virus nonstructural protein 1 (NS1A) into the nucleus | 4.17E-02 | 10.81 | 4 |
| 187 | R-HSA:69278 | Cell Cycle, Mitotic | 4.18E-02 | 5.22 | 28 |
| 188 | R-HSA:389513 | CTLA4 inhibitory signaling | 4.20E-02 | 13.64 | 3 |
| 189 | R-HSA:2979096 | NOTCH2 Activation and Transmission of Signal to the Nucleus | 4.20E-02 | 13.64 | 3 |
| 190 | R-HSA:8848021 | Signaling by PTK6 | 4.27E-02 | 9.26 | 5 |
| 191 | R-HSA:9006927 | Signaling by Non-Receptor Tyrosine Kinases | 4.27E-02 | 9.26 | 5 |
| 192 | R-HSA:1280218 | Adaptive Immune System | 4.31E-02 | 4.95 | 38 |
| 193 | R-HSA:163200 | Respiratory electron transport, ATP synthesis by chemiosmotic coupling, and heat production by uncoupling proteins. | 4.40E-02 | 7.26 | 9 |
| 194 | R-HSA:6791226 | Major pathway of rRNA processing in the nucleolus and cytosol | 4.52E-02 | 6.38 | 12 |
| 195 | R-HSA:390470 | Association of CCT/TriC with other substrates during biosynthesis (unknown chaperone) | 4.53E-02 | 10.53 | 4 |
| 196 | R-HSA:5663202 | Diseases of signal transduction | 4.55E-02 | 5.60 | 21 |
| 197 | R-HSA:422475 | Axon guidance | 4.57E-02 | 5.19 | 29 |
| 198 | R-HSA:8949215 | Mitochondrial calcium ion transport | 4.70E-02 | 13.04 | 3 |
| 199 | R-HSA:3788724 | Cdh1:APC/C ubiquitinates EHMT1 and EHMT2 | 4.70E-02 | 13.04 | 3 |
| 200 | R-HSA:5690157 | USP16,USP21 deubiquitinate Histone H2A | 4.70E-02 | 13.04 | 3 |
| 201 | R-HSA:1222516 | Intraphagosomal pH is lowered to 5 by V-ATPase | 4.70E-02 | 13.04 | 3 |
| 202 | R-HSA:159236 | Transport of Mature mRNA derived from an Intron-Containing Transcript | 4.82E-02 | 8.11 | 6 |
| 203 | R-HSA:390471 | Association of TriC/CCT with target proteins during biosynthesis | 4.91E-02 | 10.26 | 4 |
| 204 | R-HSA:77594 | Docking of the Mature intronless derived transcript derived mRNA, TAP and Aly/Ref at the NPC | 4.91E-02 | 10.26 | 4 |
| 205 | R-HSA:1483206 | Glycerophospholipid biosynthesis | 5.00E-02 | 6.98 | 9 |
