# Supplementary material for: Integration of summary data from GWAS and eQTL studies identified novel risk genes for coronary artery disease
Source: Medicine (Baltimore). 2021 Mar 19;100(11):e24769. doi: 10.1097/MD.0000000000024769 (PMC7982177; doi:10.1097/MD.0000000000024769)
Supplement: Supplemental Digital Content [file medi-100-e24769-s017.docx]

**Supplemental Table S5. Significant GO-terms of biological process enriched by CAD-associated genes identified from Sherlock Bayesian analysis**

| **ID** | **GO-Terms ID** | **GO-Terms Name** | **Enriched P-value** | **Proportion of associated genes (%)** | **Number of associated genes** |
| --- | --- | --- | --- | --- | --- |
| 1 | GO:0016071 | mRNA metabolic process | 2.94E-06 | 6.52 | 55 |
| 2 | GO:0010467 | Gene expression | 6.06E-06 | 4.28 | 239 |
| 3 | GO:0006396 | RNA processing | 1.47E-05 | 6.01 | 59 |
| 4 | GO:0006401 | RNA catabolic process | 4.75E-05 | 7.59 | 30 |
| 5 | GO:0016236 | Macroautophagy | 4.90E-05 | 8.28 | 24 |
| 6 | GO:0010608 | Posttranscriptional regulation of gene expression | 5.32E-05 | 6.78 | 37 |
| 7 | GO:0006139 | Nucleobase-containing compound metabolic process | 5.40E-05 | 4.13 | 249 |
| 8 | GO:0034641 | Cellular nitrogen compound metabolic process | 5.45E-05 | 4.07 | 273 |
| 9 | GO:0044249 | Cellular biosynthetic process | 5.68E-05 | 4.10 | 263 |
| 10 | GO:1901576 | Organic substance biosynthetic process | 7.22E-05 | 4.08 | 266 |
| 11 | GO:0031329 | Regulation of cellular catabolic process | 8.53E-05 | 6.02 | 49 |
| 12 | GO:0006402 | mRNA catabolic process | 8.83E-05 | 7.54 | 27 |
| 13 | GO:0006412 | Translation | 9.76E-05 | 6.25 | 44 |
| 14 | GO:0006914 | Autophagy | 1.09E-04 | 6.85 | 34 |
| 15 | GO:1901360 | Organic cyclic compound metabolic process | 1.21E-04 | 4.06 | 262 |
| 16 | GO:0010629 | Negative regulation of gene expression | 1.23E-04 | 4.98 | 92 |
| 17 | GO:0044265 | Cellular macromolecule catabolic process | 1.40E-04 | 5.50 | 61 |
| 18 | GO:0046483 | Heterocycle metabolic process | 1.52E-04 | 4.07 | 252 |
| 19 | GO:0019439 | Aromatic compound catabolic process | 1.56E-04 | 6.45 | 38 |
| 20 | GO:0016070 | RNA metabolic process | 1.61E-04 | 4.22 | 203 |
| 21 | GO:0016241 | Regulation of macroautophagy | 1.67E-04 | 9.58 | 16 |
| 22 | GO:0044248 | Cellular catabolic process | 1.73E-04 | 4.79 | 105 |
| 23 | GO:0006725 | Cellular aromatic compound metabolic process | 1.83E-04 | 4.06 | 253 |
| 24 | GO:0051168 | Nuclear export | 2.29E-04 | 8.70 | 18 |
| 25 | GO:0044267 | Cellular protein metabolic process | 2.37E-04 | 4.14 | 217 |
| 26 | GO:0010506 | Regulation of autophagy | 2.60E-04 | 7.45 | 24 |
| 27 | GO:0034655 | nucleobase-containing compound catabolic process | 3.04E-04 | 6.45 | 34 |
| 28 | GO:0034470 | ncRNA processing | 3.25E-04 | 6.81 | 29 |
| 29 | GO:0034645 | cellular macromolecule biosynthetic process | 3.35E-04 | 4.13 | 216 |
| 30 | GO:0046700 | heterocycle catabolic process | 3.40E-04 | 6.28 | 36 |
| 31 | GO:0006612 | protein targeting to membrane | 3.46E-04 | 8.99 | 16 |
| 32 | GO:0045017 | glycerolipid biosynthetic process | 3.48E-04 | 7.67 | 22 |
| 33 | GO:0044270 | cellular nitrogen compound catabolic process | 3.60E-04 | 6.25 | 36 |
| 34 | GO:0045653 | negative regulation of megakaryocyte differentiation | 3.60E-04 | 60.00 | 3 |
| 35 | GO:1901361 | organic cyclic compound catabolic process | 3.96E-04 | 6.10 | 38 |
| 36 | GO:0009894 | regulation of catabolic process | 4.21E-04 | 5.52 | 53 |
| 37 | GO:0044271 | cellular nitrogen compound biosynthetic process | 4.41E-04 | 4.12 | 212 |
| 38 | GO:0010468 | regulation of gene expression | 4.51E-04 | 4.17 | 197 |
| 39 | GO:0030219 | megakaryocyte differentiation | 4.87E-04 | 13.04 | 9 |
| 40 | GO:0000398 | mRNA splicing, via spliceosome | 5.17E-04 | 7.23 | 24 |
| 41 | GO:0007173 | epidermal growth factor receptor signaling pathway | 5.92E-04 | 9.70 | 13 |
| 42 | GO:0043604 | amide biosynthetic process | 6.52E-04 | 5.63 | 46 |
| 43 | GO:0006793 | phosphorus metabolic process | 7.09E-04 | 4.32 | 149 |
| 44 | GO:0006661 | phosphatidylinositol biosynthetic process | 7.12E-04 | 9.09 | 14 |
| 45 | GO:0006613 | cotranslational protein targeting to membrane | 8.12E-04 | 10.48 | 11 |
| 46 | GO:0031123 | RNA 3'-end processing | 9.05E-04 | 9.76 | 12 |
| 47 | GO:0009057 | macromolecule catabolic process | 9.08E-04 | 5.03 | 68 |
| 48 | GO:0008654 | phospholipid biosynthetic process | 9.29E-04 | 7.31 | 22 |
| 49 | GO:0090407 | organophosphate biosynthetic process | 9.39E-04 | 5.72 | 41 |
| 50 | GO:0006646 | phosphatidylethanolamine biosynthetic process | 9.70E-04 | 28.57 | 4 |
| 51 | GO:0009059 | macromolecule biosynthetic process | 1.02E-03 | 4.05 | 218 |
| 52 | GO:0045047 | protein targeting to ER | 1.11E-03 | 10.09 | 11 |
| 53 | GO:0000056 | ribosomal small subunit export from nucleus | 1.20E-03 | 42.86 | 3 |
| 54 | GO:0006405 | RNA export from nucleus | 1.24E-03 | 8.97 | 13 |
| 55 | GO:1903319 | positive regulation of protein maturation | 1.29E-03 | 20.00 | 5 |
| 56 | GO:1900017 | positive regulation of cytokine production involved in inflammatory response | 1.29E-03 | 26.67 | 4 |
| 57 | GO:0090304 | nucleic acid metabolic process | 1.36E-03 | 4.04 | 215 |
| 58 | GO:0000184 | nuclear-transcribed mRNA catabolic process, nonsense-mediated decay | 1.37E-03 | 9.30 | 12 |
| 59 | GO:0046474 | glycerophospholipid biosynthetic process | 1.40E-03 | 7.34 | 19 |
| 60 | GO:0045652 | regulation of megakaryocyte differentiation | 1.51E-03 | 13.73 | 7 |
| 61 | GO:0006397 | mRNA processing | 1.55E-03 | 6.10 | 31 |
| 62 | GO:0002181 | cytoplasmic translation | 1.61E-03 | 12.12 | 8 |
| 63 | GO:0071428 | rRNA-containing ribonucleoprotein complex export from nucleus | 1.67E-03 | 25.00 | 4 |
| 64 | GO:0055089 | fatty acid homeostasis | 1.67E-03 | 25.00 | 4 |
| 65 | GO:0042886 | amide transport | 1.71E-03 | 4.55 | 98 |
| 66 | GO:0019637 | organophosphate metabolic process | 1.84E-03 | 4.94 | 65 |
| 67 | GO:0072530 | purine-containing compound transmembrane transport | 1.87E-03 | 37.50 | 3 |
| 68 | GO:0006796 | phosphate-containing compound metabolic process | 1.94E-03 | 4.26 | 143 |
| 69 | GO:0031331 | positive regulation of cellular catabolic process | 1.95E-03 | 6.36 | 26 |
| 70 | GO:0006417 | regulation of translation | 1.95E-03 | 6.36 | 26 |
| 71 | GO:0043412 | macromolecule modification | 2.08E-03 | 4.09 | 184 |
| 72 | GO:0006518 | peptide metabolic process | 2.12E-03 | 5.29 | 47 |
| 73 | GO:1903311 | regulation of mRNA metabolic process | 2.18E-03 | 7.04 | 20 |
| 74 | GO:0008104 | protein localization | 2.26E-03 | 4.36 | 121 |
| 75 | GO:0007346 | regulation of mitotic cell cycle | 2.29E-03 | 5.56 | 38 |
| 76 | GO:0015833 | peptide transport | 2.52E-03 | 4.51 | 96 |
| 77 | GO:1903047 | mitotic cell cycle process | 2.53E-03 | 5.31 | 46 |
| 78 | GO:0046488 | phosphatidylinositol metabolic process | 2.60E-03 | 7.33 | 17 |
| 79 | GO:0061013 | regulation of mRNA catabolic process | 2.69E-03 | 7.91 | 14 |
| 80 | GO:0045184 | establishment of protein localization | 2.79E-03 | 4.48 | 98 |
| 81 | GO:0090150 | establishment of protein localization to membrane | 2.96E-03 | 6.76 | 20 |
| 82 | GO:0016331 | morphogenesis of embryonic epithelium | 2.98E-03 | 8.13 | 13 |
| 83 | GO:0008380 | RNA splicing | 3.07E-03 | 6.07 | 27 |
| 84 | GO:0016254 | preassembly of GPI anchor in ER membrane | 3.29E-03 | 21.05 | 4 |
| 85 | GO:0006650 | glycerophospholipid metabolic process | 3.29E-03 | 6.37 | 24 |
| 86 | GO:0046486 | glycerolipid metabolic process | 3.80E-03 | 5.97 | 28 |
| 87 | GO:1901566 | organonitrogen compound biosynthetic process | 3.96E-03 | 4.50 | 92 |
| 88 | GO:0044403 | symbiosis, encompassing mutualism through parasitism | 3.97E-03 | 5.17 | 46 |
| 89 | GO:0051726 | regulation of cell cycle | 4.01E-03 | 4.86 | 60 |
| 90 | GO:0009141 | nucleoside triphosphate metabolic process | 4.02E-03 | 6.34 | 22 |
| 91 | GO:0031124 | mRNA 3'-end processing | 4.06E-03 | 9.68 | 9 |
| 92 | GO:0034613 | cellular protein localization | 4.20E-03 | 4.55 | 85 |
| 93 | GO:2000112 | regulation of cellular macromolecule biosynthetic process | 4.27E-03 | 4.07 | 171 |
| 94 | GO:0033045 | regulation of sister chromatid segregation | 4.29E-03 | 10.39 | 8 |
| 95 | GO:0000278 | mitotic cell cycle | 4.33E-03 | 5.00 | 52 |
| 96 | GO:0006464 | cellular protein modification process | 4.40E-03 | 4.06 | 172 |
| 97 | GO:0044766 | multi-organism transport | 4.64E-03 | 10.26 | 8 |
| 98 | GO:0075733 | intracellular transport of virus | 4.67E-03 | 11.29 | 7 |
| 99 | GO:0006413 | translational initiation | 4.76E-03 | 7.35 | 15 |
| 100 | GO:0010558 | negative regulation of macromolecule biosynthetic process | 4.78E-03 | 4.67 | 72 |
| 101 | GO:0051252 | regulation of RNA metabolic process | 4.98E-03 | 4.09 | 163 |
| 102 | GO:0051276 | chromosome organization | 4.98E-03 | 4.83 | 59 |
| 103 | GO:0000070 | mitotic sister chromatid segregation | 5.07E-03 | 7.95 | 12 |
| 104 | GO:1901362 | organic cyclic compound biosynthetic process | 5.07E-03 | 4.01 | 188 |
| 105 | GO:0090307 | mitotic spindle assembly | 5.10E-03 | 11.11 | 7 |
| 106 | GO:0035247 | peptidyl-arginine omega-N-methylation | 5.11E-03 | 27.27 | 3 |
| 107 | GO:0009890 | negative regulation of biosynthetic process | 5.16E-03 | 4.58 | 76 |
| 108 | GO:0019432 | triglyceride biosynthetic process | 5.26E-03 | 14.71 | 5 |
| 109 | GO:0071705 | nitrogen compound transport | 5.37E-03 | 4.33 | 106 |
| 110 | GO:0072657 | protein localization to membrane | 5.53E-03 | 5.60 | 31 |
| 111 | GO:0006351 | transcription, DNA-templated | 5.58E-03 | 4.08 | 160 |
| 112 | GO:0034660 | ncRNA metabolic process | 5.64E-03 | 5.47 | 33 |
| 113 | GO:0051640 | organelle localization | 5.72E-03 | 5.41 | 36 |
| 114 | GO:0000819 | sister chromatid segregation | 5.85E-03 | 7.00 | 17 |
| 115 | GO:0006605 | protein targeting | 6.09E-03 | 5.90 | 25 |
| 116 | GO:0043603 | cellular amide metabolic process | 6.34E-03 | 4.89 | 52 |
| 117 | GO:0000075 | cell cycle checkpoint | 6.37E-03 | 6.85 | 17 |
| 118 | GO:0043488 | regulation of mRNA stability | 6.54E-03 | 7.69 | 12 |
| 119 | GO:0051883 | killing of cells in other organism involved in symbiotic interaction | 6.75E-03 | 17.39 | 4 |
| 120 | GO:0051225 | spindle assembly | 6.97E-03 | 8.91 | 9 |
| 121 | GO:0016032 | viral process | 7.04E-03 | 5.12 | 42 |
| 122 | GO:0017148 | negative regulation of translation | 7.11E-03 | 6.94 | 15 |
| 123 | GO:0022613 | ribonucleoprotein complex biogenesis | 7.29E-03 | 5.67 | 28 |
| 124 | GO:0031018 | endocrine pancreas development | 7.74E-03 | 11.54 | 6 |
| 125 | GO:0031016 | pancreas development | 7.77E-03 | 9.41 | 8 |
| 126 | GO:1903076 | regulation of protein localization to plasma membrane | 7.77E-03 | 9.41 | 8 |
| 127 | GO:0051090 | regulation of DNA binding transcription factor activity | 7.86E-03 | 5.87 | 24 |
| 128 | GO:2000406 | positive regulation of T cell migration | 7.89E-03 | 16.67 | 4 |
| 129 | GO:0009147 | pyrimidine nucleoside triphosphate metabolic process | 7.89E-03 | 16.67 | 4 |
| 130 | GO:0022402 | cell cycle process | 8.03E-03 | 4.64 | 64 |
| 131 | GO:0007093 | mitotic cell cycle checkpoint | 8.38E-03 | 7.51 | 13 |
| 132 | GO:1901575 | organic substance catabolic process | 8.38E-03 | 4.36 | 93 |
| 133 | GO:0051984 | positive regulation of chromosome segregation | 9.14E-03 | 16.00 | 4 |
| 134 | GO:0006732 | coenzyme metabolic process | 9.26E-03 | 5.94 | 23 |
| 135 | GO:2000273 | positive regulation of receptor activity | 9.48E-03 | 12.82 | 5 |
| 136 | GO:0042254 | ribosome biogenesis | 9.75E-03 | 6.03 | 21 |
| 137 | GO:0042274 | ribosomal small subunit biogenesis | 9.75E-03 | 9.86 | 7 |
| 138 | GO:0009892 | negative regulation of metabolic process | 1.02E-02 | 4.15 | 124 |
| 139 | GO:0002275 | myeloid cell activation involved in immune response | 1.02E-02 | 5.38 | 32 |
| 140 | GO:0009161 | ribonucleoside monophosphate metabolic process | 1.03E-02 | 5.98 | 21 |
| 141 | GO:0001889 | liver development | 1.04E-02 | 7.53 | 11 |
| 142 | GO:0000212 | meiotic spindle organization | 1.05E-02 | 21.43 | 3 |
| 143 | GO:0006355 | regulation of transcription, DNA-templated | 1.05E-02 | 4.05 | 152 |
| 144 | GO:0006369 | termination of RNA polymerase II transcription | 1.05E-02 | 9.72 | 7 |
| 145 | GO:0060765 | regulation of androgen receptor signaling pathway | 1.05E-02 | 15.38 | 4 |
| 146 | GO:0002444 | myeloid leukocyte mediated immunity | 1.06E-02 | 5.34 | 32 |
| 147 | GO:0009792 | embryo development ending in birth or egg hatching | 1.06E-02 | 5.34 | 32 |
| 148 | GO:0036230 | granulocyte activation | 1.08E-02 | 5.45 | 30 |
| 149 | GO:0002446 | neutrophil mediated immunity | 1.09E-02 | 5.44 | 30 |
| 150 | GO:0006403 | RNA localization | 1.16E-02 | 6.48 | 16 |
| 151 | GO:0006656 | phosphatidylcholine biosynthetic process | 1.17E-02 | 12.20 | 5 |
| 152 | GO:0051186 | cofactor metabolic process | 1.18E-02 | 5.65 | 26 |
| 153 | GO:0010508 | positive regulation of autophagy | 1.19E-02 | 8.18 | 9 |
| 154 | GO:0002532 | production of molecular mediator involved in inflammatory response | 1.20E-02 | 10.53 | 6 |
| 155 | GO:0015991 | ATP hydrolysis coupled proton transport | 1.20E-02 | 14.81 | 4 |
| 156 | GO:0030521 | androgen receptor signaling pathway | 1.21E-02 | 9.46 | 7 |
| 157 | GO:0010564 | regulation of cell cycle process | 1.24E-02 | 5.07 | 38 |
| 158 | GO:0071702 | organic substance transport | 1.25E-02 | 4.15 | 119 |
| 159 | GO:0006996 | organelle organization | 1.25E-02 | 4.02 | 153 |
| 160 | GO:0045892 | negative regulation of transcription, DNA-templated | 1.26E-02 | 4.67 | 56 |
| 161 | GO:0006122 | mitochondrial electron transport, ubiquinol to cytochrome c | 1.27E-02 | 20.00 | 3 |
| 162 | GO:0010243 | response to organonitrogen compound | 1.31E-02 | 4.83 | 47 |
| 163 | GO:0031349 | positive regulation of defense response | 1.32E-02 | 5.52 | 26 |
| 164 | GO:0007051 | spindle organization | 1.35E-02 | 7.27 | 12 |
| 165 | GO:0030177 | positive regulation of Wnt signaling pathway | 1.35E-02 | 7.27 | 12 |
| 166 | GO:0071354 | cellular response to interleukin-6 | 1.37E-02 | 14.29 | 4 |
| 167 | GO:0008645 | hexose transport | 1.38E-02 | 7.58 | 10 |
| 168 | GO:0051253 | negative regulation of RNA metabolic process | 1.40E-02 | 4.57 | 61 |
| 169 | GO:0019083 | viral transcription | 1.41E-02 | 6.81 | 13 |
| 170 | GO:1902115 | regulation of organelle assembly | 1.42E-02 | 7.19 | 12 |
| 171 | GO:0007088 | regulation of mitotic nuclear division | 1.42E-02 | 7.19 | 12 |
| 172 | GO:1905953 | negative regulation of lipid localization | 1.42E-02 | 11.63 | 5 |
| 173 | GO:0045786 | negative regulation of cell cycle | 1.43E-02 | 5.19 | 34 |
| 174 | GO:0009790 | embryo development | 1.44E-02 | 4.79 | 48 |
| 175 | GO:0009117 | nucleotide metabolic process | 1.44E-02 | 4.99 | 39 |
| 176 | GO:0006470 | protein dephosphorylation | 1.48E-02 | 6.34 | 17 |
| 177 | GO:0016242 | negative regulation of macroautophagy | 1.55E-02 | 13.79 | 4 |
| 178 | GO:0060571 | morphogenesis of an epithelial fold | 1.55E-02 | 13.79 | 4 |
| 179 | GO:0006378 | mRNA polyadenylation | 1.56E-02 | 11.36 | 5 |
| 180 | GO:0006364 | rRNA processing | 1.70E-02 | 6.14 | 17 |
| 181 | GO:0010605 | negative regulation of macromolecule metabolic process | 1.72E-02 | 4.14 | 112 |
| 182 | GO:0007176 | regulation of epidermal growth factor-activated receptor activity | 1.74E-02 | 13.33 | 4 |
| 183 | GO:0009299 | mRNA transcription | 1.74E-02 | 13.33 | 4 |
| 184 | GO:0045089 | positive regulation of innate immune response | 1.77E-02 | 5.74 | 21 |
| 185 | GO:0009142 | nucleoside triphosphate biosynthetic process | 1.80E-02 | 8.75 | 7 |
| 186 | GO:0033143 | regulation of intracellular steroid hormone receptor signaling pathway | 1.80E-02 | 8.75 | 7 |
| 187 | GO:0051304 | chromosome separation | 1.80E-02 | 8.75 | 7 |
| 188 | GO:0032793 | positive regulation of CREB transcription factor activity | 1.81E-02 | 17.65 | 3 |
| 189 | GO:0031122 | cytoplasmic microtubule organization | 1.87E-02 | 10.87 | 5 |
| 190 | GO:0044068 | modulation by symbiont of host cellular process | 1.94E-02 | 12.90 | 4 |
| 191 | GO:0009895 | negative regulation of catabolic process | 1.95E-02 | 5.99 | 17 |
| 192 | GO:0008610 | lipid biosynthetic process | 1.99E-02 | 4.97 | 36 |
| 193 | GO:0006511 | ubiquitin-dependent protein catabolic process | 1.99E-02 | 5.19 | 31 |
| 194 | GO:0043312 | neutrophil degranulation | 2.01E-02 | 5.23 | 28 |
| 195 | GO:0033047 | regulation of mitotic sister chromatid segregation | 2.04E-02 | 9.38 | 6 |
| 196 | GO:0019693 | ribose phosphate metabolic process | 2.05E-02 | 5.02 | 33 |
| 197 | GO:0006644 | phospholipid metabolic process | 2.06E-02 | 5.35 | 26 |
| 198 | GO:2000484 | positive regulation of interleukin-8 secretion | 2.12E-02 | 16.67 | 3 |
| 199 | GO:0043434 | response to peptide hormone | 2.17E-02 | 5.44 | 24 |
| 200 | GO:0051091 | positive regulation of DNA binding transcription factor activity | 2.18E-02 | 6.15 | 16 |
| 201 | GO:0002274 | myeloid leukocyte activation | 2.19E-02 | 5.00 | 34 |
| 202 | GO:1903321 | negative regulation of protein modification by small protein conjugation or removal | 2.24E-02 | 6.96 | 11 |
| 203 | GO:0045088 | regulation of innate immune response | 2.26E-02 | 5.39 | 24 |
| 204 | GO:0019915 | lipid storage | 2.34E-02 | 9.09 | 6 |
| 205 | GO:0014032 | neural crest cell development | 2.34E-02 | 9.09 | 6 |
| 206 | GO:0030071 | regulation of mitotic metaphase/anaphase transition | 2.39E-02 | 10.20 | 5 |
| 207 | GO:0006637 | acyl-CoA metabolic process | 2.41E-02 | 7.69 | 8 |
| 208 | GO:0016310 | phosphorylation | 2.41E-02 | 4.15 | 100 |
| 209 | GO:0031468 | nuclear envelope reassembly | 2.46E-02 | 15.79 | 3 |
| 210 | GO:1901991 | negative regulation of mitotic cell cycle phase transition | 2.48E-02 | 6.19 | 14 |
| 211 | GO:1903313 | positive regulation of mRNA metabolic process | 2.50E-02 | 8.96 | 6 |
| 212 | GO:0030902 | hindbrain development | 2.50E-02 | 6.79 | 11 |
| 213 | GO:0008643 | carbohydrate transport | 2.50E-02 | 6.79 | 11 |
| 214 | GO:0045930 | negative regulation of mitotic cell cycle | 2.51E-02 | 5.77 | 18 |
| 215 | GO:0016567 | protein ubiquitination | 2.55E-02 | 4.75 | 41 |
| 216 | GO:0043299 | leukocyte degranulation | 2.57E-02 | 5.12 | 30 |
| 217 | GO:0032886 | regulation of microtubule-based process | 2.62E-02 | 6.50 | 13 |
| 218 | GO:0006517 | protein deglycosylation | 2.65E-02 | 11.76 | 4 |
| 219 | GO:0042558 | pteridine-containing compound metabolic process | 2.65E-02 | 11.76 | 4 |
| 220 | GO:0031324 | negative regulation of cellular metabolic process | 2.67E-02 | 4.10 | 110 |
| 221 | GO:0002218 | activation of innate immune response | 2.68E-02 | 5.70 | 18 |
| 222 | GO:0042058 | regulation of epidermal growth factor receptor signaling pathway | 2.72E-02 | 8.05 | 7 |
| 223 | GO:1901990 | regulation of mitotic cell cycle phase transition | 2.73E-02 | 5.44 | 23 |
| 224 | GO:0045931 | positive regulation of mitotic cell cycle | 2.88E-02 | 7.09 | 10 |
| 225 | GO:0010458 | exit from mitosis | 2.91E-02 | 11.43 | 4 |
| 226 | GO:1902186 | regulation of viral release from host cell | 2.91E-02 | 11.43 | 4 |
| 227 | GO:0046034 | ATP metabolic process | 2.96E-02 | 5.90 | 17 |
| 228 | GO:0006887 | exocytosis | 2.97E-02 | 4.69 | 43 |
| 229 | GO:0033043 | regulation of organelle organization | 2.98E-02 | 4.44 | 57 |
| 230 | GO:0021545 | cranial nerve development | 3.01E-02 | 9.62 | 5 |
| 231 | GO:0030490 | maturation of SSU-rRNA | 3.01E-02 | 9.62 | 5 |
| 232 | GO:0045840 | positive regulation of mitotic nuclear division | 3.01E-02 | 9.62 | 5 |
| 233 | GO:0055086 | nucleobase-containing small molecule metabolic process | 3.02E-02 | 4.76 | 40 |
| 234 | GO:0015931 | nucleobase-containing compound transport | 3.03E-02 | 6.05 | 15 |
| 235 | GO:1901698 | response to nitrogen compound | 3.04E-02 | 4.54 | 50 |
| 236 | GO:0045321 | leukocyte activation | 3.04E-02 | 4.45 | 58 |
| 237 | GO:0006886 | intracellular protein transport | 3.05E-02 | 4.50 | 54 |
| 238 | GO:0002262 | myeloid cell homeostasis | 3.09E-02 | 6.94 | 10 |
| 239 | GO:0000122 | negative regulation of transcription from RNA polymerase II promoter | 3.09E-02 | 4.72 | 40 |
| 240 | GO:0010948 | negative regulation of cell cycle process | 3.19E-02 | 5.57 | 19 |
| 241 | GO:0000045 | autophagosome assembly | 3.20E-02 | 7.78 | 7 |
| 242 | GO:0021697 | cerebellar cortex formation | 3.21E-02 | 14.29 | 3 |
| 243 | GO:0045649 | regulation of macrophage differentiation | 3.21E-02 | 14.29 | 3 |
| 244 | GO:0061157 | mRNA destabilization | 3.21E-02 | 14.29 | 3 |
| 245 | GO:1903077 | negative regulation of protein localization to plasma membrane | 3.21E-02 | 14.29 | 3 |
| 246 | GO:1902600 | hydrogen ion transmembrane transport | 3.22E-02 | 7.27 | 8 |
| 247 | GO:0044772 | mitotic cell cycle phase transition | 3.28E-02 | 5.05 | 29 |
| 248 | GO:0032446 | protein modification by small protein conjugation | 3.30E-02 | 4.62 | 44 |
| 249 | GO:0043122 | regulation of I-kappaB kinase/NF-kappaB signaling | 3.32E-02 | 5.91 | 15 |
| 250 | GO:0032103 | positive regulation of response to external stimulus | 3.37E-02 | 5.69 | 17 |
| 251 | GO:0016569 | covalent chromatin modification | 3.38E-02 | 5.01 | 29 |
| 252 | GO:0045787 | positive regulation of cell cycle | 3.38E-02 | 5.33 | 21 |
| 253 | GO:0032874 | positive regulation of stress-activated MAPK cascade | 3.43E-02 | 6.76 | 10 |
| 254 | GO:1901652 | response to peptide | 3.45E-02 | 5.07 | 26 |
| 255 | GO:0032781 | positive regulation of ATPase activity | 3.47E-02 | 9.26 | 5 |
| 256 | GO:0044093 | positive regulation of molecular function | 3.54E-02 | 4.21 | 79 |
| 257 | GO:0043433 | negative regulation of DNA binding transcription factor activity | 3.62E-02 | 6.67 | 10 |
| 258 | GO:0006479 | protein methylation | 3.62E-02 | 6.45 | 12 |
| 259 | GO:0007063 | regulation of sister chromatid cohesion | 3.63E-02 | 13.64 | 3 |
| 260 | GO:1901185 | negative regulation of ERBB signaling pathway | 3.71E-02 | 9.09 | 5 |
| 261 | GO:2000785 | regulation of autophagosome assembly | 3.79E-02 | 10.53 | 4 |
| 262 | GO:1905898 | positive regulation of response to endoplasmic reticulum stress | 3.79E-02 | 10.53 | 4 |
| 263 | GO:0031347 | regulation of defense response | 3.80E-02 | 4.68 | 39 |
| 264 | GO:0045637 | regulation of myeloid cell differentiation | 3.92E-02 | 6.09 | 14 |
| 265 | GO:0032984 | macromolecular complex disassembly | 3.96E-02 | 5.61 | 18 |
| 266 | GO:0010507 | negative regulation of autophagy | 4.04E-02 | 8.00 | 6 |
| 267 | GO:0033044 | regulation of chromosome organization | 4.06E-02 | 5.56 | 18 |
| 268 | GO:0016180 | snRNA processing | 4.07E-02 | 13.04 | 3 |
| 269 | GO:0043902 | positive regulation of multi-organism process | 4.13E-02 | 6.19 | 12 |
| 270 | GO:1902652 | secondary alcohol metabolic process | 4.17E-02 | 6.45 | 10 |
| 271 | GO:0043123 | positive regulation of I-kappaB kinase/NF-kappaB signaling | 4.21E-02 | 6.15 | 12 |
| 272 | GO:0036503 | ERAD pathway | 4.31E-02 | 7.29 | 7 |
| 273 | GO:1903706 | regulation of hemopoiesis | 4.31E-02 | 5.20 | 23 |
| 274 | GO:0032269 | negative regulation of cellular protein metabolic process | 4.31E-02 | 4.45 | 52 |
| 275 | GO:0006913 | nucleocytoplasmic transport | 4.32E-02 | 5.02 | 25 |
| 276 | GO:0002687 | positive regulation of leukocyte migration | 4.41E-02 | 6.87 | 9 |
| 277 | GO:0051338 | regulation of transferase activity | 4.45E-02 | 4.46 | 48 |
| 278 | GO:0010498 | proteasomal protein catabolic process | 4.46E-02 | 5.15 | 23 |
| 279 | GO:0007339 | binding of sperm to zona pellucida | 4.46E-02 | 10.00 | 4 |
| 280 | GO:0048255 | mRNA stabilization | 4.46E-02 | 10.00 | 4 |
| 281 | GO:0038128 | ERBB2 signaling pathway | 4.46E-02 | 10.00 | 4 |
| 282 | GO:0045742 | positive regulation of epidermal growth factor receptor signaling pathway | 4.46E-02 | 10.00 | 4 |
| 283 | GO:0006695 | cholesterol biosynthetic process | 4.50E-02 | 7.79 | 6 |
| 284 | GO:0000245 | spliceosomal complex assembly | 4.51E-02 | 8.62 | 5 |
| 285 | GO:0035336 | long-chain fatty-acyl-CoA metabolic process | 4.54E-02 | 12.50 | 3 |
| 286 | GO:1903306 | negative regulation of regulated secretory pathway | 4.54E-02 | 12.50 | 3 |
| 287 | GO:0001701 | in utero embryonic development | 4.60E-02 | 5.37 | 18 |
| 288 | GO:0033365 | protein localization to organelle | 4.63E-02 | 4.50 | 45 |
| 289 | GO:0002366 | leukocyte activation involved in immune response | 4.69E-02 | 4.72 | 35 |
| 290 | GO:0006892 | post-Golgi vesicle-mediated transport | 4.73E-02 | 7.14 | 7 |
| 291 | GO:0051172 | negative regulation of nitrogen compound metabolic process | 4.73E-02 | 4.05 | 101 |
| 292 | GO:0010876 | lipid localization | 4.74E-02 | 5.25 | 21 |
| 293 | GO:0007032 | endosome organization | 4.74E-02 | 7.69 | 6 |
| 294 | GO:0034308 | primary alcohol metabolic process | 4.80E-02 | 8.47 | 5 |
| 295 | GO:0006066 | alcohol metabolic process | 4.93E-02 | 5.41 | 19 |
| 296 | GO:0048738 | cardiac muscle tissue development | 4.97E-02 | 5.91 | 12 |
| 297 | GO:0061097 | regulation of protein tyrosine kinase activity | 4.99E-02 | 7.59 | 6 |
