# Supplementary material for: Integration of summary data from GWAS and eQTL studies identified novel risk genes for coronary artery disease
Source: Medicine (Baltimore). 2021 Mar 19;100(11):e24769. doi: 10.1097/MD.0000000000024769 (PMC7982177; doi:10.1097/MD.0000000000024769)
Supplement: Supplemental Digital Content [file medi-100-e24769-s018.docx]

**Supplemental Table S6. Significant GO-terms of cellular component enriched by CAD-associated genes identified from Sherlock Bayesian analysis**

| **ID** | **GO-Terms ID** | **GO-Terms Name** | **Enriched P-value** | **Proportion of associated genes (%)** | **Number of associated genes** |
| --- | --- | --- | --- | --- | --- |
| 1 | GO:0005829 | Cytosol | 3.95E-08 | 4.46 | 228 |
| 2 | GO:0005634 | Nucleus | 6.10E-07 | 4.09 | 297 |
| 3 | GO:0030529 | Intracellular ribonucleoprotein complex | 3.88E-06 | 6.35 | 54 |
| 4 | GO:0031967 | Organelle envelope | 3.62E-05 | 5.46 | 66 |
| 5 | GO:0044428 | Nuclear part | 4.37E-05 | 4.24 | 188 |
| 6 | GO:0000506 | Glycosylphosphatidylinositol-N-acetylglucosaminyltransferase (GPI-GnT) complex | 7.10E-05 | 50.00 | 4 |
| 7 | GO:0031981 | Nuclear lumen | 3.05E-04 | 4.18 | 170 |
| 8 | GO:0016604 | Nuclear body | 5.71E-04 | 5.59 | 43 |
| 9 | GO:0090543 | Flemming body | 9.27E-04 | 20.83 | 5 |
| 10 | GO:0005740 | Mitochondrial envelope | 1.15E-03 | 5.48 | 42 |
| 11 | GO:0031966 | Mitochondrial membrane | 1.22E-03 | 5.53 | 40 |
| 12 | GO:0071013 | Catalytic step 2 spliceosome | 1.36E-03 | 10.20 | 10 |
| 13 | GO:0005681 | Spliceosomal complex | 1.55E-03 | 7.85 | 15 |
| 14 | GO:0005654 | Nucleoplasm | 1.69E-03 | 4.14 | 143 |
| 15 | GO:0043657 | Host cell | 2.57E-03 | 10.96 | 8 |
| 16 | GO:0005743 | Mitochondrial inner membrane | 2.83E-03 | 5.85 | 29 |
| 17 | GO:0030496 | Midbody | 2.87E-03 | 7.93 | 13 |
| 18 | GO:0044429 | Mitochondrial part | 3.23E-03 | 4.90 | 52 |
| 19 | GO:0019866 | Organelle inner membrane | 3.49E-03 | 5.58 | 31 |
| 20 | GO:1990204 | Oxidoreductase complex | 6.56E-03 | 8.74 | 9 |
| 21 | GO:0005741 | Mitochondrial outer membrane | 6.78E-03 | 7.11 | 14 |
| 22 | GO:0005750 | Mitochondrial respiratory chain complex III | 7.76E-03 | 23.08 | 3 |
| 23 | GO:0072686 | Mitotic spindle | 8.03E-03 | 9.09 | 8 |
| 24 | GO:0031090 | Organelle membrane | 8.30E-03 | 4.06 | 122 |
| 25 | GO:0005876 | Spindle microtubule | 8.83E-03 | 10.91 | 6 |
| 26 | GO:0016607 | Nuclear speck | 9.05E-03 | 5.81 | 23 |
| 27 | GO:0044451 | Nucleoplasm part | 9.27E-03 | 4.68 | 53 |
| 28 | GO:0005739 | Mitochondrion | 1.12E-02 | 4.33 | 77 |
| 29 | GO:0070069 | Cytochrome complex | 1.76E-02 | 12.90 | 4 |
| 30 | GO:0022626 | Cytosolic ribosome | 1.86E-02 | 7.38 | 9 |
| 31 | GO:0098800 | Inner mitochondrial membrane protein complex | 1.95E-02 | 7.32 | 9 |
| 32 | GO:0098796 | Membrane protein complex | 2.14E-02 | 4.89 | 34 |
| 33 | GO:0000932 | P-body | 2.23E-02 | 8.14 | 7 |
| 34 | GO:0044455 | Mitochondrial membrane part | 2.56E-02 | 6.37 | 13 |
| 35 | GO:0035770 | Ribonucleoprotein granule | 2.63E-02 | 6.31 | 13 |
| 36 | GO:0005730 | Nucleolus | 2.65E-02 | 4.56 | 45 |
| 37 | GO:0030667 | Secretory granule membrane | 2.66E-02 | 5.54 | 18 |
| 38 | GO:0008287 | Protein serine/threonine phosphatase complex | 2.69E-02 | 9.62 | 5 |
| 39 | GO:0005849 | mRNA cleavage factor complex | 2.97E-02 | 14.29 | 3 |
| 40 | GO:0044445 | Cytosolic part | 3.06E-02 | 5.86 | 15 |
| 41 | GO:0005635 | Nuclear envelope | 3.32E-02 | 5.14 | 24 |
| 42 | GO:0035145 | Exon-exon junction complex | 3.36E-02 | 13.64 | 3 |
| 43 | GO:0098803 | Respiratory chain complex | 3.78E-02 | 7.89 | 6 |
| 44 | GO:0031902 | Late endosome membrane | 3.93E-02 | 6.98 | 9 |
| 45 | GO:1990234 | transferase complex | 4.04E-02 | 4.56 | 36 |
| 46 | GO:0005770 | late endosome | 4.15E-02 | 5.79 | 14 |
| 47 | GO:0005643 | nuclear pore | 4.90E-02 | 7.41 | 6 |
| 48 | GO:0031985 | Golgi cisterna | 4.95E-02 | 6.86 | 7 |
