# Supplementary material for: Integration of summary data from GWAS and eQTL studies identified novel risk genes for coronary artery disease
Source: Medicine (Baltimore). 2021 Mar 19;100(11):e24769. doi: 10.1097/MD.0000000000024769 (PMC7982177; doi:10.1097/MD.0000000000024769)
Supplement: Supplemental Digital Content [file medi-100-e24769-s019.docx]

**Supplemental Table S7. Significant GO-terms of molecular function enriched by CAD-associated genes identified from Sherlock Bayesian analysis**

| **ID** | **GO-Terms ID** | **GO-Terms Name** | **Enriched P-value** | **Proportion of associated genes (%)** | **Number of associated genes** |
| --- | --- | --- | --- | --- | --- |
| 1 | GO:0003723 | RNA Binding | 7.67E-07 | 5.61 | 94 |
| 2 | GO:0008187 | Poly-Pyrimidine Tract Binding | 1.32E-03 | 20.00 | 5 |
| 3 | GO:0019904 | Protein Domain Specific Binding | 1.44E-03 | 5.69 | 41 |
| 4 | GO:0008143 | Poly(A) Binding | 1.71E-03 | 25.00 | 4 |
| 5 | GO:0015924 | Mannosyl-Oligosaccharide Mannosidase Activity | 3.87E-03 | 30.00 | 3 |
| 6 | GO:0003676 | Nucleic Acid Binding | 4.85E-03 | 4.09 | 168 |
| 7 | GO:0019208 | Phosphatase Regulator Activity | 5.56E-03 | 9.28 | 9 |
| 8 | GO:0004721 | Phosphoprotein Phosphatase Activity | 5.86E-03 | 7.57 | 14 |
| 9 | GO:0003727 | Single-Stranded Rna Binding | 6.02E-03 | 9.88 | 8 |
| 10 | GO:0043422 | Protein Kinase B Binding | 6.74E-03 | 25.00 | 3 |
| 11 | GO:0016274 | Protein-Arginine N-Methyltransferase Activity | 6.74E-03 | 25.00 | 3 |
| 12 | GO:0001671 | ATPase Activator Activity | 6.88E-03 | 17.39 | 4 |
| 13 | GO:0003729 | mRNA Binding | 1.06E-02 | 6.86 | 14 |
| 14 | GO:0008170 | N-Methyltransferase Activity | 1.80E-02 | 8.16 | 8 |
| 15 | GO:0016791 | Phosphatase Activity | 1.81E-02 | 6.09 | 17 |
| 16 | GO:0019888 | Protein Phosphatase Regulator Activity | 2.36E-02 | 8.33 | 7 |
| 17 | GO:0042578 | Phosphoric Ester Hydrolase Activity | 2.86E-02 | 5.59 | 21 |
| 18 | GO:0008276 | Protein Methyltransferase Activity | 3.28E-02 | 7.78 | 7 |
| 19 | GO:0019899 | Enzyme Binding | 3.45E-02 | 4.15 | 95 |
| 20 | GO:0051537 | 2 Iron, 2 Sulfur Cluster Binding | 3.68E-02 | 13.64 | 3 |
| 21 | GO:0017025 | Tbp-Class Protein Binding | 4.60E-02 | 12.50 | 3 |
| 22 | GO:0004114 | 3',5'-Cyclic-Nucleotide Phosphodiesterase Activity | 4.60E-02 | 12.50 | 3 |
