# Supplementary material for: Integration of summary data from GWAS and eQTL studies identified novel risk genes for coronary artery disease
Source: Medicine (Baltimore). 2021 Mar 19;100(11):e24769. doi: 10.1097/MD.0000000000024769 (PMC7982177; doi:10.1097/MD.0000000000024769)
Supplement: Supplemental Digital Content [file medi-100-e24769-s020.docx]

**Supplemental Table S8. Significant GO-Terms of immune system enriched by CAD-associated genes identified from Sherlock Bayesian analysis**

| **ID** | **GO-Terms ID** | **GO-Terms Name** | **Enriched P-value** | **Proportion of associated genes (%)** | **Number of associated genes** |
| --- | --- | --- | --- | --- | --- |
| 1 | GO:0045653 | Negative Regulation Of Megakaryocyte Differentiation | 4.97E-04 | 60.00 | 3 |
| 2 | GO:0030219 | Megakaryocyte Differentiation | 9.66E-04 | 13.04 | 9 |
| 3 | GO:0045652 | Regulation Of Megakaryocyte Differentiation | 2.67E-03 | 13.73 | 7 |
| 4 | GO:2000406 | Positive Regulation Of T Cell Migration | 1.14E-02 | 16.67 | 4 |
| 5 | GO:0036230 | Granulocyte Activation | 2.64E-02 | 5.45 | 30 |
| 6 | GO:0002275 | Myeloid Cell Activation Involved In Immune Response | 3.03E-02 | 5.38 | 32 |
| 7 | GO:0002444 | Myeloid Leukocyte Mediated Immunity | 3.11E-02 | 5.34 | 32 |
| 8 | GO:0002446 | Neutrophil Mediated Immunity | 3.50E-02 | 5.44 | 30 |
| 9 | GO:0045089 | Positive Regulation Of Innate Immune Response | 4.11E-02 | 5.74 | 21 |
| 10 | GO:0045649 | Regulation Of Macrophage Differentiation | 4.26E-02 | 14.29 | 3 |
